# Human WRN is an intrinsic inhibitor of progerin, abnormal splicing product of lamin A

**DOI:** 10.1038/s41598-021-88325-1

**Published:** 2021-04-27

**Authors:** So-mi Kang, Min-Ho Yoon, Su-Jin Lee, Jinsook Ahn, Sang Ah Yi, Ki Hong Nam, Soyoung Park, Tae-Gyun Woo, Jung-Hyun Cho, Jaecheol Lee, Nam-Chul Ha, Bum-Joon Park

**Affiliations:** 1 Department of Molecular Biology, Pusan National University, Busan, Republic of Korea; 2 School of Pharmacy, Sungkyunkwan University, Suwon, Kyunggi-Do Republic of Korea; 3 Program in Food Science and Biotechnology, College of Agriculture and Life Sciences, Seoul National University, Seoul, Republic of Korea

**Keywords:** Cell biology, Drug discovery, Molecular biology

## Abstract

Werner syndrome (WRN) is a rare progressive genetic disorder, caused by functional defects in WRN protein and RecQ4L DNA helicase. Acceleration of the aging process is initiated at puberty and the expected life span is approximately the late 50 s. However, a *Wrn*-deficient mouse model does not show premature aging phenotypes or a short life span, implying that aging processes differ greatly between humans and mice. Gene expression analysis of WRN cells reveals very similar results to gene expression analysis of Hutchinson Gilford progeria syndrome (HGPS) cells, suggesting that these human progeroid syndromes share a common pathological mechanism. Here we show that WRN cells also express progerin, an abnormal variant of the lamin A protein. In addition, we reveal that duplicated sequences of human WRN (hWRN) from exon 9 to exon 10, which differ from the sequence of mouse WRN (mWRN), are a natural inhibitor of progerin. Overexpression of hWRN reduced progerin expression and aging features in HGPS cells. Furthermore, the elimination of progerin by siRNA or a progerin-inhibitor (SLC-D011 also called progerinin) can ameliorate senescence phenotypes in WRN fibroblasts and cardiomyocytes, derived from WRN-iPSCs. These results suggest that progerin, which easily accumulates under WRN-deficient conditions, can lead to premature aging in WRN and that this effect can be prevented by SLC-D011.

## Introduction

Werner syndrome (WRN) is a human segmental premature aging disorder characterized by hair graying and loss of hair, alopecia, cataracts, progressive subcutaneous atrophy, calcification and ulceration, mesenchymal neoplasms, short stature due to a limited pubertal growth spurt and an elevated risk of age-associated diseases such as diabetes, osteoporosis, and cardiovascular disorders^1–4^. The disorder is typically recognized at 30–40 years of age, but certain characteristic outcomes can also occur in adolescence and early adulthood^5^. Classical WRN is caused by biallelic inactivating mutations of the *WRN* gene which is located in human chromosome 8^6–8^. The *WRN* gene encodes both a 3′ → 5′ helicase and a 3′ → 5′ exonuclease^9,10^. Since WRN protein functions in the cell as a DNA helicase, accumulation of DNA damage has been suggested to be the reason for senescence^11,12^. Cells derived from WRN patients show increased genomic instability and are hypersensitive to DNA damage-related agents^13,14^. However, some previous studies have shown that inhibitors of WRN helicase activity do not elicit a hypersensitive response in WRN cells^15^ and the drug hypersensitivity-profiles of WRN cells are consistent with involvement of the exonuclease activity^14^. Another study also showed that loss of WRN helicase activity due to homozygous inactivating point mutations does not cause clinical WRN^16^. These results indicate that helicase activity is not indispensable for WRN. Another putative aging related mechanism of WRN is telomere dysregulation^17,18^. In fact, WRN deficiency can promote telomere fusion and recombination^19,20^. However, this event is also observed in *Wrn*-deficient mouse cells^20^, (and *Wrn*-deleted mice have normal phenotypes, similar to those of wild-type mice^21^), indicating that telomere dysregulation is not a direct reason for premature aging. Besides, dysregulated gene expression and DNA methylation due to mutations in the *WRN* gene are also known as hypotheses that contribute to inducing senescence^22,23^.

Although WRN deficiency is a major factor in WRN patients, genetic mutation of lamin A has also been proposed as the causal factor of atypical WRN^24,25^. Thus, we hypothesize that WRN is related to dysfunction of lamin A. Progerin, an abnormal splicing variant of lamin A, is a main cause of Hutchinson Gilford progeria syndrome (HGPS)^26,27^. High expression of progerin, which results from a point mutation in exon 11 that generates abnormal splicing donor sites, induces premature aging from the neonatal stage^28^, whereas progerin expression can also accumulate following a normal physiological aging process without genetic mutations^29,30^. Conversely, the amount of WRN decreases as age increases^31^. In our previous study, we found that progerin strongly binds to wild-type lamin A, creating an abnormal nuclear shape and promoting the aging process^32^. These previous reports show that, defects in lamin A regulation are closely involved in pathological and physiological aging progression. We thought it was likely that a connection between WRN and progerin existed and would affect the aging process. Therefore, we hypothesized that WRN protein is a natural inhibitor of progerin accumulation and that the accumulation of progerin is promoted under WRN-deficient conditions, leading to premature senescence in WRN patients. Thus, in this report, we explored a novel function of WRN in the aging process and introduced an inhibitor of progerin as an effective drug candidate for WRN patients.

## Results

### WRN shows a similar gene expression profile to HGPS

**Figure 1:**
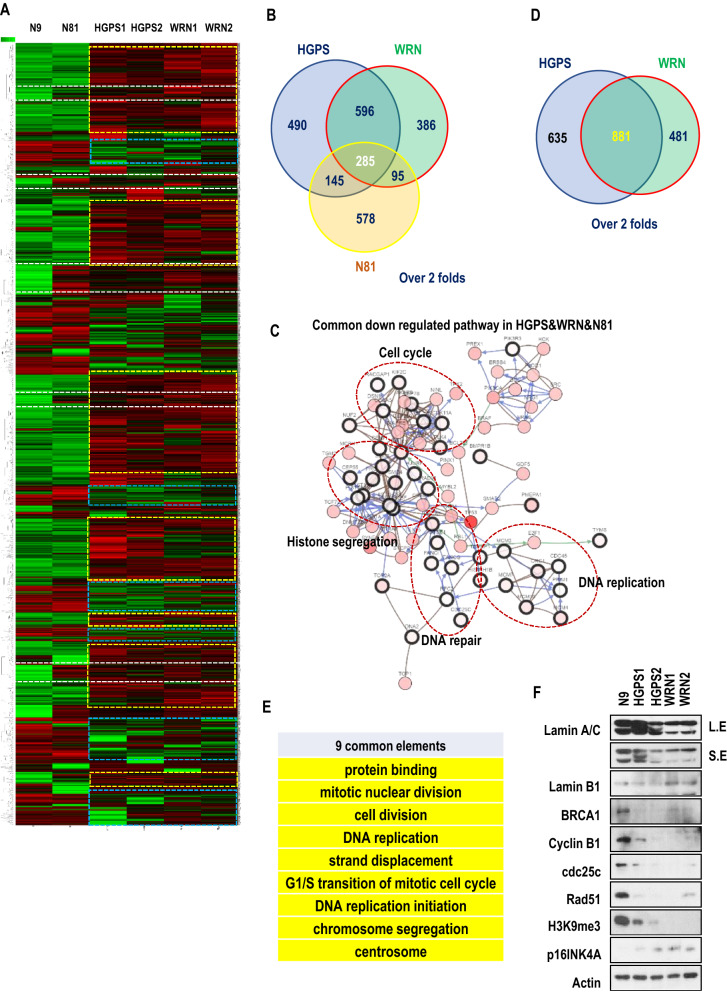
Gene ontology analysis of HGPS and WRN cells. (**A**) Heatmap of gene expression profiles in normal fibroblasts (young; N9; unaffected 9-year old subject, and aged; N81; unaffected 81-year-old subject) and prematurely aged cells (HGPS and WRN) at passage 10. Microarray was performed using Human Gene 1.0 ST arrays (Affymetrix). Yellow boxes indicate gene sets downregulated in both WRN and HGPS cells compared to normal fibroblasts (N9). Blue boxes indicate gene sets upregulated in both WRN and HGPS cells compared to N9 fibroblasts. White boxes indicate gene sets common to N81 fibroblasts as well as WRN and HGPS cells compared to N9 fibroblasts. (**B**) Analysis of differentially expressed genes (DEGs) showing a more than twofold difference from N9 fibroblasts. A total of 285 genes (core gene set) were commonly altered in progeroid cells (WRN and HGPS) and N81 fibroblasts compared to N9 fibroblasts. (**C**) Downregulated genes in the core gene set are associated with the cell cycle, chromosome separation, DNA replication, and DNA repair in HGPS, WRN, and N81 fibroblasts. Gene clustering was performed using the gene interaction mapping tool in cBioPortal (http://www.cbioportal.org). (**D**) DEG analysis (at least twofold) of HGPS and WRN cells compared to N9 fibroblasts. A total of 881 genes were altered in both HGPS and WRN cells. (**E**) Nine common elements overlapped between HGPS and WRN, including cell cycle regulation and chromosome separation. (**F**) Compared to normal fibroblasts (at passage 10), the expression of BRCA1, cyclin B1, cdc25c, rad51, H3K9me3, and p16INK4A in HGPS and WRN cells (at passage 10) was reduced. Western blots were cropped from different parts of the same samples and analyzed by film-based imaging systems (*n* = 3 independent experiments; two-tailed Student’s *t*-test).

To investigate our hypothesis, we first performed microarray to examine the gene expression profile of fibroblasts from HGPS subjects, WRN subjects, and an unaffected old-aged subject (N81; 81 years old) compared to fibroblasts from an unaffected young-aged subject (N9; 9 years old). We found that a large portion of gene sets were commonly upregulated (blue boxes; Fig. 1A) or downregulated (yellow boxes; Fig. 1A) in HGPS and WRN cells compared to normal young cells. N81 fibroblasts also showed partially similar alterations in gene expression to HGPS and WRN cells (white box; Fig. 1A). Compared to N9 fibroblasts, the 285 differentially expressed genes in N81, WRN and HGPS cells (Fig. 1B and Fig. S1A) were clustered into downregulated cell cycle, DNA and histone segregation, and DNA replication genes (Fig. 1C) and upregulated cell adhesion genes (Fig. S1B). This result is consistent with our and others’ previous reports that senescence blocks cell cycle progression and induces cell adhesion^32–35^. We confirmed the alteration of several genes including LMNA, IL-8, CENP-E, and Rad51 by RT-PCR (Fig. S1C). This result is consistent with our and other previous reports that fibroblasts derived from prematurely aged and naturally aged subjects exhibit cellular senescence such as blocked cell cycle progression and induced cell adhesion^32^. Six specific gene sets were altered only in HGPS (Fig. S2A), 24 specific elements only in WRN cells (Fig. S2B), and 51 elements only in N81 fibroblasts (Fig. S2C) compared to N9 fibroblasts. We also analyzed the commonly regulated gene sets in WRN and HGPS cells. Compared to N9 fibroblasts, 881 genes were altered in both HGPS and WRN cells (Fig. 1D and Fig. S2D and S2E) and their functions were categorized into nine elements (Fig. 1E), which were mainly involved in cell cycle progression. We also confirmed by western blot assay that common alterations occurred in HGPS and WRN cells (Fig. 1F). Fluorescence staining of Ki67 showed that the proliferation of HGPS and WRN cells was reduced (Fig. S3A and S3B). Conversely, fluorescence staining of paxillin and phalloidin showed that cell adhesion and cell size were increased in both HGPS and WRN cells compared to N9 fibroblasts (Fig. S3C and S3D). Furthermore, we examined the basal level of γ-H2A.X foci in N9, HGPS, and WRN fibroblasts by immunofluorescence assay. HGPS and WRN fibroblasts showed a slightly higher level of γ-H2A.X expression than N9 fibroblasts (Fig. S3E). Senescence-associated β-galactosidase (SA-β-gal) activity was increased in HGPS and WRN cells (Fig. S3F). We also counted each cell line for 5 days to compare the rate of cell propagation and the doubling time of the population. The cell propagation of N9 fibroblasts was much higher than that of HGPS and WRN cells. The density of N9 fibroblasts doubled within 24 h, while HGPS and WRN cells took more than 3 days to double. In particular, WRN cells took a longer time than HGPS cells to double (Fig. S3G). These results indicate that HGPS and WRN cells (and to a lesser extent N81 cells) have very similar cellular senescence phenotypes.

### Fibroblasts derived from patients with WRN express progerin

**Figure 2:**
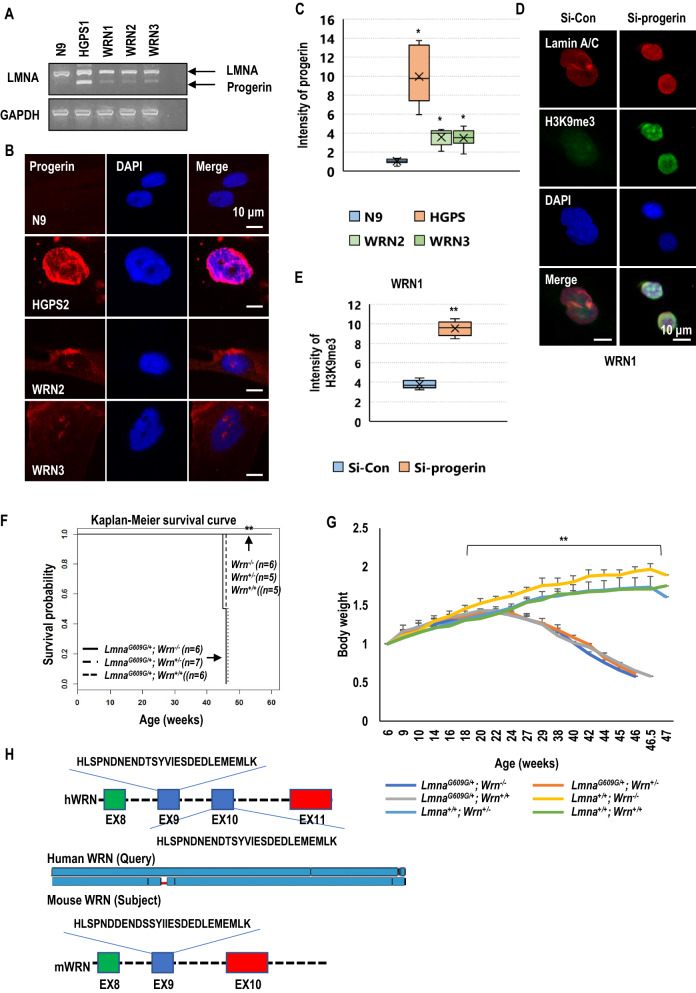
Fibroblasts derived from WRN patients express progerin. (**A**) Analysis of lamin A cDNA in N9, HGPS, and WRN fibroblasts. RNA was extracted from HGPS, WRN and normal control cell lines (at passage 12) and RT-PCR was performed. A large amount of progerin was expressed in HGPS cells. A small amount of progerin was expressed in WRN cells. GAPDH was used as a loading control (*n* = 3 independent experiments; two-tailed Student’s *t*-test). (**B**) Analysis of progerin expression and nuclear aberrations in WRN and HGPS cells (at passage 11) by immunofluorescence (IF) assay. Progerin expression was detected by using an anti-progerin antibody. DAPI was used for nuclei staining. (**C**) The box plot shows the relative expression of progerin in normal fibroblasts, HGPS, and WRN cells (*n* = 3 independent experiments; unpaired *t*-test), Data are the mean ± SD. (**D**) Expression of H3K9me3 was induced after the elimination of progerin in WRN cells (at passage 11) by an siRNA system. Knockdown of progerin induced H3K9me3 and reduced nuclear size in WRN cells. WRN cells were transfected with siRNA-control (Si-Con; nontarget sequence) or siRNA-progerin (Si-progerin) for 48 h and stained with anti-H3K9me3 antibody and DAPI (*n* = 3 independent experiments; unpaired *t*-test). (**E**) The box plot shows the intensity of H3K9me3 expression after transfection with siRNAs. (**F**) The survival rate of *Lmna*^*G609G/*+^ mice was not affected by deletion of the mouse *Wrn* gene. Survival curves were determined by Kaplan–Meier analysis. (**G**) The graph shows the change in body weight of 6 mouse models (*Lmna*^+*/*+^ *Wrn*^+*/*+^*, Lmna*^+*/*+^ *Wrm*^+*/-*^*, Lmna*^+*/*+^ *Wrn*^*−/−*^*, Lmna*^*G609G/*+^ *Wrn*^+*/*+^*, Lmna*^*G609G/*+^ *Wrn*^+*/-*^*,* and *Lmna*^*G609G/*+^ *Wrn*^*−/−*^). There was no difference in body weight between *Lmna*^*G609G/*+^ and *Lmna*^*G609G/*+^; *Wrn*^*−/−*^ mice or between *Wrn*^+*/*+^ and *Wrn*^*−/−*^ mice. (**H**) Amino acid sequence analysis between human WRN (hWRN) and mouse WRN (mWRN). Twenty-eight amino acids are repeated in hWRN but not in mWRN. In hWRN, the sequences of exon 9 are repeated in exon 10. **p < 0.001. Data are the mean ± SD.

Since normal cells of healthy aged people can produce progerin^36^, we examined the expression of progerin in WRN cells. RT-PCR analysis showed a small amount of progerin in WRN cells and N81 fibroblasts (Fig. 2A and Fig. S1C). To determine whether the lower band was progerin, we performed cloning and sequencing of this product and confirmed a perfect match with progerin (Fig. S4A). However, these WRN cells did not possess genetic mutations in the genomic DNA (Fig. S4B and S4C). These results indicate that progerin can be expressed by an alternative splicing process without a HGPS-related mutation in WRN cells. Additionally, we detected progerin expression by IF staining (Fig. 2B) and found that, its level was much lower in WRN cells than in HGPS cells (Fig. 2C). To examine whether the expression of progerin is related to the senescence phenotype of WRN, we eliminated progerin using siRNA and measured the expression of H3K9me3, which is decreased in senescent cells^37–42^. Elimination of progerin obviously induced H3K9me3 expression in WRN cells but did not affect the expression of lamin A (Fig. 2D,E, Fig. S5A and S5C). To confirm that progerin is one of the triggers inducing premature aging in WRN, we crossbred heterozygous *Lmna*^*G609G*^ mice with *Wrn*-deficient mouse to generate a mouse model expressing progerin in *Wrn*-deficient background (Fig. S5D). However, unlike humans, *Wrn*-deficiency alone does not cause premature aging phenotypes in mice^18,21^ and progerin is not naturally expressed in the mouse model^43,44^. Interestingly, heterozygous *Lmna*^*G609G*^ mice did not show any differences in pathophysiological phenotypes including body weight and lifespan with or without the mouse *Wrn* gene (Fig. 2F,G). To explore why the *Wrn*-deficient mouse model does not exhibit premature aging phenotypes, we analyzed and compared the WRN sequences of humans and mice. We found that human WRN possesses repeated regions (Fig. 2H), due to exon duplication (in fact, exon 9 and exon 10 share the same DNA sequence encoding the same amino acid sequence in humans; Fig. S6A and S6B). In contrast, mice do not have consistent duplicated sequences like those in humans. The exon 9 sequence of mouse WRN is not duplicated (Fig. 2F and Fig. S6A). We hypothesized that the duplicated sequence of human WRN has an important role in the aging process. Thus we generated recombinant proteins consisting of unduplicated sequences (WRN-R1) or duplicated sequences (WRN-R2; Fig. S6C). WRN-R1 is a peptide of the exon 9 sequence of human WRN that mimics the unduplicated exon 9 sequence of mouse WRN. WRN-R2 is a peptide that replaces the duplicated exon 9 sequence of human WRN.

### Human WRN rescues the senescence phenotypes in WRN cells

**Figure 3:**
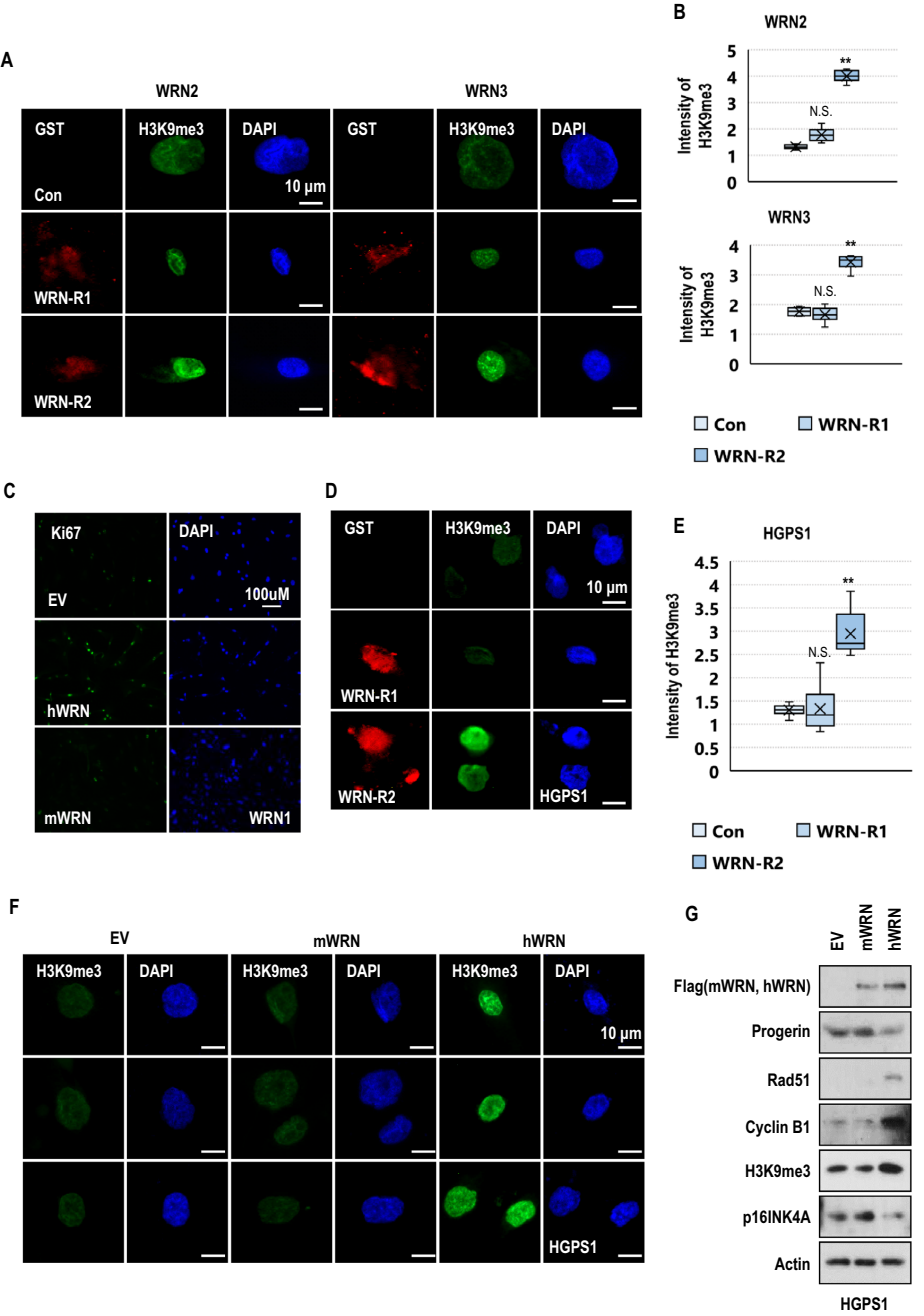
Specific duplicated regions in hWRN are critical for senescence. (**A**) Transfection of GST-tagged WRN-R2 (duplicated peptide) and WRN-R1 (nonduplicated peptide) recombinant proteins into WRN cells (at passage 10) for 24 h. The delivery of WRN-R2 induced H3K9me3 expression in WRN cells compared to WRN-R1 (*n* = 3 independent experiments; unpaired *t*-test). (**B**) The box plots show the intensity of H3K9me3 expression in WRN cells after the delivery of WRN-R1 and WRN-R2. All values are reported as the mean ± SD. (**C**) Transfection of hWRN and mWRN expression vectors into WRN cells (at passage 10). EV refers to empty vector which was used as a negative control. The expression of Ki67 was induced after transfection with hWRN for 48 h (*n* = 3 independent experiments; unpaired *t*-test). (**D**) Transfection of WRN-R1 and WRN-R2 peptides into HGPS cells (at passage 11) for 24 h. The expression of H3K9me3 was increased in WRN-R2-positive cells (*n* = 3 independent experiments; unpaired *t*-test). (**E**) The box plot shows the intensity of H3K9me3 expression in HGPS cells after the delivery of WRN-R1 and WRN-R2 peptides. Data are the mean ± SD. (**F**) Transfection of hWRN and mWRN expression vectors into HGPS cells (at passage 11) for 48 h. Transfection of hWRN, but not mWRN, induced H3K9me3 expression in HGPS cells. (*n* = 3 independent experiments; unpaired *t*-test). (**G**) Overexpression of hWRN reduced the expression of progerin and p16INK4A and induced the expression of rad51, cyclin B1, and H3K9me3 in HGPS cells (at passage 11). Blots were cropped from different parts of the same samples and analyzed by film-based imaging systems. **p < 0.001, N.S: not significant. Data are mean ± SD.

To investigate whether the duplicated region of human WRN affects premature aging, we first delivered the recombinant proteins into WRN cells for 24 h and observed the changing phenotypes. WRN-R2 induced the expression of H3K9me3 and reduced the expression of p16INK4A in WRN cells (Fig. 3A,B, Fig. S7A to S7C). The expression of Ki67 was increased in WRN-R2-positive WRN cells (Fig. S7D and S7E). Although WRN-R1 also partially reduced the expression of p16INK4A (Fig. S7B), it did not differ significantly from the control group in general (Fig. 3A,B, Fig. S7A, S7D, and S7E). Next, we checked the rescue effect of human WRN on premature aging by using DNA vectors. We transferred DNA vectors expressing human WRN (hWRN) and mouse WRN (mWRN) in WRN cells. The overexpression of hWRN, but not mWRN, increased KI67 and H3K9me3 expression (Fig. 3C and Fig. S8A to S8E). Conversely, the average number of 53BP1 foci and the levels of γ-H2A.X foci were decreased after transfection with the human WRN expression vector (Fig. S8F to S8J). These results strongly suggest that only hWRN is involved in the aging process and explain why *Wrn*-deficient mice do not show aging features.

### Human WRN reduces progerin expression and rescues the senescence phenotypes in HGPS cells

**Figure 4:**
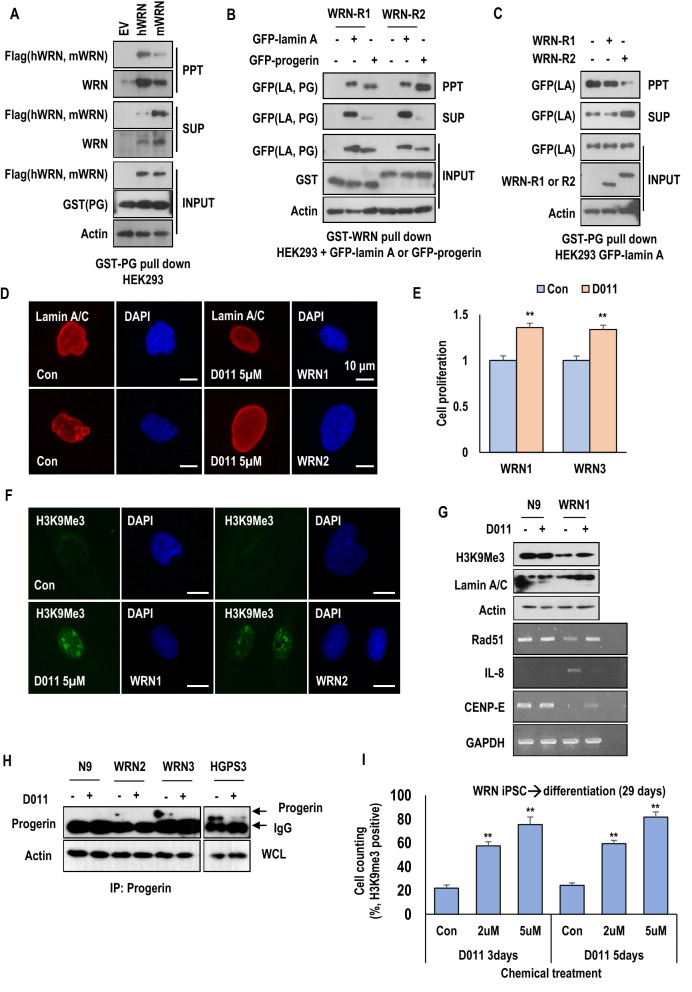
SLC-D011 can ameliorate the premature aging features of WRN cells. (**A**) GST pulldown assay using HEK293 cell lysates expressing hWRN or mWRN. The lysates were incubated with bead-conjugated GST-tagged progerin recombinant proteins. hWRN, but not mWRN, had a strong interaction with progerin. Blots were cropped from different parts of the same samples and analyzed by film-based imaging systems (*n* = 3 independent experiments; two-tailed Student’s *t*-test). (**B**) The duplicated region of human WRN is critical for progerin binding with WRN. Bead-conjugated GST-tagged WRN-R1 and GST-tagged WRN-R2 recombinant proteins were incubated with GFP-lamin A (LA)- or GFP-progerin (PG)-transfected HEK293 cell lysates. After the pull-down assay, GFP-binding proteins were measured by western blot assay. Blots were cropped from different parts of the same samples and analyzed by film-based imaging systems (*n* = 3 independent experiments; two-tailed Student’s *t*-test). (**C**) WRN-R2 blocks the interaction between lamin A and progerin. Bead-conjugated GST-tagged progerin (GST-PG) recombinant proteins were incubated with GFP-lamin A-transfected HEK293 lysates with WRN-R1 or WRN-R2 peptide. Addition of the WRN-R2 peptide but not the WRN-R1 peptide, decreased the interaction between lamin A and progerin. Blots were cropped from different parts of the same samples and analyzed by film-based imaging systems (*n* = 3 independent experiments; two-tailed Student’s *t*-test). (**D**) SLC-D011 ameliorates nuclear abnormality in WRN cells. WRN cells (at passage 11) were incubated with SLC-D011 for 48 h and stained with anti-lamin A/C antibody and DAPI (*n* = 3 independent experiments; two-tailed Student’s *t*-test). (**E**) SLC-D011 improved the cell proliferation of WRN cells. WRN cells (at passage 11) were incubated with SLC-D011 for 7 days and counted by using IF images (*n* = 3 independent experiments; two-tailed Student’s *t*-test). (**F**) SLC-D011 induced H3K9me3 expression in WRN cells. WRN cells (at passage 12) were incubated with SLC-D011 (5 μM) for 48 h and stained with anti-H3K9me3 antibody (*n* = 3 independent experiments; two-tailed Student’s *t*-test). (**G**) The expression of H3K9me3, CENP1, and rad51 was induced and the expression of IL-8 was reduced after treatment with SLC-D011 in WRN cells. N9 fibroblasts and WRN1 cells (at passage 11) were incubated with 5 μM of SLC-D011 for 24 h and analyzed by WB and RT-PCR. Western blots were cropped from different parts of the same samples and analyzed by film-based imaging systems (*n* = 3 independent experiments; two-tailed Student’s *t*-test). (**H**) Reduction in progerin expression by treatment with SLC-D011 (5 μM, 7 days) in WRN cells. All primary fibroblasts were harvested at passage 12. To detect the expression of progerin in WRN cells, we performed an immunoprecipitation assay (IP) with an anti-progerin antibody (Abcam) for 1 day at 4 °C and detected the results using a different anti-progerin antibody (Santa Cruz). We observed a small amount of progerin in WRN cells and detected a reduction in progerin after treatment with SLC-D011, similar to the results in HGPS cells. Blots were cropped from different parts of the same samples and analyzed by film-based imaging systems. (**I**) SLC-D011 can improve the proliferation of cardiomyocytes (CMs) derived from WRN iPSCs. CMs derived from WRN iPSCs were incubated with two different concentrations (2 μM and 5 μM) of SLC-D011 for 3 days and 5 days after 29 days of differentiation. The graph shows the percentage of H3K9me3-positive in CMs derived from WRN iPSCs (*n* = 3 independent experiments; two-tailed Student’s *t*-test), **p < 0.001. Data are mean ± SD.

To investigate the effect of hWRN on progerin expression and premature aging phenotypes, we also conducted experiments in HGPS cells. As seen in WRN cells, WRN-R2 also increased the expression of H3K9me3 and Ki67 in HGPS cells (Fig. 3D,E and Fig. S9A to S9C). However, WRN-R1 had only a slight effect on the expression of Ki67 (Fig. S9C). These results confirmed again that the repeated region of hWRN might be related to the inhibition of aging. Therefore, we next tested the effect of hWRN on HGPS cells by using hWRN and mWRN expression vectors. Overexpression of hWRN, but not mWRN, induced H3K9me3 and Ki67 expression in HGPS cells (Fig. 3F,G and Fig. S9D and S9E). Moreover, hWRN reduced the expression of progerin and p16INK4A and increased the levels of Rad51 and cyclin B1 in HGPS cells (Fig. 3G). The basal levels of DNA damage-related genes, including 53BP1 and γ-H2A.X were reduced in hWRN-positive cells (Fig. S9F to S9J). We also observed that SA-β-gal activity was reduced 48 h after transferring the hWRN expression vector (Fig. S9K). These results suggest that hWRN, especially its duplicated region, is closely related to progerin-induced senescence. To address this, we first tested the interaction between WRN and wild-type (WT) lamin A or progerin. By an immunoprecipitation (IP) assay using an anti-WRN antibody, we observed the interaction of WRN with progerin as well as WT-lamin A. However, the interaction between WRN and progerin seemed to be stronger than the interaction between WRN and WT-lamin A (Fig. S10A). To confirm their interaction, we performed a GST-pulldown assay using recombinant progerin proteins and found that hWRN interacted strongly with progerin (Fig. 4A). WRN-R2 showed a much stronger binding affinity than WRN-R1 with progerin (Fig. 4B). Moreover, WRN-R2 blocked the interaction between lamin A and progerin (Fig. 4C). Considering our previous finding that progerin induces premature aging by abnormal binding with lamin A^32^, this result suggests that hWRN is a natural inhibitor of progerin and a protector against progerin-induced senescence by inhibiting progerin from binding to wild-type lamin A.

### An inhibitor of progerin (SLC-D011) can ameliorate the senescence phenotypes in WRN cells

We predicted that if premature aging syndrome caused by hWRN deficiency is affected by progerin, then a progerin inhibitor could have a favorable effect on WRN cells. To test this hypothesis, we treated WRN fibroblasts with a progerin inhibitor, SLC-D011 (D011 also called progerinin; an optimized chemical version of JH4^32^). WRN cells showed abnormal nuclear morphology (deformed nuclei; Fig. 4D and Fig. S10B). Treatment with D011 normalized the nuclear morphology of WRN cells (Fig. 4D and Fig. S10B), promoted cell proliferation (Fig. 4E), and reduced SA-β-gal activity (Fig. S10C). The expression of H3K9me3 was induced by D011 treatment (Fig. 4F,G and Fig. S10D). We also observed increased of CENP1 and Rad51 expression and decreased IL-8 expression after treatment with D011 at the transcriptional level (Fig. 4G and Fig. S10E). Next, we performed an IP assay to directly measure progerin expression in WRN cells. Although it was extremely difficult to detect progerin expression in WRN cells at the protein level, we observed that treatment with D011 could suppress progerin expression in WRN cells, similar to HGPS cells (Fig. 4H and Fig. S11A). We also confirmed the reduction in progerin expression and consistent induction of H3K9me3 expression after treatment with D011 by IF assay (Fig. S11B to S11D). In fact, cardiovascular disease is one of the symptoms and main causes of death in WRN. Therefore, we tried to explore the anti-senescence effect of D011 in WRN cardiomyocytes. We generated iPSCs from WRN fibroblasts and differentiated them into cardiac muscle cells for 29 days (Fig. S12A to S12D). The expression of H3K9me3 and cyclin B1 in WRN iPSC-derived cardiac muscle cells was induced after treatment with D011 (Fig. 4I and Fig. S12E to S12G). These results strongly suggest that the progerin inhibitor D011 has potential as a treatment for patients with WRN.

## Discussion

In this study, we found that progerin is expressed in fibroblasts derived from patients with Werner syndrome (WRN) without genetic mutation as much as in fibroblasts derived from a person with healthy normal aging (N81) and suggested that progerin could be an influential factor to induce premature senescence in WRN patients. We confirmed that WRN cells have a very similar gene expression profile to HGPS cells and a partially similar profile to fibroblasts from an unaffected aged person (N81, Fig. 1A,B,D). Genes related to the cell cycle, DNA replication, DNA repair, and histone segregation were commonly downregulated in HGPS, WRN, and N81 fibroblasts compared to fibroblasts derived from a normal young person (N9, Fig. 1C), while cell adhesion-related genes were upregulated in senescence models (Fig. S1B). Based on gene ontology, we tested senescence-related markers by western blotting, RT-PCR, and IF assays (Fig. 1F and Fig. S3). The cell proliferation (KI67) and doubling time (cell counts for 5 days) of HGPS and WRN cells were reduced compared with those of N9 fibroblasts. The expression of cell cycle-related genes (cyclin B1 and cdc25c), DNA repair-related genes (BRCA1 and Rad51), and H3K9me3 was also reduced in both HGPS and WRN cells. H3K9me3 is necessary for proper chromosome segregation^45,46^, and the expression of H3K9me3 is well known as a proliferation marker in HGPS studies^37–42^. A WRN stem cell model also showed a reduction in H3K9me3 under WRN-deficient conditions^38^. It seems that the reduction in H3K9me3 in HGPS and WRN cells is related to the downregulation of chromosome segregation in gene ontology analysis (Fig. 1C). Conversely, the cell size and cell adhesion were increased. In addition, the basal expression of γ-H2A.X and p16INK4A was also increased in HGPS and WRN cells compared to N9 fibroblasts. Overall, as seen in our previous study^32^, the characteristics of the cellular senescence phenotype commonly appear in HGPS and WRN cells. Additionally, we observed that a small amount of progerin was expressed in WRN cells and N81 fibroblasts. Normal healthy people can produce progerin and the amount of progerin increases with age^29,30^. We observed that the amount of progerin in WRN cells was approximately the same as in N81 fibroblasts, even though the subjects who provided the WRN cells were in their 20 s and 30 s. We thought that the accumulation of progerin would be faster in WRN-deficient conditions than normal aging conditions. However, when we crossbred transgenic *Lmna*^*G609G*^ progeroid mice with *Wrn*-deficient mice to generate a mouse model expressing progerin under *Wrn*-deficient conditions, the pathological phenotypes and lifespan of *Lmna*^*G609G*^ progeroid mice did not differ depending on the presence or absence of the *Wrn* gene. In fact, *Wrn*-deficient mice have almost same physiological phenotypes and lifespan as wild-type mice^21^. We concluded that the function of mWRN is not related to progerin expression since the mouse does not naturally produce progerin^43,44^. Therefore, we expected sequence differences between hWRN and mWRN. To test this hypothesis, we compared the sequences of WRN genes in humans and mice. In addition to other minor differences, the most distinct difference was hWRN possesses repeated regions (exon 9 and exon 10 encode the same amino acid sequence) but mWRN does not. We hypothesized that duplication in hWRN has a role in the senescence process. To confirm our hypothesis, we generated a recombinant protein consisting of a duplicated sequence (WRN-R2) and also generated an unduplicated sequence (WRN-R1) as a negative control. However, the delivery of recombinant protein into primary fibroblasts could not last more than 24 h because cells easily became contaminated or deteriorated after transfection. Nevertheless, the duplicated peptide, WRN-R2, rescued several cellular senescence phenotypes in WRN cells. This duplicated region was also effective in HGPS cells. However, the unduplicated peptide WRN-R1 had little or no effect on progeroid cells. We also observed that senescence phenotypes were ameliorated after transfection with vectors expressing full-length hWRN and mWRN for 48 h. Overexpression of hWRN induced cell proliferation and H3K9me3 expression but reduced DNA damage in both WRN and HGPS cells. In particular, hWRN, but not mWRN, reduced progerin expression and SA-β-Gal activity in HGPS cells. We concluded that hWRN, especially its duplicated regions, are closely related to progerin-induced senescence and examined the interaction of hWRN and progerin. We found that hWRN bound strongly to progerin and inhibited progerin from binding to wild-type lamin A. It seems that the duplicated region of hWRN is important for this binding and thus affects the aging process. Based on the preceding results, we proposed that hWRN might be a natural inhibitor of progerin, and for patients with WRN, premature aging would be caused by progerin accumulation. Therefore, we thought that inhibiting progerin in WRN cells could suppress aging phenotypes, and confirmed this effect by treatment siRNA and with a progerin-inhibitor (SLC-D011). SLC-D011, also called progerinin (a modified chemical version of JH4^32^), is an inhibitor of the interaction between progerin and lamin A. In our previous study, we discovered that progerin strongly binds to lamin A leading to nuclear deformation and aging processes in HGPS^47^. We observed that progerin was stabilized by interaction with lamin A and developed the binding inhibitor SLC-D011, which inhibited progerin from binding to wild-type lamin A and suppressed the expression of progerin in HGPS. Although WRN protein is also expressed in HGPS, it is thought that the amount of WRN protein is insufficient to prevent progerin accumulation because the level of progerin in HGPS is very high. However, SLC-D011 can substantially decrease progerin levels in HGPS and improve premature aging phenotypes. Likewise, this binding inhibitor of progerin ameliorated the aging features of WRN cells in this study. Furthermore, the main cause of death in WRN is cardiovascular disease or cancer^2,48^. Therefore, we treated cardiomyocytes derived from WRN iPSCs with SLC-D011. The expression of H3K9me3 and cyclin B was also induced in cardiac muscle cells after treatment with SLC-D011.

**Figure 5:**
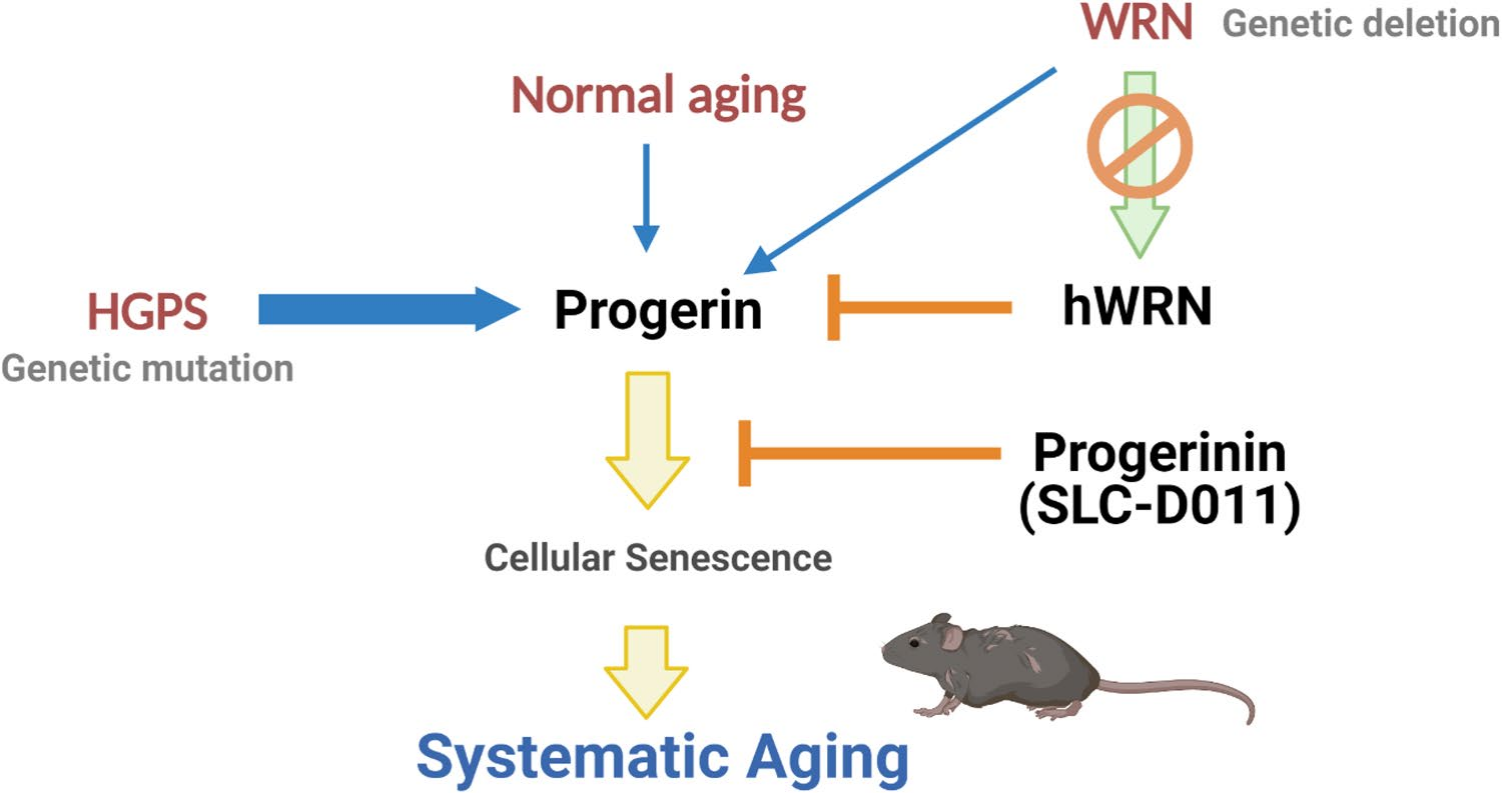
Summary diagram. Progerin can be produced by genetic mutation (HGPS) or the normal aging process. The thickness of the blue arrow indicates the intensity of progerin expression. Human WRN (hWRN) binds to progerin and inhibits progerin expression under normal conditions. Under WRN-deficient conditions, such as in WRN, progerin can accumulate easily and promote cellular senescence. SLC-D011 binds to progerin and eliminates its expression. Thus, SLC-D011 can be used to treat WRN and normal aging as well as HGPS. The diagram was created with BioRender.com.

In this study, we suggest that progerin can be involved in causing premature aging in WRN and that human WRN protein is a natural inhibitor of progerin (Fig. 5). In addition, we revealed that the repeated regions of hWRN are important in this aging process. In contrast, it is assumed that mWRN is not related to this aging process because progerin is not naturally expressed in mice, and there is no duplication in mWRN. Therefore, the *Wrn*-deficient mouse model does not show human-like premature aging phenotypes, and the phenotypes of the *Lmna*^*G609G*^ progeroid mouse model are not affected by the presence or absence of mWRN. Based on these results, we are not sure but expect that expressing the duplicated peptide of WRN in *Lmna*^*G609G*^ progeroid mice will affect the premature senescence phenotypes of the mouse model. Therefore, we are currently in the process of generating the corresponding mouse model, which will be discussed in our next paper. Moreover, SLD-D011 (progerinin) has favorable effects in WRN cells, so this compound could be a plausible drug candidate for patients with WRN in the future.

## Materials and methods

### Animal experiments

All methods performed in this study were approved by the Institutional Review Board (IRB) at Pusan National University (PNU) in accordance with relevant guidelines and regulations. Animal experiments were approved by the Institutional Animal Care and Use Committee (IACUC) at PNU and were performed in a facility certified by the Association for Assessment and Accreditation of Laboratory Animal Care in compliance with animal policies approved by PNU. Progerin-heterozygous *Lmna*^*G609G/*+^ mice were provided by Carlos López-Otín (Universidad de Oviedo, Asturias, Oviedo, Spain). Homozygous *Wrn*^*−/−*^ mice were provided by Michel Lebel (CHU of Quebec Research Center, Québec, Canada). *Wrn*-deficient heterozygous *Lmna*^*G609G/*+^ mice (*Wrn*^*−/−*^; *Lmna*^*G609G/*+^ and *Wrn*^+*/−*^; *Lmna*^*G609G/*+^) were generated by cross-mating heterozygous *Lmna*^*G609G/*+^ mice and homozygous *Wrn*^*−/−*^ mice. For genotyping, genomic DNA was extracted from each mouse model. Genomic DNA samples from the mouse model were amplified by PCR using specific primers. *LmnaG609G*: primer #1, 5′-AAGGGGCTGGGACAGAG-3′; primer #2, 5′-AGTAGATGGCGCGAAGG-3′; primer #3, 5′-AGCATGCAATAGGGTGGAAGGA-3′ and *Wrn*: primer #1, 5′-CAATAACCAATGGAATTCTAAGC-3′; primer #2, 5′-TCAAATTTTATCCCAACCCTTAG-3′; primer #3, 5′-GCCTGCAGCTGGCGCCATC-3’.

### Cell culture and reagents

Primary human fibroblast cells from HGPS patients (AG03198, 10-year-old female, HGPS1; AG11513, 8-year-old female, HGPS2; AG11498, 14-year-old male, HGPS3), WRN patients (AG06300, 37-year-old male, WRN1; AG05229, 25-year-old male, WRN2; AG03141, 30-year-old female, WRN3) and unaffected controls (GM00038, 9-year-old female, N9; AG09603, 81-year-old female, N81) were obtained from Coriell Cell Repositories (Camden, NJ, USA) and maintained in Eagle’s Minimal Essential Medium (EMEM) supplemented with 15% fetal bovine serum (FBS) and 2 mM glutamine or in EMEM with 26 mM HEPES without antibiotics. HEK293 cells were obtained from the American Type Culture Collection (ATCC, Manassas, VA, USA) and maintained in liquid medium (DMEM) containing 10% FBS and 1% penicillin–streptomycin at 37 °C with 5% CO_2_. All cell lines were established in our laboratory under study protocols approved by the PNU IRB, in accordance with relevant guidelines and regulations.

### WRN iPSC line and culture conditions

WRN iPSCs were derived from WRN patient skin fibroblasts (AG05229, 25-year-old male, Coriell Cell Repositories, Camden, NJ, USA). The WRN patient-derived iPSC line was maintained on Matrigel-coated plates (BD Biosciences, San Jose, CA, USA) in Essential 8 Medium (Gibco, Life Technology, Carlsbad, CA, USA). The medium was changed every day.

### Differentiation of human iPSC into cardiomyocytes

The protocol used in this study was approved by the IRB at PNU. Human iPSCs at ~ 90% confluence were differentiated into cardiomyocytes using a chemically defined monolayer differentiation method as described in previous studies^49–51^. Briefly, human iPSCs were incubated in insulin-minus RPMI + B27 supplemented with CHIR99021, a selective glycogen synthase kinase 3β inhibitor, for 48 h. After that, iPSCs were recovered in insulin-minus RPMI-B27 without CHIR99021 for 24 h, treated with the Wnt antagonist IWR-1, for 48 h, treated with fresh insulin-minus RPMI + B27 for another 48 h, and finally switched to RPMI + B27 medium with insulin. Approximately 30 days after cardiac differentiation, iPSC-CM monolayers were subsequently dissociated using TrypLE Express for experimental use.

### Antibodies and reagents

The antibodies used for experiments included antibodies against GFP (1:1000; sc-9996; Santa Cruz Biotechnology, Dallas, TX, USA); GST (1:5000; sc-138; Santa Cruz Biotechnology), actin (1:10,000; sc-47778; Santa Cruz Biotechnology), WRN (1:300; GTX101081; GeneTex and 1:300; sc-376182; Santa Cruz Biotechnology), lamin A/C (1:10,000; sc-376248; Santa Cruz Biotechnology), progerin (1:300; sc-81611; Santa Cruz Biotechnology and ab66587; Abcam, Cambridge, UK), Ki67 (1:400; ab15580; Abcam), H3K9me3 (1:400 and 1:2000; ab8898; Abcam), p16-INK4A (1:300; 10,883–1-AP; Proteintech, Rosemont, IL, USA), cyclin B1 (1:200; sc-594; Santa Cruz), cdc25C (1:500; #4688; Cell Signaling technology) and Rad51 (1:200; #05-530; Millipore).

### Recombinant proteins

To produce recombinant proteins, the recombinant C-terminal region of lamin A (556–664) and the C-terminal region of progerin were produced by cloning 100 amino acids upstream of the termination codon through PCR. The WRN-R1 peptide (hWRN 424–450) and WRN-R2 peptide (hWRN 424–476) were generated by a similar strategy. Each fragment was loaded onto GSH-agarose and then eluted using a buffer containing 20 mM reduced glutathione after extensive washing. These eluted fractions were further purified using anion-exchange chromatography (HiTrap Q HP, GE Healthcare, Chicago, IL, USA). Recombinant proteins were provided by the Department of Food Science, College of Agricultural Science, Seoul Nation University, Seoul.

### Transfection of vectors and siRNA

GFP-fused progerin (GFP-PG) and GFP-fused lamin A (GFP-lamin A) expression vectors were provided by Misteli T. (National Cancer Institute [NCI], Frederick, MD, USA). Flag-tagged hWRN was obtained from Seoul National University. Flag-tagged mouse WRN (mWRN; MR226496) expression vector was obtained from ORIGENE. jetPEI (Polyplus Transfection, New York, USA) and ViaFect transfection reagent (Promega Corporation, Wisconsin, USA) were used for the transfection of these vectors. GFP-PG and GFP-lamin A expression vectors were mixed with 1.5 μl of jetPEI in 150 mM NaCl solution. The mixture was added to cells in 10–15 min. After 4 h of incubation, the medium was replaced with new medium supplemented with 10% FBS. hWRN and mWRN expression vectors were mixed with 2 μl of ViaFect transfection reagent in serum-free medium. For in vitro gene knockdown, siRNA against the target proteins was generated (COSMO GENETECH, Seoul, Korea). The target sequences of the siRNA were as follows: Si-con (5′-AAT TCT CCG AAC GTG TCT CGT TTC AAC CTT ACG AGA CAC GTT CGG AGA ATT-3′), and Si-progerin (5′-GGG TCC ACC CAC CTG GGC TCC TGA GTT CAA GAG ACT CAG GAG CCC AGG TGG GTG GAC CC-3′). Transfection was performed for 48 h using INTERFERin reagent (Polyplus Transfection, New York, USA) according to the manufacturer’s protocol.

### Immunoblotting

Immunoblotting assays were designed under protocols approved by the PNU IRB in accordance with relevant guidelines. Radioimmunoprecipitation assay (RIPA) buffer (50 mM Tris–Cl, pH 7.5, 150 mM NaCl, 1% NP-40, 0.1% SDS, and 10% sodium deoxycholate) was used for protein extraction from cells. After heat-inactivation in sample buffer, proteins were subjected to sodium dodecyl sulfate–polyacrylamide gel electrophoresis (SDS-PAGE) and transferred to polyvinylidene difluoride (PVDF) membranes. Blotted membranes were blocked with 3% skim milk for 1 h and incubated overnight with specific primary antibodies, followed by incubation with horseradish peroxidase-conjugated goat anti-mouse, goat anti-rabbit, or mouse anti-goat IgG secondary antibodies (Pierce, Thermo Fisher Scientific, Inc., Rockford, IL, USA). Signals were detected by chemiluminescence using ECL kit (Intron, Seoul, Korea) following the manufacturer’s instructions. This immunoblotting protocol was used as described in our previous publication^52^.

### Protein–protein interaction analyses

For the analysis of protein–protein interactions, glutathione S-transferase (GST) pull-down assays and IP experiments were performed under protocols approved by the PNU IRB. To detect the interaction, the GST-bead-fused lamin A-C-terminal region, progerin-C-terminal region, WRN-R1 peptide, or WRN-R2 peptide was incubated with GFP-tagged progerin (GFP-progerin) and lamin A (GFP-lamin A) transfected HEK293 cell lysate for 1 h at room temperature (RT). After washing once with PBS, precipitated materials were collected and subjected to SDS-PAGE and western blot analysis with anti-GFP and GST. For the competition assay of WRN-R1 and WRN-R2 against progerin and lamin A binding, bead-conjugated GST-protein was incubated with GFP-lamin A overexpressing HEK293 lysate with or without WRN-R1 or WRN-R2 recombinant protein. For endogenous IP assays, whole-cell lysates were incubated with anti-WRN antibody at 4 °C for 2 h followed by incubation with protein A/G agarose beads at 4 °C for 1 h. In the case of the progerin IP assay in WRN and HGPS cells, whole-cell lysates were incubated with anti-progerin antibody at 4 °C overnight. After centrifugation and washing with RIPA buffer, these immunocomplexes were separated by SDS-PAGE and subjected to western blotting.

### Immunofluorescence staining and senescence-specific acidic β-galactosidase activity staining

The staining assays were performed under protocols approved by the PNU IRB, in accordance with relevant guidelines and regulations. Cells were fixed with 1% paraformaldehyde (PFA) for 1 h at RT, and then permeabilized with 0.2% Triton X-100 at RT for 5 min. Cells were incubated with blocking solution (goat serum diluted 1:400 in PBS) for 1 h, followed by incubation with anti-lamin A/C (1:400), anti-progerin (1:100), anti-flag (1–100), anti-Ki67 (1:200), anti-γ-H2A.X (1:200), anti-53BP1 (1:200), or anti-H3K9me3 (1:200) overnight at 4 °C. Then, the cells were incubated with fluorescein isothiocyanate (FITC) and/or rhodamine-conjugated secondary antibodies at 4 °C for 7 h and stained with DAPI (4, 6-diamidino-2-phenylindole) at RT for 10 min. Paxillin and phalloidin were stained by DAPI. After the cells were washed with PBS, coverslips were mounted with mounting solution (H-5501; Vector Laboratories (Burlingame, CA, USA). Immunofluorescence signals were detected using fluorescence microscopes (ZEIZZ, Germany and Logos Biosystems, Korea). This immunofluorescence protocol was used as described in our previous pubication^52^. For senescence specific acidic-β-galactosidase activity staining, cells were fixed with 0.5% glutaraldehyde for 15 min and stained with X-gal solution overnight at 37 °C as described in the instructions of the Senescence β-Galactosidase Staining Kit (9860; Cell Signaling Technology). The images were analyzed and quantified using NIH ImageJ software (version 1.52a, imagej.nih.gov/ij/).

### Gene expression

For RT-PCR, total cellular RNA was extracted using an RNA extraction kit (QIAGEN). Gene expression studies were performed using cDNA synthesized from total RNA with MMLV RT (Invitrogen, Carlsbad, USA) and random hexamers. PCR from genomic DNA was performed using DiaStar Taq DNA polymerase (SolGent, Daejeon, Korea). Gene expression studies were performed with the following specific primers: Rad51, 5′-CTTTGGCCCACAACCCATTTC-3′ and 5′-ATGGCCTTTCCTTCACCTCCA-3′; IL-8, 5′-TTGGCAGCCTTCCTGATT-3′ and 5′-AACTTCTCCACAACCCTCTG-3′; CENP-E, 5′-AGCTGCTTAGAGAAAAGGAAGACC-3′ and 5′-GCAAAATGACTTCTTCCCGCA-3′; LMNA, 5′-AAGGAGATGACCTGCTCCATC-3′ and 5′-TTTCTTTGGCTTCAAGCCCCC-3′; GAPDH 5′-ATCTTCCAGGAGCGAGATCCC-3′ and 5′-AGTGAGCTTCCCGTTCAGCTC-3′; and LMNA exon 11, 5′-TGGTCAGTCCCAGACTCGCC-3′ and 5′-CGCCTGCAGGATTTGGAGA-3’.

### Sequencing analysis

Genomic DNA or cDNA was extracted from N9, N81, HGPS, and WRN fibroblasts. The PCR products of genomic DNA and cDNA were analyzed using a genetic analyzer instrument (Applied Biosystems, Thermo Fisher Scientific), and the data were processed with Sequencing Analysis Software. Data analysis was performed by COSMO GENETECH (Seoul, Korea).

### Protein delivery

Protein delivery was performed using PULSin (Polyplus Transfection, New York, USA) following the manufacturer’s protocol. To deliver GST-tagged WRN-R1 and WRN-R2 recombinant proteins into HGPS cells, we used PULSin (Polyplus Transfection, New York, USA) following the manufacturer’s protocol. Recombinant protein (2 μg) was diluted with 200 μl of 20 mM HEPES. After dilution, PULSin reagent (8 μl) was added. The mixture was incubated at RT for 15 min. After incubation, the mixture was added to cells. After 4 h of incubation, culture medium was removed from the wells and replaced with fresh serum-containing medium.

### Cell counting

For cell counting, fixed cells were counted in randomly selected fields and expressed as percentages or actual numbers of total cells counted. Cell propagation was analyzed by counting cells daily for 5 days. Cell counting was performed by three independent observers who were blinded to the chemical treatment group. To analyze the intensity of proteins, images were quantified through the “color histogram” function of NIH ImageJ software (version 1.52a, https://imagej.net). Background signals were subtracted from the fluorescence intensities.

### Microarray analysis

Total RNA (500 ng) was extracted using an RNAeasy kit (QIAGEN). RNA labeling, hybridization on Human Gene 1.0 ST arrays (Affymetrix) and data analysis were performed by DNA Link (Seoul, Korea). Genes showing at least twofold differences in either cell line were selected for further analysis.

### Protein network analysis

For protein network analysis, we collected 100 genes from the HARG/JR set and ran a network program on a web application platform (cBioPortal, http://cbioportal.org). The biological process- and cellular component-associated genes in each cell line were analyzed using a public web server (g:Profiler, biit.cs.ut.ee/gprofiler/gost).

### Statistical analysis

Data were analyzed with an unpaired or paired Student’s *t*-test. A P-value < 0.05 was considered significant. Error bars indicate standard deviation (SD). Data for all figures are expressed as the mean ± SD of at least two independent experiments.

## Supplementary Information

Supplementary Information.
