# Author Correction: Human WRN is an intrinsic inhibitor of progerin, abnormal splicing product of lamin A

**DOI:** 10.1038/s41598-021-97463-5

**Published:** 2021-09-01

**Authors:** So‑mi Kang, Min‑Ho Yoon, Su‑Jin Lee, Jinsook Ahn, Sang Ah Yi, Ki Hong Nam, Soyoung Park, Tae‑Gyun Woo, Jung‑Hyun Cho, Jaecheol Lee, Nam‑Chul Ha, Bum‑Joon Park

**Affiliations:** 1 Department of Molecular Biology, Pusan National University, Busan, Republic of Korea; 2 School of Pharmacy, Sungkyunkwan University, Suwon, Kyunggi‑Do Republic of Korea; 3 Program in Food Science and Biotechnology, College of Agriculture and Life Sciences, Seoul National University, Seoul, Republic of Korea

Correction to: *Scientific Reports* 10.1038/s41598-021-88325-1, published online 27 April 2021

The original version of this Article contained an error in Figure 2G, where the legends for mouse models “Lmna^+/+^; Wrn^+/-^” and “Lmna^G609/+^; Wrn^+/+^” were interchanged.

“Lmna^+/+^; Wrn^+/-^

Lmna^G609/+^; Wrn^+/+^”

now reads:

“Lmna^G609/+^; Wrn^+/+^

Lmna^+/+^; Wrn^+/-^”

In addition, the Supplementary Information file published with this Article contained an error in Figure S4B, where the x-axis

“Genomic DNA”

now reads:

“cDNA gDNA”

Finally, in the legend of Figure S4,

“Analysis of lamin A genomic DNA in HGPS and WRN cells (at passage 12). An PCR screening using a specific primer to detect splice sites found in exon 11 of *LMNA*. A fragment under 300 bp in length was found in HGPS cells but not in WRN cells (*n* = 3 independent experiments; two-tailed Student’s *t*-test).”

now reads:

“Analysis of lamin A genomic DNA (gDNA) in WRN cells (at passage 12). An intronic primer was designed for the PCR amplification of the *LMNA* exon 11 in WRN cells. The PCR product of HGPS cells was generated by amplification of cDNA using exon-specific primers (*n* = 3 independent experiments; two-tailed Student’s *t*-test).”

The original Figure 2 with accompanying legend and the Supplementary information file are provided below. The original Article and accompanying Supplementary Information file have been corrected.

**Figure 2:**
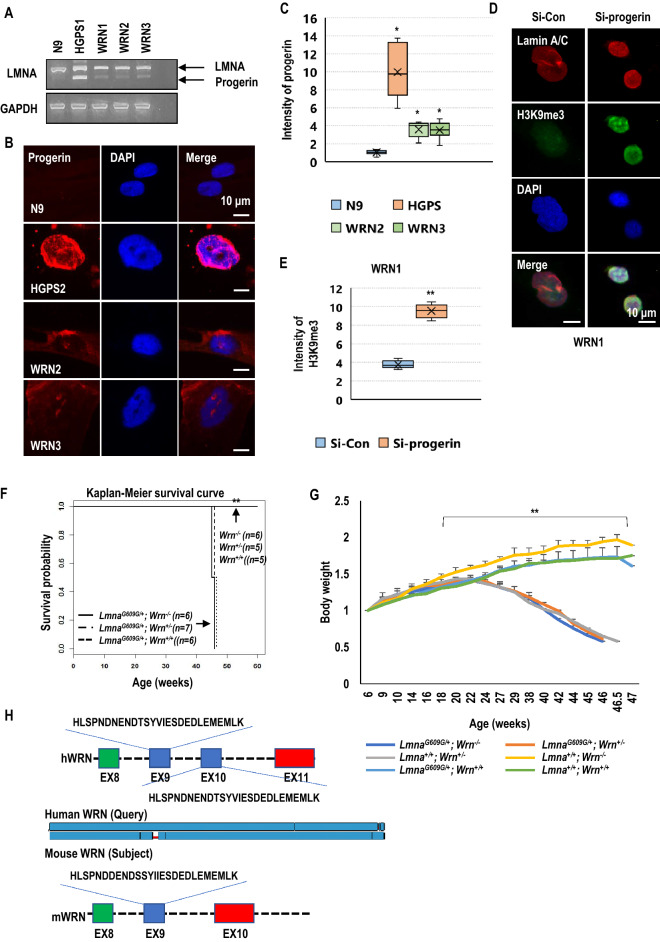
Fibroblasts derived from WRN patients express progerin. (**A**) Analysis of lamin A cDNA in N9, HGPS, and WRN fibroblasts. RNA was extracted from HGPS, WRN and normal control cell lines (at passage 12) and RT-PCR was performed. A large amount of progerin was expressed in HGPS cells. A small amount of progerin was expressed in WRN cells. GAPDH was used as a loading control (*n* = 3 independent experiments; two-tailed Student’s *t*-test). (**B**) Analysis of progerin expression and nuclear aberrations in WRN and HGPS cells (at passage 11) by immunofluorescence (IF) assay. Progerin expression was detected by using an anti-progerin antibody. DAPI was used for nuclei staining. (**C**) The box plot shows the relative expression of progerin in normal fibroblasts, HGPS, and WRN cells (*n* = 3 independent experiments; unpaired *t*-test), Data are the mean ± SD. (**D**) Expression of H3K9me3 was induced after the elimination of progerin in WRN cells (at passage 11) by an siRNA system. Knockdown of progerin induced H3K9me3 and reduced nuclear size in WRN cells. WRN cells were transfected with siRNA-control (Si-Con; nontarget sequence) or siRNA-progerin (Si-progerin) for 48 h and stained with anti-H3K9me3 antibody and DAPI (*n* = 3 independent experiments; unpaired *t*-test). (**E**) The box plot shows the intensity of H3K9me3 expression after transfection with siRNAs. (**F**) The survival rate of *Lmna*^*G609G/*+^ mice was not affected by deletion of the mouse *Wrn* gene. Survival curves were determined by Kaplan–Meier analysis. (**G**) The graph shows the change in body weight of 6 mouse models (*Lmna*^+*/*+^ *Wrn*^+*/*+^*, Lmna*^+*/*+^ *Wrm*^+*/-*^*, Lmna*^+*/*+^ *Wrn*^*−/−*^*, Lmna*^*G609G/*+^ *Wrn*^+*/*+^*, Lmna*^*G609G/*+^ *Wrn*^+*/-*^*,* and *Lmna*^*G609G/*+^ *Wrn*^*−/−*^). There was no difference in body weight between *Lmna*^*G609G/*+^ and *Lmna*^*G609G/*+^; *Wrn*^*−/−*^ mice or between *Wrn*^+*/*+^ and *Wrn*^*−/−*^ mice. (**H**) Amino acid sequence analysis between human WRN (hWRN) and mouse WRN (mWRN). Twenty-eight amino acids are repeated in hWRN but not in mWRN. In hWRN, the sequences of exon 9 are repeated in exon 10. **p < 0.001. Data are the mean ± SD.

## Supplementary Information

Supplementary Information.

