# Supplementary material for: Human WRN is an intrinsic inhibitor of progerin, abnormal splicing product of lamin A
Source: Sci Rep. 2021 Apr 27;11:9122. doi: 10.1038/s41598-021-88325-1 (PMC8079706; doi:10.1038/s41598-021-88325-1)

Human WRN is an intrinsic inhibitor of progerin, abnormal splicing product of lamin A

So-mi Kang [1], Min-Ho Yoon [1], Su-Jin Lee [1], Jinsook Ahn[3], Sang Ah Yi [2], Ki Hong Nam [2], Soyoung Park [1], Tae-Gyun Woo [1], Jung-Hyun Cho[1], Jaecheol Lee [2], Nam-Chul Ha[3], Bum-Joon Park [1, #]

[1]Department of Molecular Biology, Pusan National University, Busan, Korea (Republic of)

[2] School of Pharmacy, Sungkyunkwan University, Suwon, Kyunggi-Do, Korea (Republic of)

[3]Program in Food Science and Biotechnology, College of Agriculture and Life Sciences, Seoul National University, Seoul, Korea (Republic of)

[#]Corresponding author; BJP (bjpark1219@pusan.ac.kr)

Content:

Supplementary Figures:

Supplementary Figure S1: Gene ontology analysis of HGPS, WRN, and N81 fibroblasts compared to N9 fibroblasts.

Supplementary Figure S2: Gene set analysis in HGPS, WRN, and N81 fibroblasts.

Supplementary Figure S3: Similar senescence features in HGPS and WRN fibroblasts.

Supplementary Figure S4: Expression of progerin in WRN cells at the transcriptional level.

Supplementary Figure S5: Progerin is an influential factors in WRN.

Supplementary Figure S6: hWRN has a unique repeated sequence, which is different from mouse WRN.

Supplementary Figure S7: WRN-R2 rescues aging-related markers in WRN fibroblasts.

Supplementary Figure S8: Human WRN induces cell proliferation and ameliorates senescence in WRN fibroblasts.

Supplementary Figure S9: Human WRN ameliorates aging features in HGPS fibroblasts.

Supplementary Figure S10: SLC-D011 can ameliorate the premature aging features of WRN cells.

Supplementary Figure S11: SLC-D011 ameliorates aging features of WRN cells.

Supplementary Figure S12: SLC-D011 induces H3K9me3 expression in cardiomyocytes derived from WRN iPSCs.

Supplementary Figure S13: Uncropped blots

Figure S1

A

285 common elements in HGPS, WRN and N81											
POSTN	SERPING1	GTSE1	HIST1H2BF	CEP78	SYPL2	CENPK	HIST2H3A	OR7E26P	CDC20	DIRC1	TENM2
SNORD115-32	MICA	RFC2	KIF4A	SPAG5	MCM7	LOC100128816	XRCC2	RFC4	GINS1	SELENBP1	GPC6
SNORD28	PLA2G16	GINS3	PRR11	HERC6	MAP2K6	BLM	NCAPG	C1QTNF9B-AS1	KIF20A	IL17RB	SYT14
SNORD27	TTC12	KIF18B	PRC1	CLN6	TRGV5	HIST2H2AB	CENPE	ZNF724P	DDIAS	CDKN3	TFAP2A
SNORD115-44	RPL10	ARHGAP11A	ZNF257	PRIM1	KIF11	ADGRL4	CDK1	CHEK1	TTK	CLGN	APOD
SNORD63	TNXB	HIST1H1B	C1orf112	STMN1	GMNN	SPDL1	SNRNP25	ZNF730	KIAA0101	RASSF2	TNFSF10
SRGN	MIR32	TICRR	ATAD2	BRCA1	BMPR1B	CKAP2L	DTL	PKIA	NETO2	FAM111B	ADH1B
CDH6	PAQR5	CDC45	CDK11A	PIK3R3	ORC1	CENPN	OIP5	TLR3	KCNJ8	BAALC	VIT
MEST	SMC4	CDCA5	NUF2	FAM83D	TRIP13	RAD51AP1	SGOL1	AURKA	GINS2	APOBEC3A_B	BDKRB1
LOXL3	C19orf48	KIF20B	DRP2	FAM64A	TOP2A	SSX2IP	SELPLG	CDC6	POLE2	CCNB2	TFPI2
AFF3	PGM2	PMEPA1	MCM10	EXO5	MASTL	UNG	DLGAP5	MND1	ALPL	OMD	
JAM2	HIST1H3I	CDC25C	RACGAP1	HIST1H3A	PARPBP	MTFR2	HIST1H2BB	ABCA9	ENOX1	SMPDL3A	
TLR4	C17orf53	ADH1A	TCF21	STIL	GDF5	KIAA1524	NUSAP1	CKS2	SKA1	CCNE2	
LRRFIP1	RRM2	SLF1	FANCG	MCM4	PCDHB14	DCLK1	NDC80	SGOL2	CENPU	HTR1F	
LIMD1-AS1	NTSR1	ESCO2	HIST1H2BH	LOC101927806	GSTT2	EZH2	KIF15	ASF1B	RGCC	CRABP2	
TNFSF4	KIFC1	TUBA3FP	RASD2	MELK	MLLT11	GINS4	BUB1	HOXC11	ASPM	VAT1L	
LPCAT2	PITX1	KIF2C	SPC24	CEP55	PQLC2L	WDR76	MMS22L	FANCB	PBK	ADH1C	
TPT1	HJURP	RAB15	RAD54B	FANCI	FBXO5	CKAP2	PAK6	CASC5	MRAP2	BDKRB2	
MMP16	EXO1	TYMS	ZNF367	PLK1	CENPF	DEPDC1	HMMR	RFC3	KIF18A	EDNRB	
SEL1L3	MCM3	ANLN	CDCA7	GEN1	DONSON	OR7E109P	DSN1	NEK2	LY75-CD302	CLCA2	
ADGRL2	TOPBP1	CHAF1B	ATAD5	SHCBP1	BORA	SPC25	CENPQ	CCNB1	APCDD1	STAMBPL1	
RNVU1-18	DTNA	GHR	LOC339803	NCAPH	CHAC2	C4orf33	DAPK1	MMP10	SKA3	CDH10	
HHIP	WDHD1	DCLRE1B	DNA2	CENPI	BRIP1	AR	MAD2L1	CCNA2	EDNRA	ETV1	
CRELD1	OR7E37P	CDCA3	CENPV	PLK4	CDCA2	CDCA8	OR7E12P	HIST1H2BM	HIST1H3E	DPT	
SNORA24	SEPP1	RAD51	HYLS1	SMC2	KIF14	GLMN	SERTAD4	UHRF1	FLRT3	IL33	

B

Common up regulated pathway in HGPS&WRN&N81

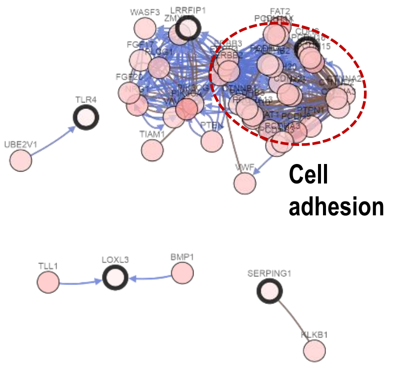

C

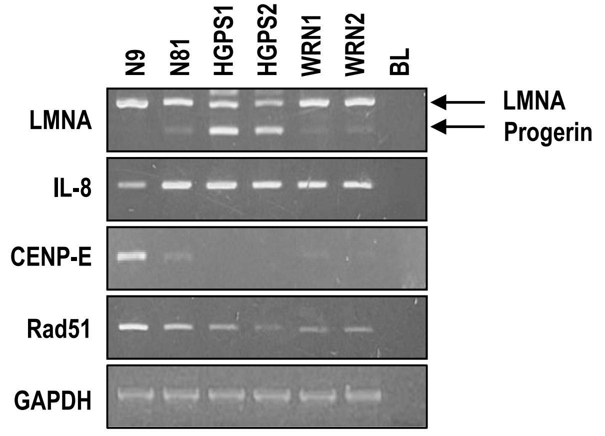

Fig. S1. Gene ontology analysis of HGPS, WRN, and N81 (derived from an unaffected 81-year-old subject) fibroblasts compared to N9 (derived from an unaffected 9-year-old subject) fibroblasts. All fibroblasts were analyzed at passage 10. **A.** Commonly altered genes in HGPS, WRN, and N81 fibroblasts compared to N9 fibroblasts. Large portions of these genes are involved in DNA replication, chromosome separation, DNA repair, and the cell cycle. **B**. Upregulated genes in the core gene set of HGPS, WRN, and N81 cells compared to N9 fibroblasts are associated with cell adhesion. Gene clustering was performed using a gene interaction mapping tool (cBioPortal, http://cbioportal.org). **C.** Analysis of lamin A, IL-8, CENP-E, and Rad51 cDNA in N9, N81, HGPS, and WRN fibroblasts. RNA was extracted from N9, N81, HGPS, and WRN fibroblasts (at passage 10) and RT-PCR was performed.

Figure S2

A

6 elements included exclusively in “HGPS”
extracellular matrix
proteinaceous extracellular matrix
extracellular exosome
cell adhesion
positive regulation of osteoblast differentiation
mitotic cytokinesis

B

24 elements included exclusively in “WRN”
nucleoplasm
sister chromatid cohesion
chromosome, centromeric region
condensed chromosome kinetochore
mitotic sister chromatid segregation
cytosol
CENP-A containing nucleosome assembly
nuclear chromosome
spindle pole
kinetochore
cytoplasm
DNA replication-dependent nucleosome assembly
Cell cycle
regulation of gene silencing
mitochondrial respiratory chain complex I assembly
DNA synthesis involved in DNA repair
mitochondrial inner membrane
nucleolus
NADH dehydrogenase (ubiquinone) activity
nucleus
positive regulation of gene expression, epigenetic
nucleosomal DNA binding
DNA repair
nucleosome

C

51 elements included exclusively in "N81"
nucleosome assembly
Systemic lupus erythematosus
nuclear chromosome, telomeric region
telomere organization
chromatin silencing at rDNA
protein heterotetramerization
protein heterodimerization activity
negative regulation of gene expression, epigenetic
Alcoholism
nuclear nucleosome
DNA replication
histone binding
CDK Regulation of DNA Replication
extracellular region
extracellular space
telomere maintenance via recombination
cellular protein metabolic process
single-stranded DNA-dependent ATPase activity
spindle midzone
positive regulation of inflammatory response
DNA strand elongation involved in DNA replication
metaphase plate congression
MCM complex
regulation of transcription involved in G1/S transition of mit otic cell cycle
DNA helicase activity
spindle microtubule
kinesin complex
G2/M transition of mitotic cell cycle
cell proliferation
spindle
microtubule binding
interstrand cross-link repair
spindle organization
Fanconi anemia pathway
male gonad development
nuclear chromatin
regulation of cell cycle
midbody
Viral carcinogenesis
mitotic metaphase plate congression
negative regulation of megakaryocyte differentiation
Pathways in cancer
response to virus
microtubule-based movement
gene silencing by RNA
DNA replication-independent nucleosome assembly
ATP binding
response to drug
microtubule motor activity
mitotic chromosome condensation
integral component of plasma membrane

D

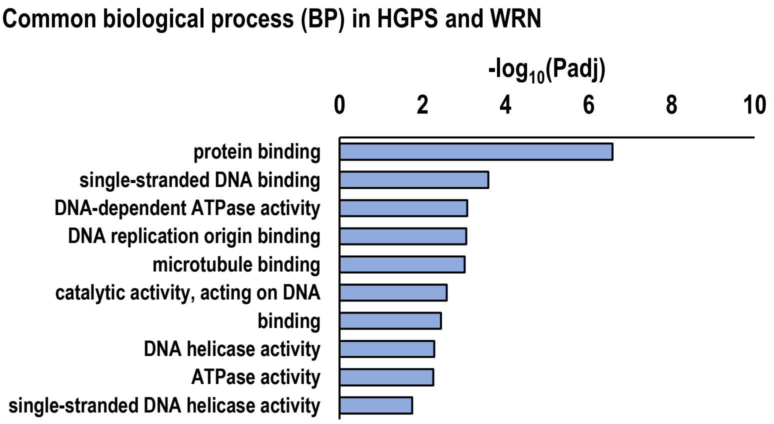

Common biological process (BP) in HGPS and WRN
-log10(Padj)
0
2
4
6
8
10
protein binding
single-stranded DNA binding
DNA-dependent ATPase activity
DNA replication origin binding
microtubule binding
catalytic activity, acting on DNA
binding
DNA helicase activity
ATPase activity
single-stranded DNA helicase activity

E

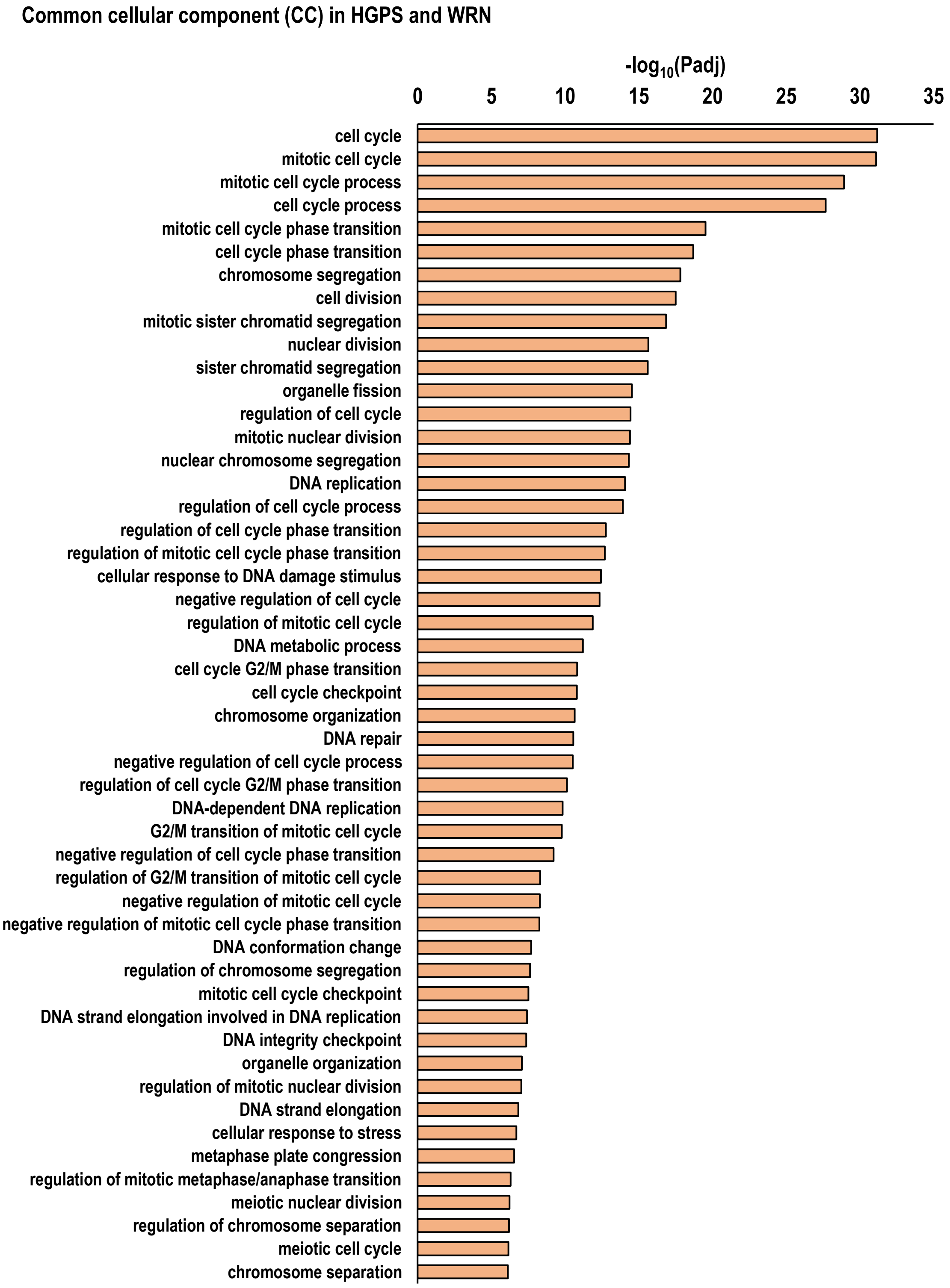

Common cellular component (CC) in HGPS and WRN
-log10(Padj)
0
5
10
15
20
25
30
35
cell cycle
mitotic cell cycle
mitotic cell cycle process
cell cycle process
mitotic cell cycle phase transition
cell cycle phase transition
chromosome segregation
cell division
mitotic sister chromatid segregation
nuclear division
sister chromatid segregation
organelle fission
regulation of cell cycle
mitotic nuclear division
nuclear chromosome segregation
DNA replication
regulation of cell cycle process
regulation of cell cycle phase transition
regulation of mitotic cell cycle phase transition
cellular response to DNA damage stimulus
negative regulation of cell cycle
regulation of mitotic cell cycle
DNA metabolic process
cell cycle G2/M phase transition
cell cycle checkpoint
chromosome organization
DNA repair
negative regulation of cell cycle process
regulation of cell cycle G2/M phase transition
DNA-dependent DNA replication
G2/M transition of mitotic cell cycle
negative regulation of cell cycle phase transition
regulation of G2/M transition of mitotic cell cycle
negative regulation of mitotic cell cycle
negative regulation of mitotic cell cycle phase transition
DNA conformation change
regulation of chromosome segregation
mitotic cell cycle checkpoint
DNA strand elongation involved in DNA replication
DNA integrity checkpoint
organelle organization
regulation of mitotic nuclear division
DNA strand elongation
cellular response to stress
metaphase plate congression
regulation of mitotic metaphase/anaphase transition
meiotic nuclear division
regulation of chromosome separation
meiotic cell cycle
chromosome separation

Fig. S2. Gene set analysis in HGPS, WRN, and N81 fibroblasts. **A.** Six elements exclusively induced in HGPS cells compared to N9 fibroblasts. **B**. Twenty-four elements exclusively induced in WRN cells compared to N9 fibroblasts. **C.** Fifty-one elements exclusively induced in N81 fibroblasts compared to N9 fibroblasts. **D.** Commonly altered biological process (BP)-associated genes in HGPS and WRN cells compared to N9 fibroblasts. Data were analyzed using a public web server for functional enrichment analysis (g:Profiler, biit.cs.ut.ee/gprofiler/gost). **E.** Commonly altered cellular components (CCs) in HGPS and WRN cells compared to N9 fibroblasts. Data were analyzed using the g:Profiler toolsets.

Figure S3

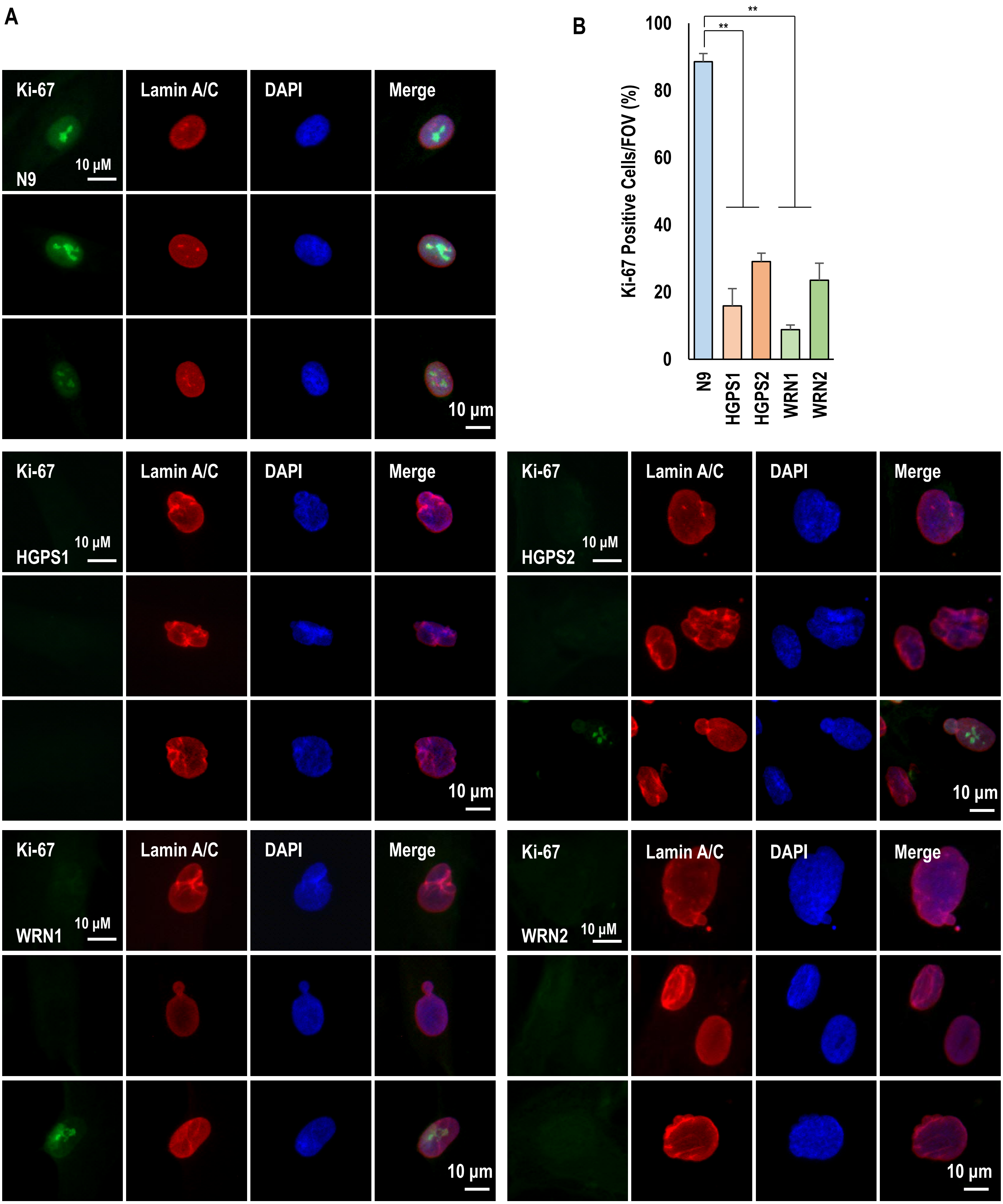
A
B
Ki-67
Lamin A/C
DAPI
Merge
N9
10 μM
10 μm
HGPS1
HGPS2
WRN1
WRN2
Ki-67 Positive Cells/FOV (%)
100
80
60
40
20
0
**
**

C

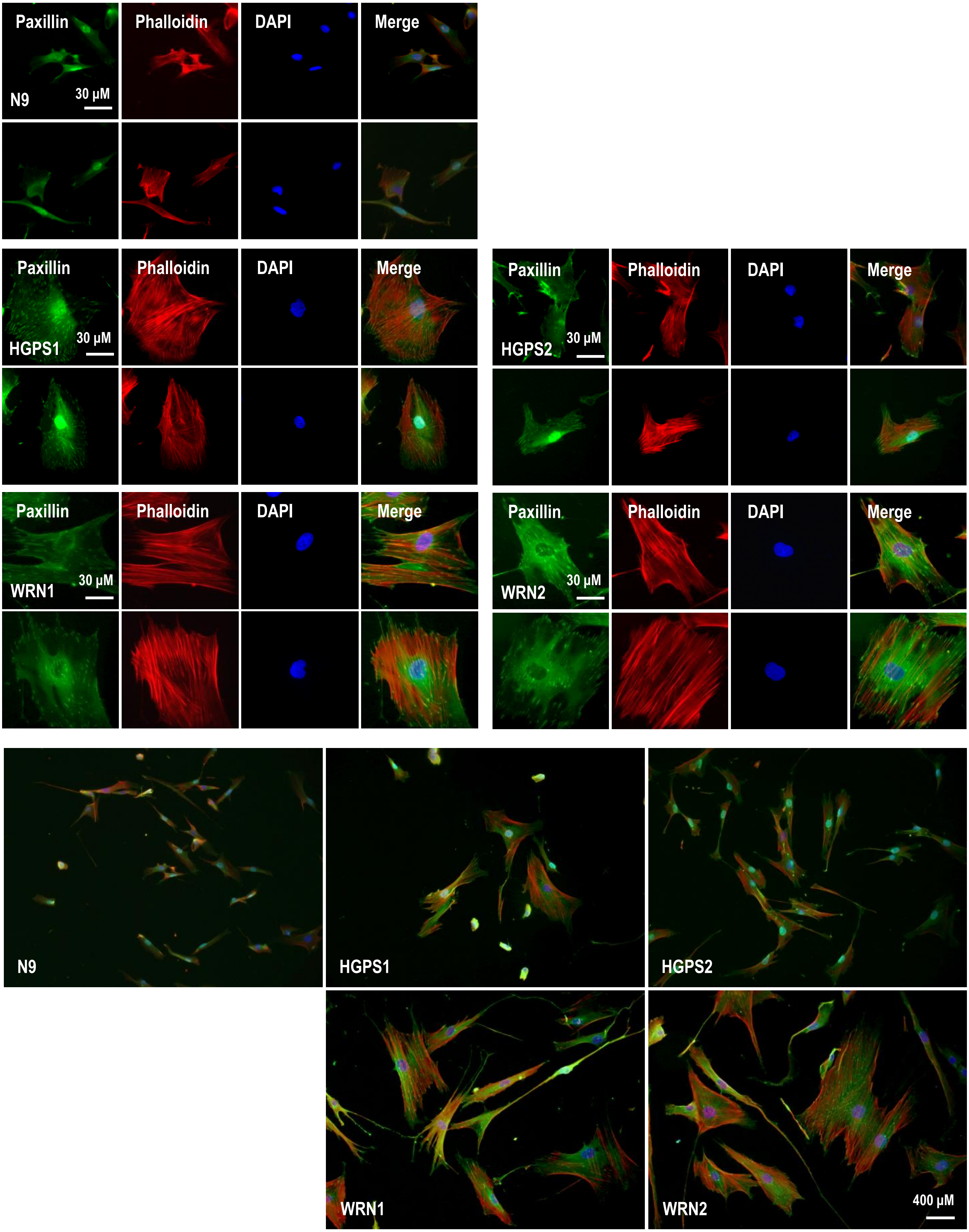
Paxillin
Phalloidin
DAPI
Merge
N9
30 μM
HGPS1
HGPS2
WRN1
WRN2
400 μM

D

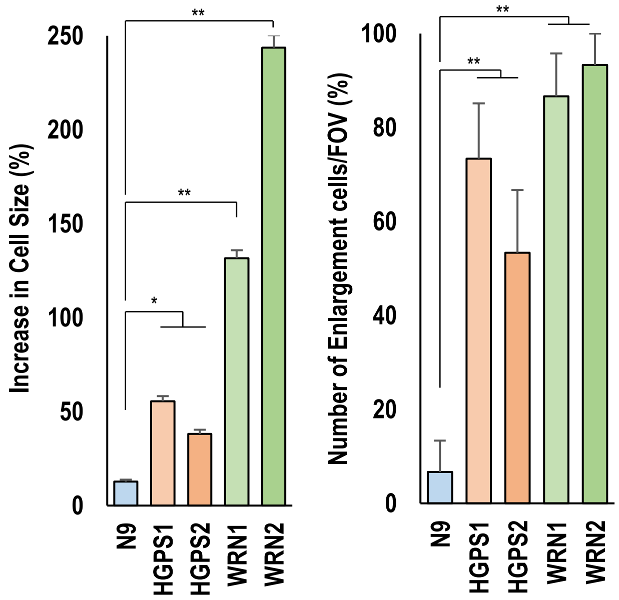

Increase in Cell Size (%)
250
200
150
100
50
0
**
**
*
N9
HGPS1
HGPS2
WRN1
WRN2
Number of Enlargement cells/FOV (%)
100
80
60
40
20
0
**
**
N9
HGPS1
HGPS2
WRN1
WRN2

E

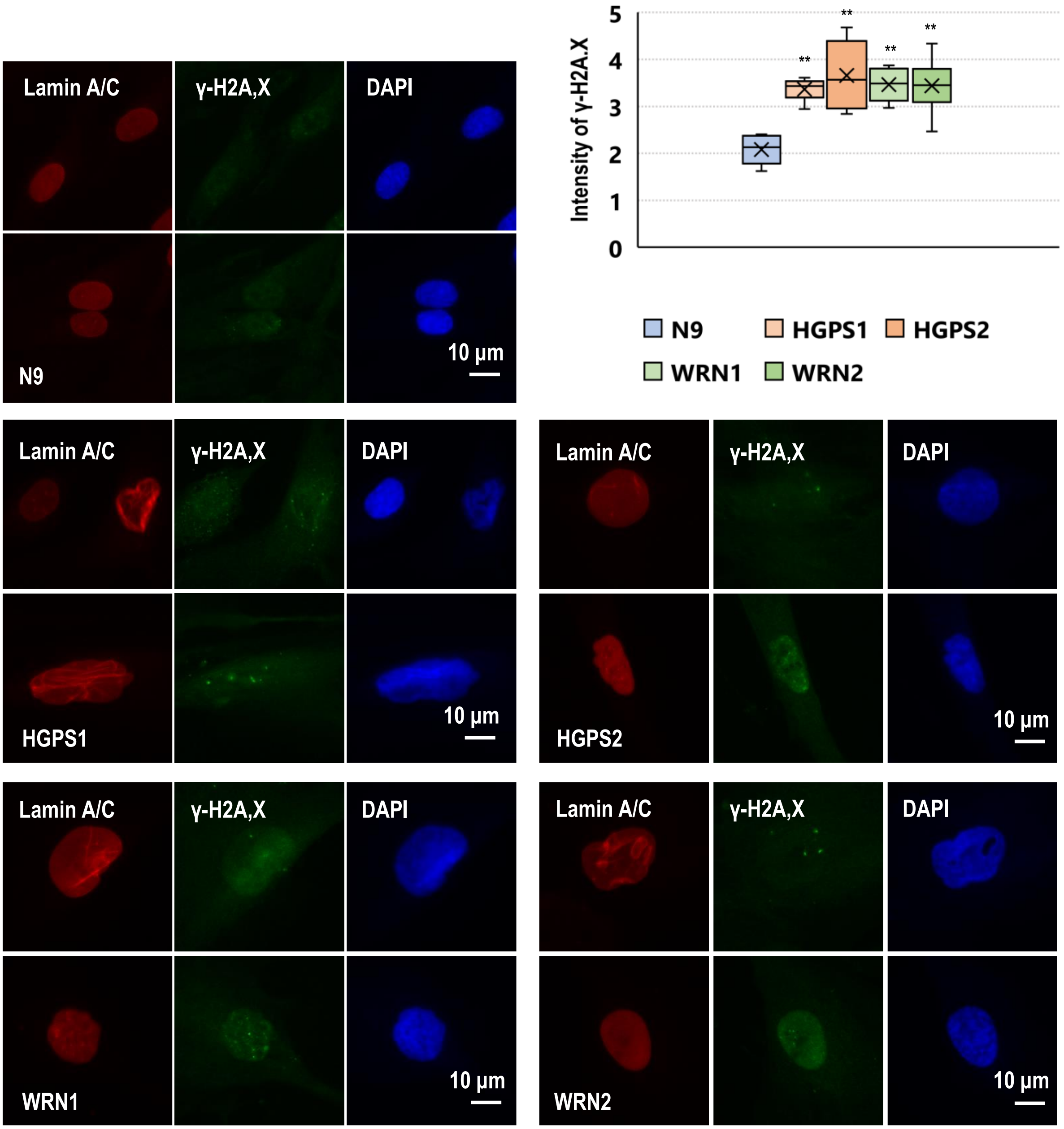

Intensity of γ-H2A.X
5
4
3
2
1
0
**
**
**
**
N9
HGPS1
HGPS2
WRN1
WRN2
Lamin A/C
γ-H2A,X
DAPI
N9
10 μm
HGPS1
HGPS2
WRN1
WRN2

F

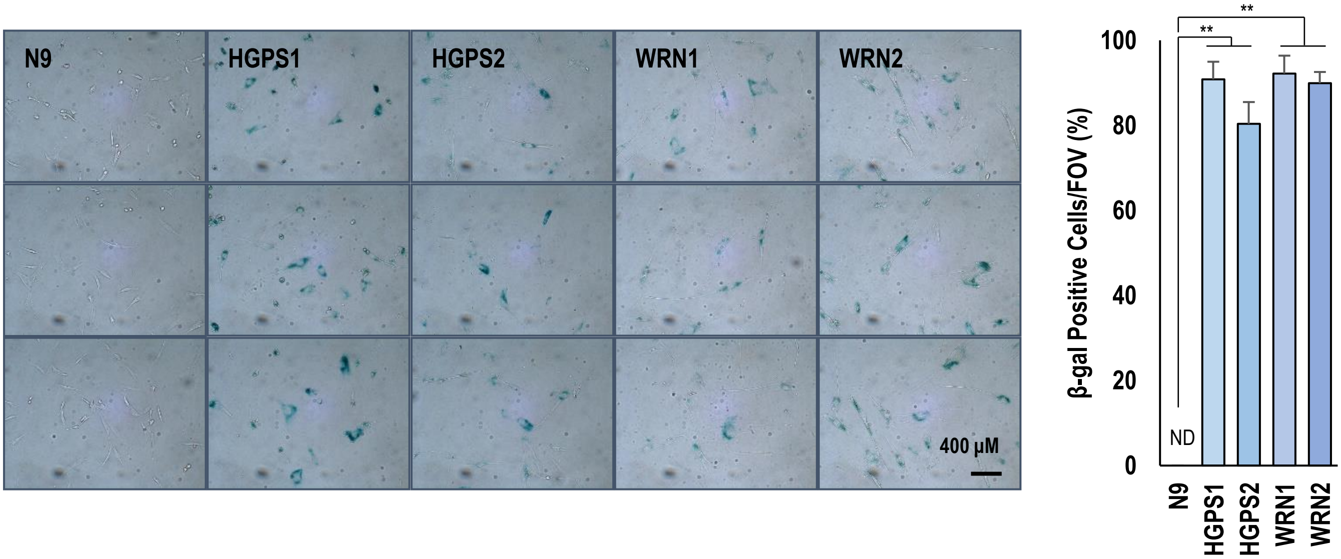
N9
HGPS1
HGPS2
WRN1
WRN2
400 μM
β-gal Positive Cells/FOV (%)
100
80
60
40
20
0
ND
**
**
N9
HGPS1
HGPS2
WRN1
WRN2

G

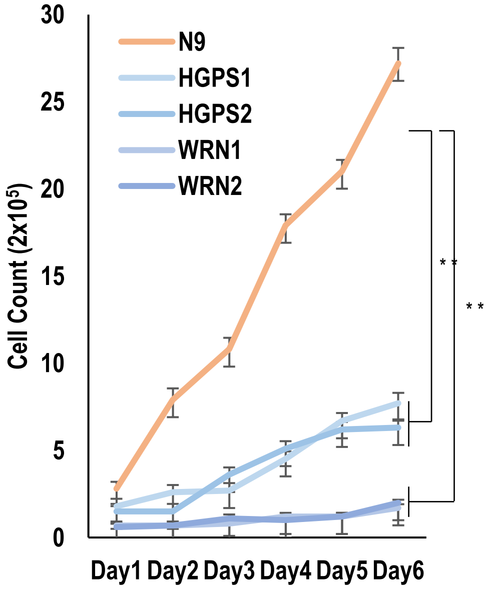
30
25
20
15
10
5
0
Cell Count (2x10⁵)
N9
HGPS1
HGPS2
WRN1
WRN2
**
**
Day1 Day2 Day3 Day4 Day5 Day6

Fig. S3. Similar senescence features in HGPS and WRN fibroblasts. **A.** Expression of Ki-67 in N9, HGPS, and WRN cells. Compared to N9 cells, HGPS and WRN cells showed significantly lower levels of Ki-67 expression. Cells were stained with anti-Ki-67 antibody (n = 3 independent experiments; two-tailed Student's t-test). **B.** Percentage of Ki-67 positive nuclei in N9, HGPS, and WRN cells. Ki-67 positive cells were counted within the field of view (FOV). **C.** The expression of paxillin and phalloidin, markers of focal adhesion formation, was increased in HGPS and WRN cells compared to N9 cells (n = 3 independent experiments; two-tailed Student's t-test). **D.** The cell size and number of enlarged cells were much higher in HGPS and WRN cells. Cells were counted within one FOV (n = 3 independent experiments; two-tailed Student's t-test). **E.** Basal level of γ-H2A.X expression in N9, HGPS, and WRN cells. The box plot shows the intensity of γ-H2A.X. Cells were stained with anti-lamin A/C and anti- γ-H2A.X antibodies (n = 3 independent experiments; two-tailed Student's t-test). **F.** SA-β-Gal expression was increased in HGPS and WRN cells compared to N9 cells (left boxes). The bar graph (right) shows the percentage of SA-β-Gal positive cells within the FOV. Five FOVs were counted in measurements (n = 3 independent experiments; two-tailed Student's t-test). **G.** Cell propagation curve for 5 days in N9, HGPS, and WRN cells. Unlike normal young fibroblasts, HGPS and WRN cells proliferated very slowly (n = 3 independent experiments; two-tailed Student's t-test). All primary fibroblasts were examined at passage 11. *p < 0.05, **p <0.001, N.D: not detected. Data are mean ± SD.

Figure S4

A.

HGPS cDNA, obtained from Fig. S3A (Subject)

```
Query  1    CAACCTGCGCTCGCGCACCGTGCTGTGCGGGACCTGCGGGCAGCCTGCCGACAAGGCATC  60
            ||||||||||||||||||||||||||||||||||||||||||||||||||||||||||||
Sbjct  1    CAACCTGCGCTCGCGCACCGTGCTGTGCGGGACCTGCGGGCAGCCTGCCGACAAGGCATC  60

Query  61   TGCCAGCGGCTCAGGAGCCCAGGTGGGCGGACCCATCTCCTCTGGCTCTTCTGCCTCCAG  120
            ||||||||||||||||||||||
Sbjct  61   TGCCAGCGGCTCAGGAGCCCAG--------------------------------------  82

Query  121  TGTCACGGTCACTCGCAGCTACCGCAGTGTGGGGGGCAGTGGGGTGGCAGCTTCGGGGA  180

Sbjct       ------------------------------------------------------------

Query  181  CAATCTGGTCACCCGCTCCTACCTCCTGGGCAACTCCAGCCCCGAACCCAGAGCCCCCA  240
                                                                 |||||||
Sbjct  83   -----------------------------------------------------AGCCCCA  90

Query  241  GAACTGCAGCATCATGTAA  259
            |||||||||||||||||||
Sbjct  91   GAACTGCAGCATCATGTAA  109
```

cDNA (WRN1)

```
Query  1    CAACCTGCGCTCGCGCACCGTGCTGTGCGGGACCTGCGGGCAGCCTGCCGACAAGGCATC  60
            ||||||||||||||||||||||||||||||||||||||||||||||||||||||||||||
Sbjct  1    CAACCTGCGCTCGCGCACCGTGCTGTGCGGGACCTGCGGGCAGCCTGCCGACAAGGCATC  60

Query  61   TGCCAGCGGCTCAGGAGCCCAGGTGGGCGGACCCATCTCCTCTGGCTCTTCTGCCTCCAG  120
            ||||||||||||||||||||||
Sbjct  61   TGCCAGCGGCTCAGGAGCCCAG--------------------------------------  82

Query  121  TGTCACGGTCACTCGCAGCTACCGCAGTGTGGGGGGCAGTGGGGTGGCAGCTTCGGGGA  180

Sbjct       ------------------------------------------------------------

Query  181  CAATCTGGTCACCCGCTCCTACCTCCTGGGCAACTCCAGCCCCGAACCCAGAGCCCCCA  240
                                                                 |||||||
Sbjct  83   -----------------------------------------------------AGCCCCA  90

Query  241  GAACTGCAGCATCATGTAA  259
            |||||||||||||||||||
Sbjct  91   GAACTGCAGCATCATGTAA  109
```

cDNA (WRN2)

```
Query  1    CAACCTGCGCTCGCGCACCGTGCTGTGCGGGACCTGCGGGCAGCCTGCCGACAAGGCATC  60
            ||||||||||||||||||||||||||||||||||||||||||||||||||||||||||||
Sbjct  1    CAACCTGCGCTCGCGCACCGTGCTGTGCGGGACCTGCGGGCAGCCTGCCGACAAGGCATC  60

Query  61   TGCCAGCGGCTCAGGAGCCCAGGTGGGCGGACCCATCTCCTCTGGCTCTTCTGCCTCCAG  120
            ||||||||||||||||||||||
Sbjct  61   TGCCAGCGGCTCAGGAGCCCAG--------------------------------------  82

Query  121  TGTCACGGTCACTCGCAGCTACCGCAGTGTGGGGGGCAGTGGGGTGGCAGCTTCGGGGA  180

Sbjct       ------------------------------------------------------------

Query  181  CAATCTGGTCACCCGCTCCTACCTCCTGGGCAACTCCAGCCCCGAACCCAGAGCCCCCA  240
                                                                 |||||||
Sbjct  83   -----------------------------------------------------AGCCCCA  90

Query  241  GAACTGCAGCATCATGTAA  259
            |||||||||||||||||||
Sbjct  91   GAACTGCAGCATCATGTAA  109
```

cDNA (WRN3)

```
Query  1    CAACCTGCGCTCGCGCACCGTGCTGTGCGGGACCTGCGGGCAGCCTGCCGACAAGGCATC  60
            ||||||||||||||||||||||||||||||||||||||||||||||||||||||||||||
Sbjct  1    CAACCTGCGCTCGCGCACCGTGCTGTGCGGGACCTGCGGGCAGCCTGCCGACAAGGCATC  60

Query  61   TGCCAGCGGCTCAGGAGCCCAGGTGGGCGGACCCATCTCCTCTGGCTCTTCTGCCTCCAG  120
            ||||||||||||||||||||||
Sbjct  61   TGCCAGCGGCTCAGGAGCCCAG--------------------------------------  82

Query  121  TGTCACGGTCACTCGCAGCTACCGCAGTGTGGGGGGCAGTGGGGTGGCAGCTTCGGGGA  180

Sbjct       ------------------------------------------------------------

Query  181  CAATCTGGTCACCCGCTCCTACCTCCTGGGCAACTCCAGCCCCGAACCCAGAGCCCCCA  240
                                                                 |||||||
Sbjct  83   -----------------------------------------------------AGCCCCA  90

Query  241  GAACTGCAGCATCATGTAA  259
            |||||||||||||||||||
Sbjct  91   GAACTGCAGCATCATGTAA  109
```

B

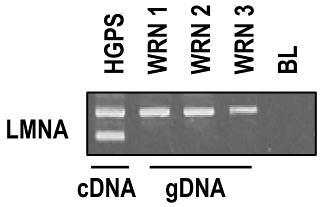

C

genomic DNA

exon 11- exon 12 (LMNA)

CAG/GTGGGC	Normal	LMNA
CAG/GTGGGT	G608G	LMNA (HGPS)
CAG/GTGGGC	Normal	LMNA (WRN)

Fig. S4. Expression of progerin in WRN cells at the transcriptional level. **A.** Alignment of progerin PCR products from cDNA showed alternative splicing in WRN fibroblasts. The sequence of purified progerin cDNA was aligned to the genome sequence of *LMNA* exon 11 (blue) to exon 12 (orange, query row) showing a gap of 150 bp between exon 11 and exon 12 in WRN fibroblasts. All fibroblasts were analyzed at passage 12. **B.** Analysis of lamin A genomic DNA (gDNA) in WRN cells (at passage 12). An intronic primer was designed for the PCR amplification of the *LMNA* exon 11 in WRN cells. The PCR product of HGPS cells was generated by amplification of cDNA using exon-specific primers (n = 3 independent experiments; two-tailed Student's t-test). **C.** The sequence of the PCR products generated by amplification of genomic DNA in WRN and HGPS fibroblasts. Shown are the splice sequence at the end of exon 11, the sequence of the normal *LMNA* splice site in WRN fibroblasts as in normal fibroblasts and the sequence commonly mutated at splice site in HGPS fibroblasts.

Figure S5

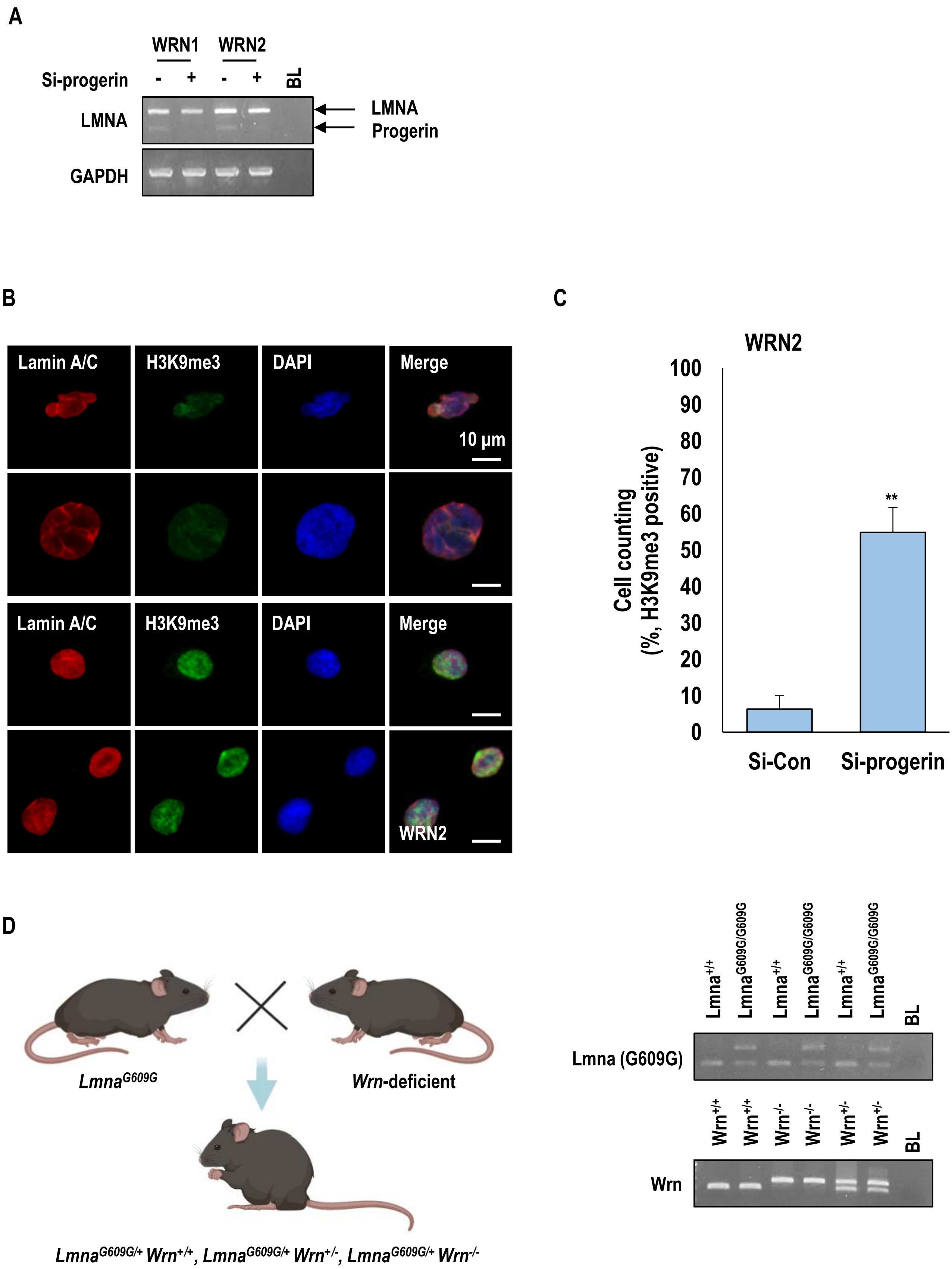
A
WRN1
WRN2
Si-progerin
-
+
-
+
BL
LMNA
GAPDH
LMNA
Progerin
B
C
Lamin A/C
H3K9me3
DAPI
Merge
10 μm
Si-Con
Si-progerin
WRN2
WRN2
Cell counting
(%, H3K9me3 positive)
100
90
80
70
60
50
40
30
20
10
0
**
Si-Con
Si-progerin
D
Lmna^G609G
Wrn-deficient
Lmna^G609G/+ Wrn^+/+, Lmna^G609G/+ Wrn^+/-, Lmna^G609G/+ Wrn^-/-
Lmna^+/+
Lmna^G609G/G609G
Lmna^+/+
Lmna^G609G/G609G
Lmna^+/+
Lmna^G609G/G609G
BL
Lmna (G609G)
Wrn^+/+
Wrn^+/+
Wrn^-/-
Wrn^-/-
Wrn^+/-
Wrn^+/-
BL
Wrn

Fig. S5. Progerin is an influential factors in WRN. **A.** The expression of progerin mRNA was reduced after transfection with siRNA for 48 hr. **B.** Expression of H3K9me3 after transfection with siRNA to reduce progerin expression in WRN cells. Knockdown of progerin using siRNA induced H3K9me3 and reduced nuclear size in WRN cells. WRN cells (at passage 11) were transfected with Si-con (nontarget sequence) or Si-progerin for 48 hr and stained with anti-lamin A/C and anti-H3K9me3 antibodies and DAPI (n = 3 independent experiments; unpaired t-test). **C.** The graph shows the percentage of H3K9me3-positive cells after progerin expression was decreased using siRNA in WRN cells (n = 3 independent experiments; unpaired t-test). **D.** Genotypes of mouse models. $Lmna^{G609G}$ progeroid mice were crossbred with *Wrn*-deficient mice (left). Genotypes of mouse models were verified by using genomic DNA and specific primers (right). **p <0.001. Data are mean ± SD.

Figure S6

A **Human WRN (Query)**

Mouse WRN (Subject)

```
Query  366  KVERKEDGFEDGVEDNKLKENMERACLMSLDITEHELQILEQQSQEEYLSDIAYKSTEHL  425
            +V++++    E+ +EDN L+E+MER C++    I+E+ELQ LEQQ++EE  +D++++ +EHL
Sbjct  360  QVKQEKGESENEIEDNLLREDMERTCVIP-SISENELQDLEQQAKEEKYNDVSHQLSEHL  418

Query  426  SPNDNENDTSYVIESDEDLEMEMLKHLSPNDNENDTSYVIESDEDLEMEMLKSLENLNSG  485
            SPND+END+SY+IESDEDLEM                         EMLKSLENLNS
Sbjct  419  SPNDDENDSSYIIESDEDLEM-------------------------EMLKSLENLNSD  451

Query  486  TVEPTHSKCLKMERNLGLPTKEEEEDDENEANEGEEDDDKDFLWPAPNEEQVTCLKMYFG  545
             VEPTHSK L+M  N  LP  EEE+    NEA + EE +++D L P PN +Q+ CLK YFG
Sbjct  452  MVEPTHSKWLEMGTNGCLP-PEEEDGHGNEAIK-EEQEEEDHLLPEPNAKQINCLKTYFG  509
```

B **Human WRN cDNA**

```
1141 aacaaattga aagagaatat ggaaagagct tgtttgatgt cgttagatat tacagaacat
1201 gaactccaaa ttttggaaca gcagtctcag gaagaatatc ttagtgatat tgcttataaa
1261 tctactgagc atttatctcc caatgataat gaaaacgata cgtcctatgt aattgagagt
1321 gatgaagatt tagaaatgga gatgcttaag catttatctc ccaatgataa tgaaaacgat
1381 acgtcctatg taattgagag tgatgaagat ttagaaatgg agatgcttaa gtctttagaa
1441 aacctcaata gtggcacggt agaaccaact cattctaaat gcttaaaaat ggaaagaaat
1501 ctgggtcttc ctactaaaga agaagaagaa gatgatgaaa atgaagctaa tgaaggggaa
1561 gaagatgatg ataaggactt tttgtggcca gcacccaatg aagagcaagt tacttgcctc
1621 aagatgtact ttggccattc cagttttaaa ccagttcagt ggaaagtgat tcattcagta
1681 ttagaagaaa gaagagataa tgttgctgtc atggcaactg gatatggaaa gagtttgtgc
1741 ttccagtatc cacctgttta tgtaggcaag attggccttg ttatctctcc ccttatttct
1801 ctgatggaag accaagtgct acagcttaaa atgtccaaca tcccagcttg cttccttgga
1861 tcagcacagt cagaaaatgt tctaacagat attaaattag gtaaataccg gattgtatac
1921 gtaactccag aatactgttc aggtaacatg ggcctgctcc agcaacttga ggctgatatt
```

Exon9 (Black line)-Exon 10 (Red line)

C

WRN-R1: HLSPNDNENDTSYVIESDEDLEMEMLK

WRN-R2: HLSPNDNENDTSYVIESDEDLEMEMLK HLSPNDNENDTSYVIESDEDLEMEMLK

Fig. S6. hWRN has a unique repeated sequence, which is different from mouse WRN. **A**. Twenty-eight amino acids (black underlined region) are repeated in the human sequence (red underlined region). **B.** In cDNA analysis, coding cDNA sequences for repeated amino acid sequences are also duplicated. The black underlined region indicates exon 9, and the red underlined region indicates exon 10 of the hWRN. **C**. Amino acid sequence of recombinant WRN-R1 (unrepeated peptide; 28 AA) and WRN-R2 (repeated peptide; 56 AA) proteins.

Figure S7

A

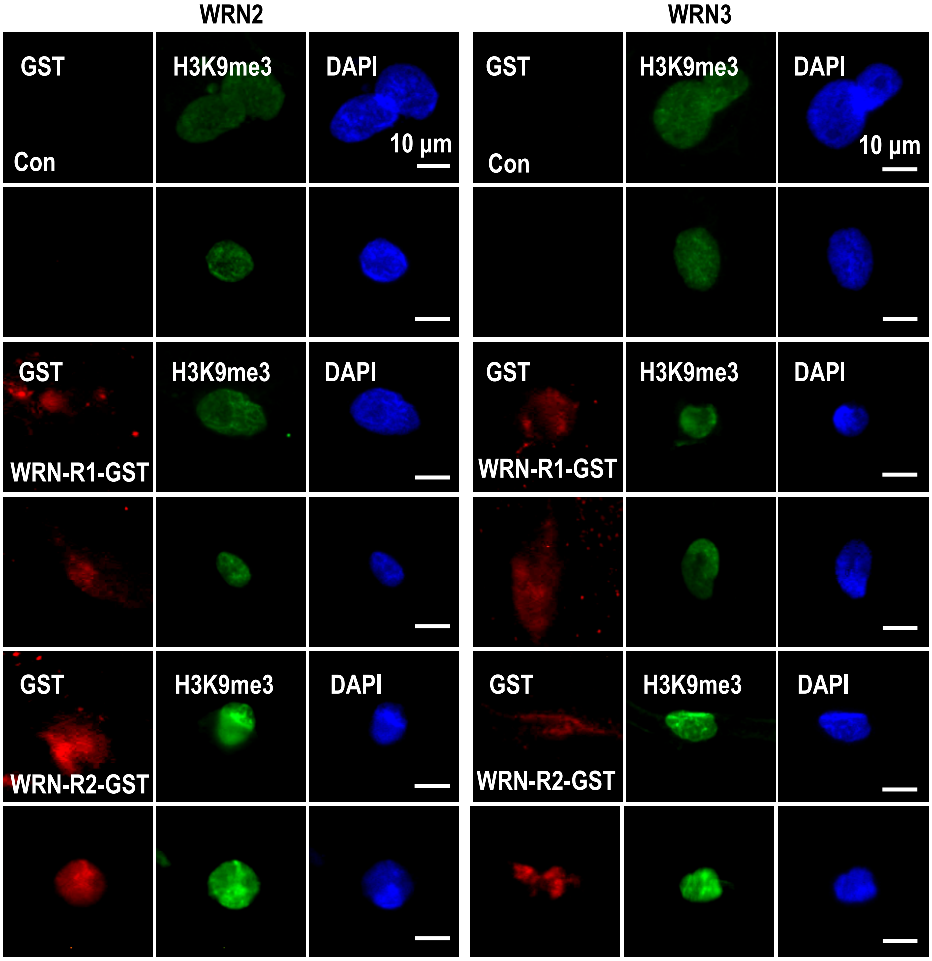
WRN2
WRN3
GST
H3K9me3
DAPI
Con
10 μm
WRN-R1-GST
WRN-R2-GST

B

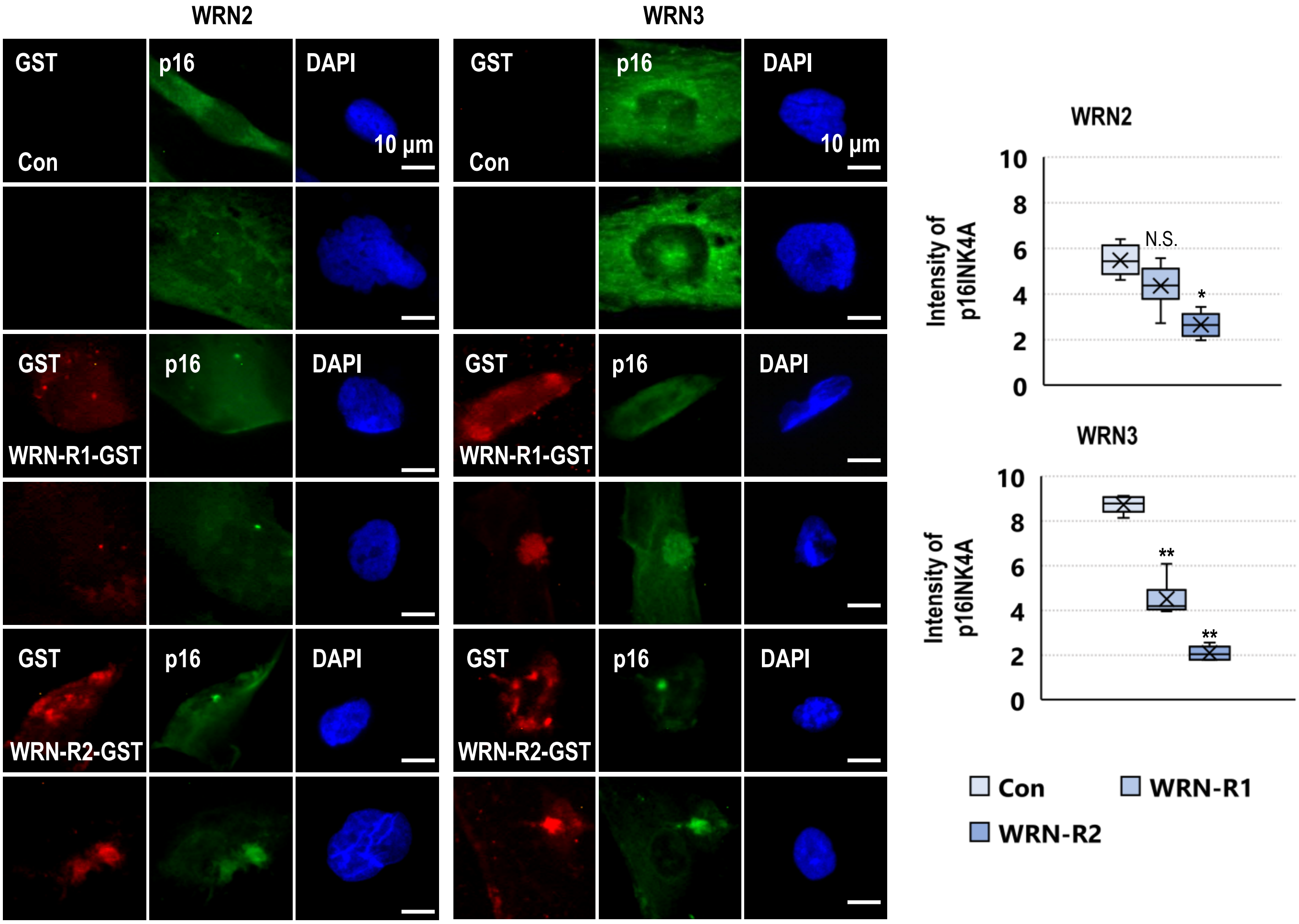
WRN2
WRN3
GST
p16
DAPI
Con
10 μm
WRN-R1-GST
WRN-R2-GST
Intensity of p16INK4A
N.S.
*
**
Con
WRN-R1
WRN-R2

C

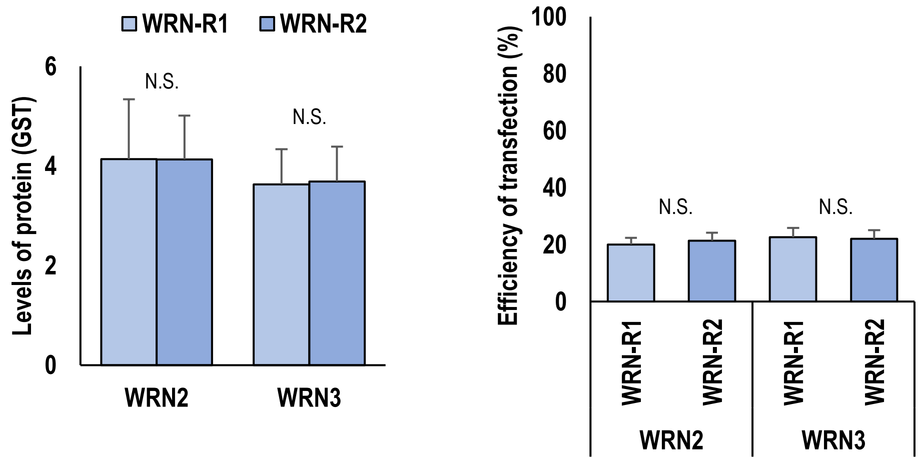

WRN-R1
WRN-R2
Levels of protein (GST)
6
4
2
0
N.S.
N.S.
WRN2
WRN3
Efficiency of transfection (%)
100
80
60
40
20
0
N.S.
N.S.
WRN-R1
WRN-R2
WRN-R1
WRN-R2
WRN2
WRN3

D

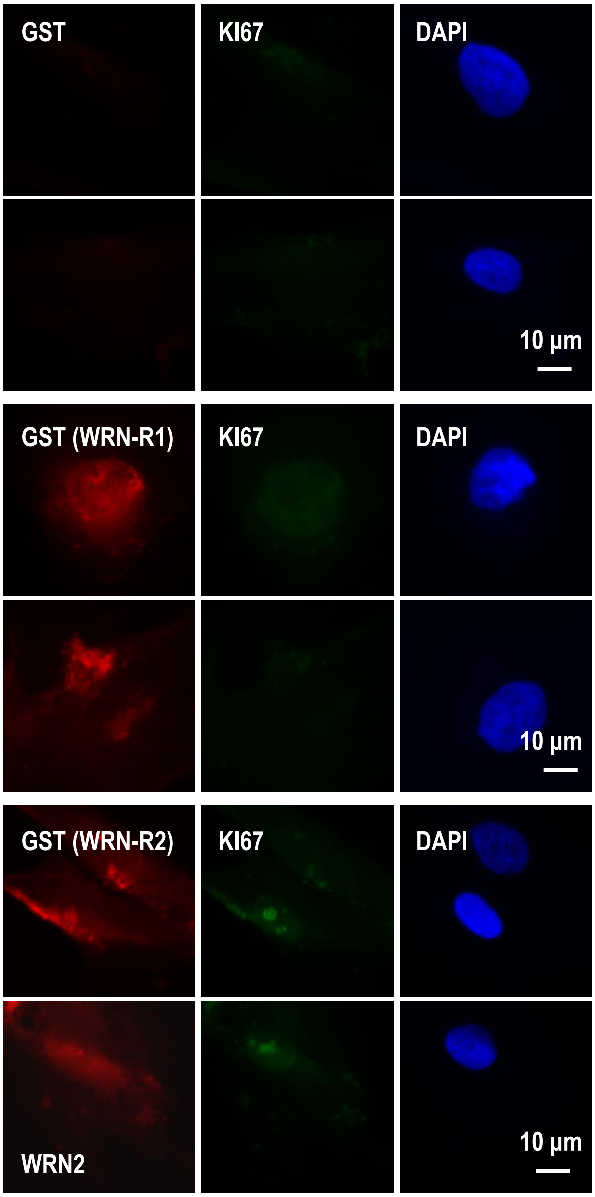

GST
KI67
DAPI
10 μm
GST (WRN-R1)
KI67
DAPI
10 μm
GST (WRN-R2)
KI67
DAPI
WRN2
10 μm

E

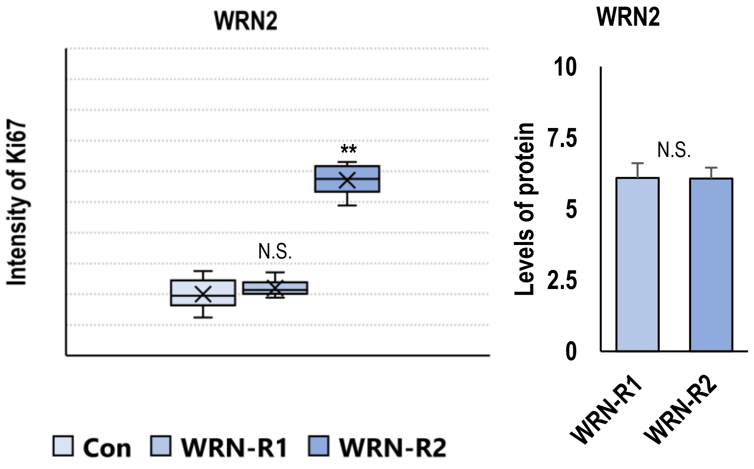

WRN2
Intensity of Ki67
**
N.S.
Con
WRN-R1
WRN-R2
WRN2
10
7.5
5
2.5
0
Levels of protein
N.S.
WRN-R1
WRN-R2

Fig. S7. WRN-R2 rescues aging-related markers in WRN fibroblasts. **A.** GST-tagged WRN-R1 and WRN-R2 recombinant proteins were delivered into WRN cells for 24 hr. WRN-R2 induced H3K9me3 in WRN cells. Cells were stained with anti-GST and anti-H3K9me3 antibodies and DAPI. (n = 3 independent experiments; unpaired t-test). **B.** WRN-R2 reduced p16INK4A expression in WRN cells. Delivery of WRN-R2, but not WRN-R1, obviously reduced p16INK4A expression in WRN cells. Cells were stained with anti-GST and anti-p16INK4A antibodies and DAPI. The box plot shows the intensity of p16INK4A expression after the delivery of recombinant proteins (n = 3 independent experiments; unpaired t-test). **C.** The graph shows the levels of delivered recombinant proteins (left) and percentage of transfection efficiency (right) in WRN cells. **D.** GST-tagged WRN-R1 and WRN-R2 recombinant proteins were transfected into WRN cells. WRN-R2 induced Ki67 expression in WRN cells. Cells were stained with anti-GST and anti-Ki67 antibodies and DAPI. **E.** The box plot and bar graph show the intensity of Ki67 expression (left) and levels of transferred proteins (right) in WRN cells (n = 3 independent experiments; unpaired t-test). All fibroblasts were examined at passage 10. $^{**}p$ <0.001, N.S: not significant. Data are mean ± SD.

Figure S8

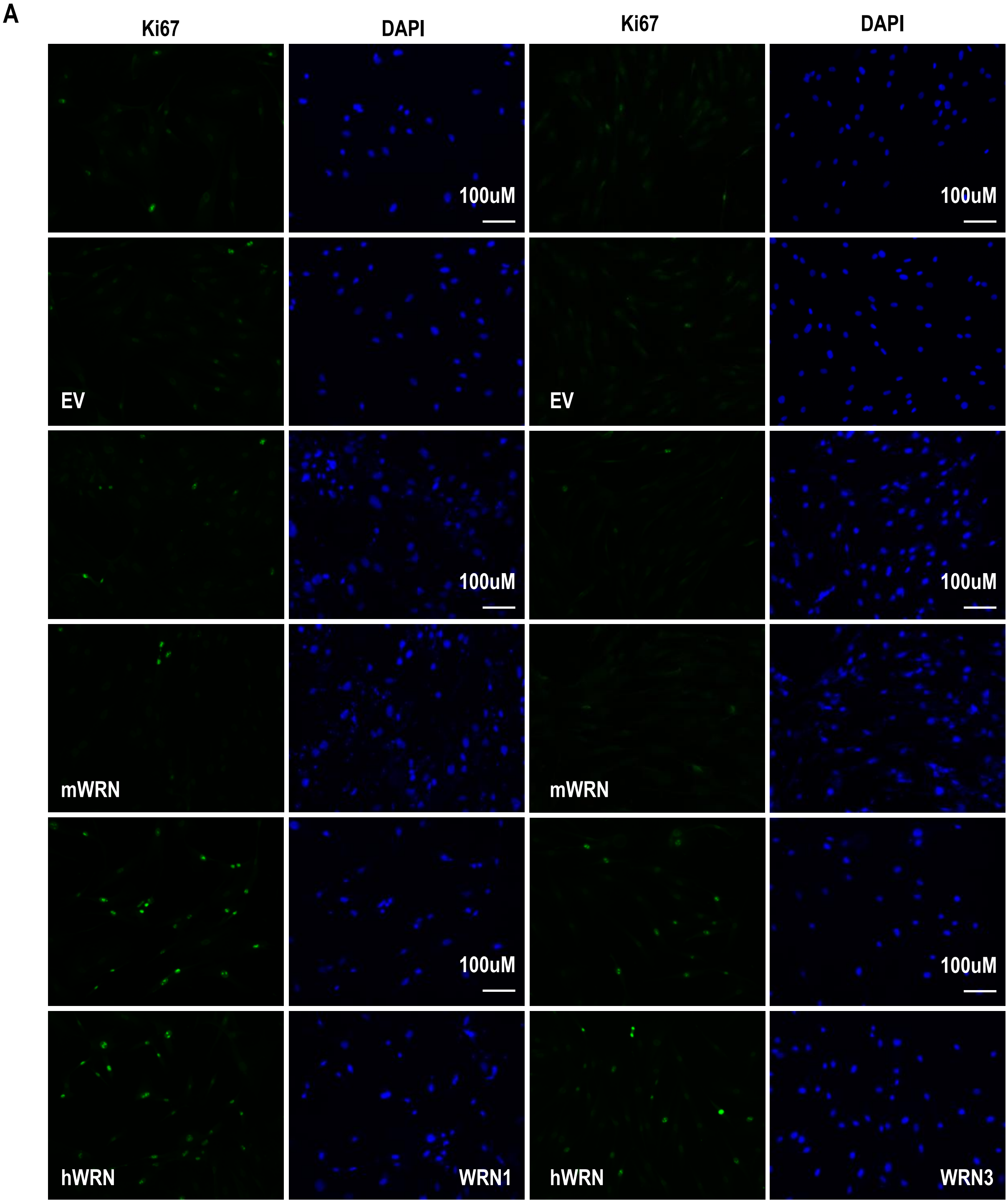
A
Ki67
DAPI
Ki67
DAPI
100uM
100uM
EV
EV
100uM
100uM
mWRN
mWRN
100uM
100uM
hWRN
WRN1
hWRN
WRN3

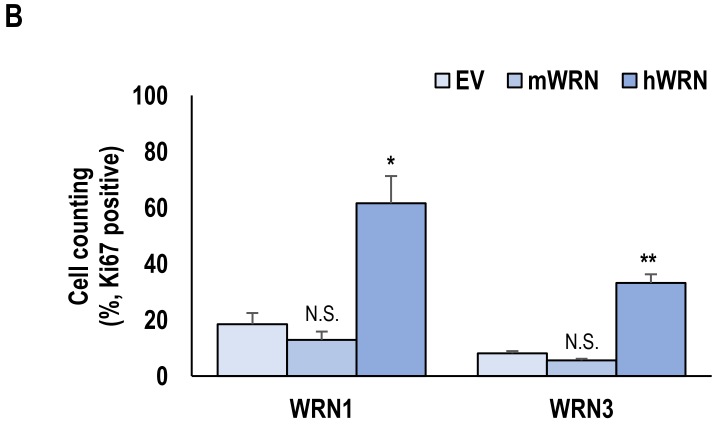
B
EV
mWRN
hWRN
Cell counting (%, Ki67 positive)
100
80
60
40
20
0
*
**
N.S.
N.S.
WRN1
WRN3

C

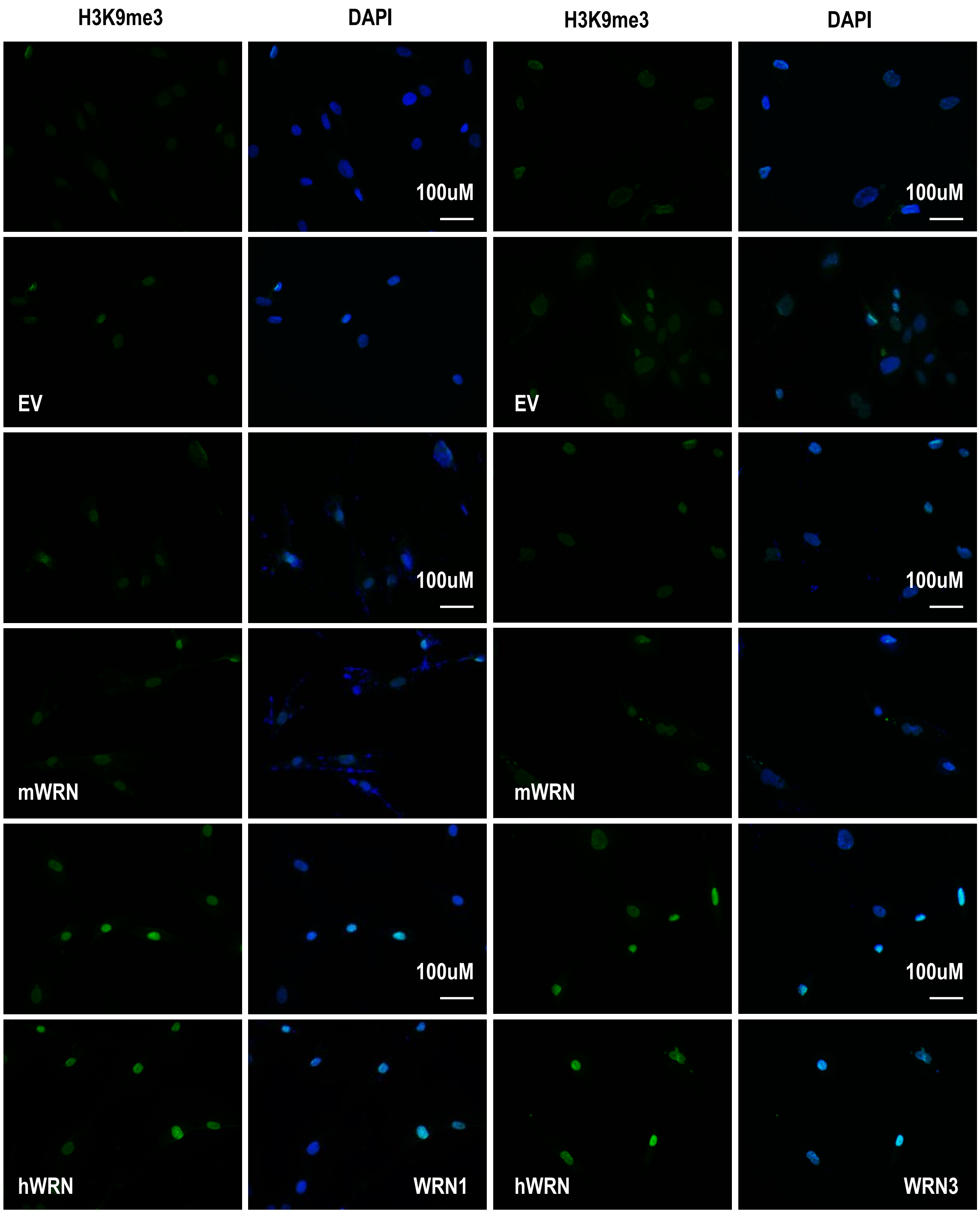
H3K9me3
DAPI
H3K9me3
DAPI
100uM
EV
EV
mWRN
mWRN
hWRN
WRN1
hWRN
WRN3

D

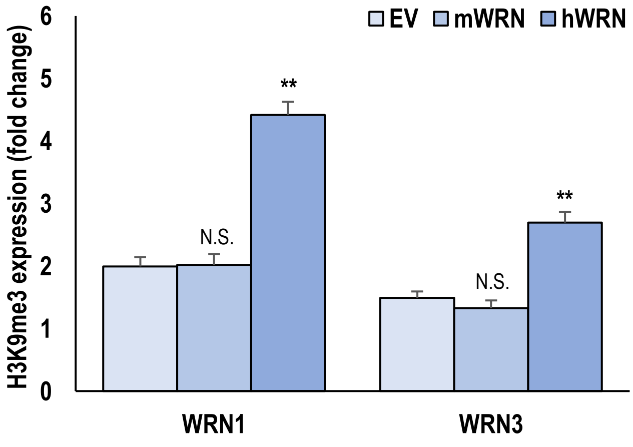
EV mWRN hWRN
H3K9me3 expression (fold change)
6
5
4
3
2
1
0
**
N.S.
**
N.S.
WRN1
WRN3

E

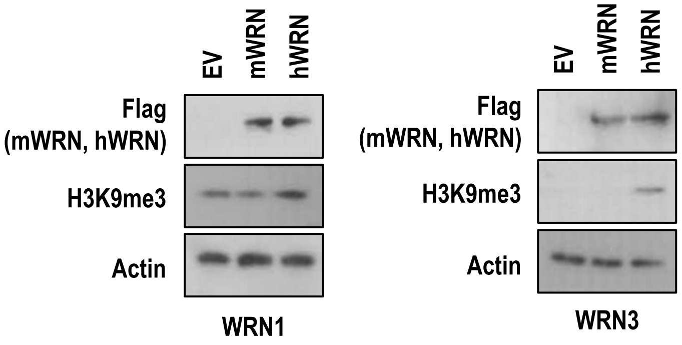
EV
mWRN
hWRN
Flag
(mWRN, hWRN)
H3K9me3
Actin
WRN1
WRN3

F

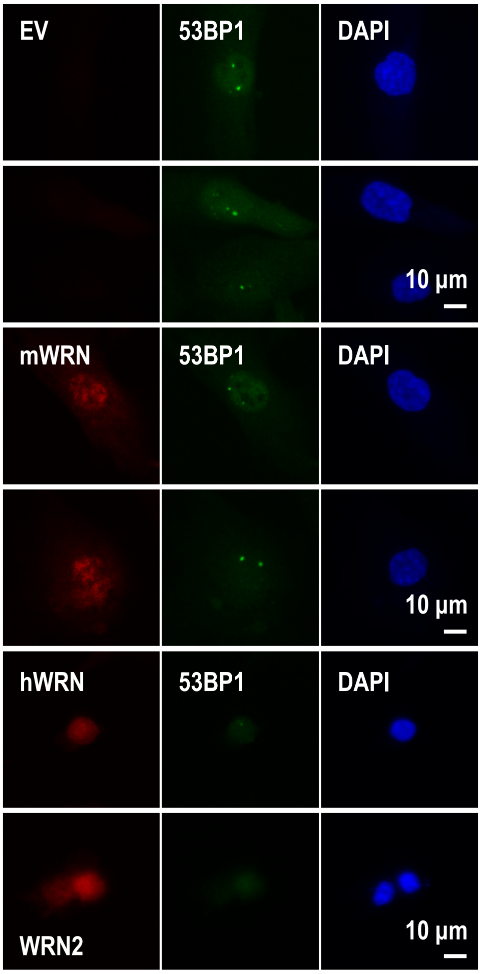
EV
53BP1
DAPI
10 μm
mWRN
53BP1
DAPI
10 μm
hWRN
53BP1
DAPI
WRN2
10 μm

H

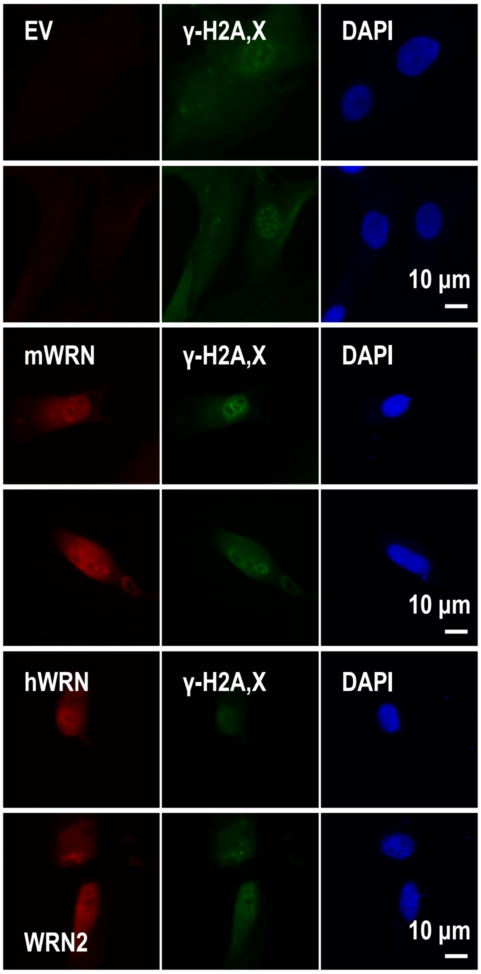
EV
γ-H2A,X
DAPI
10 μm
mWRN
γ-H2A,X
DAPI
10 μm
hWRN
γ-H2A,X
DAPI
WRN2
10 μm

G

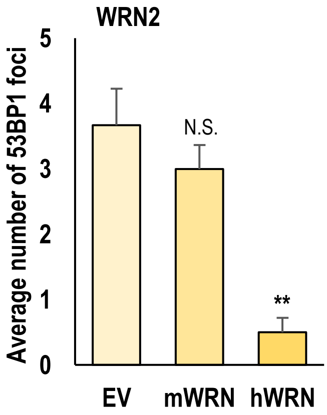
WRN2
Average number of 53BP1 foci
0
1
2
3
4
5
N.S.
**
EV
mWRN
hWRN

I

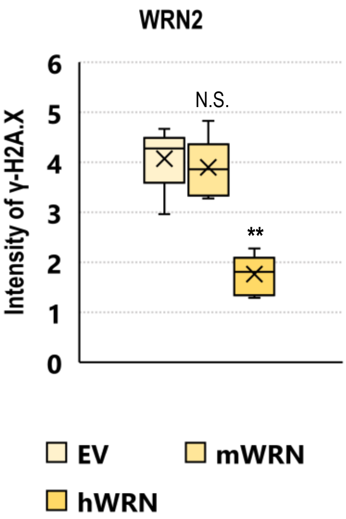
WRN2
Intensity of γ-H2A.X
0
1
2
3
4
5
6
N.S.
**
EV
mWRN
hWRN

J

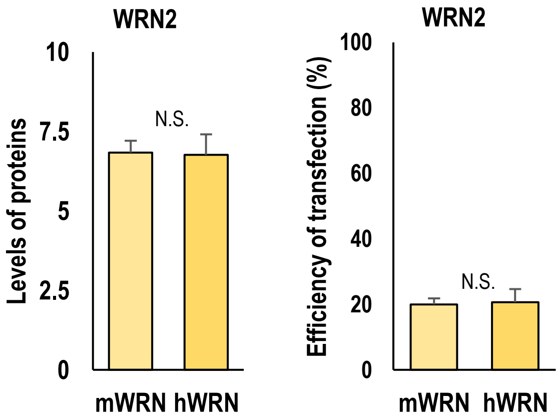
WRN2
Levels of proteins
0
2.5
5
7.5
10
N.S.
mWRN hWRN
WRN2
Efficiency of transfection (%)
0
20
40
60
80
100
N.S.
mWRN hWRN

Fig. S8. Human WRN induces cell proliferation and ameliorates senescence in WRN fibroblasts. **A.** WRN cells were transfected with vectors expressing hWRN and mWRN for 48 hr. EV refers to empty vector, which is used as a negative control. Transfection of hWRN, but not mWRN, increased Ki67-positive cells in WRN cells. Cells were stained with anti-Ki67 antibody and DAPI (n = 3 independent experiments; unpaired t-test). **B.** The graph shows the percentage of Ki67-positive cells after transfection. **C.** H3K9me3 expression was induced after transfection with hWRN and mWRN vectors. Cells were stained with anti-H3K9me3 antibody and DAPI. **D.** The graph shows the intensity of H3K9me3 expression after transfection with hWRN and mWRN vectors. **E.** Confirmation of transfected vectors expressing hWRN and mWRN in WRN cells by western blot. Expression of H3K9me3 was increased in hWRN-positive cells. **F.** Flag-tagged hWRN and mWRN vectors were transfected into WRN cells for 48 hr. hWRN reduced the expression of 53BP1 in WRN cells. Cells were stained with anti-flag, anti-53BP1 and DAPI. **G.** The graph shows the intensity of 53BP1 after transfection in WRN cells. **H.** hWRN reduced the expression of γ-H2A.X in WRN cells. Cells were stained with anti-flag, anti- γ-H2A.X and DAPI. **I.** The box plot shows the intensity of γ-H2A.X after transfection in WRN cells. **J.** The graphs show the levels of transfected vectors (left) and the efficiency of transfection in WRN cells. All fibroblasts were examined at passage 10. *p <0.05, **p <0.001, N.S: not significant. Data are mean ± SD.

Figure S9

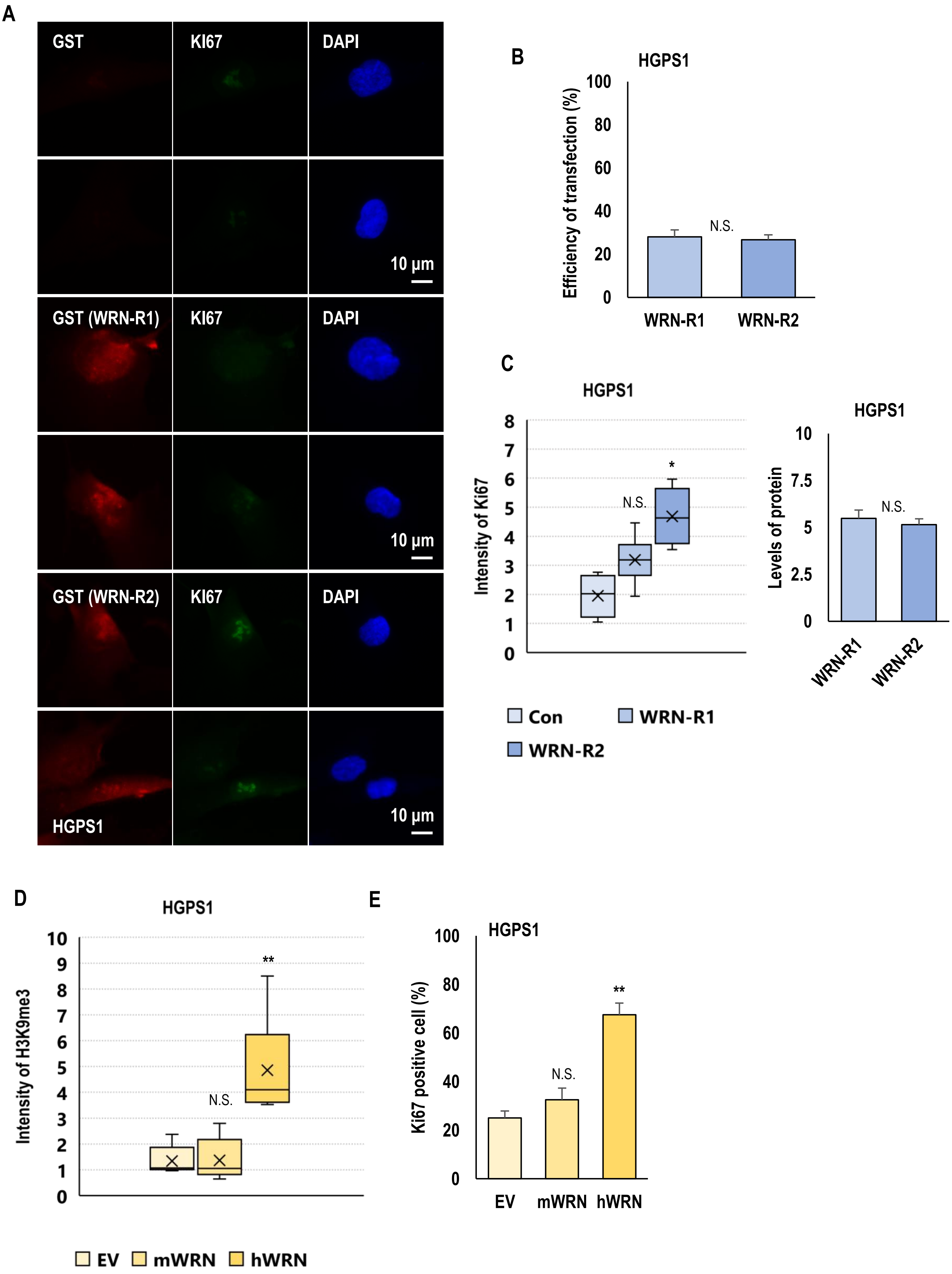

A
GST
KI67
DAPI
10 µm
GST (WRN-R1)
KI67
DAPI
10 µm
GST (WRN-R2)
KI67
DAPI
HGPS1
10 µm
B
HGPS1
Efficiency of transfection (%)
100
80
60
40
20
0
N.S.
WRN-R1
WRN-R2
C
HGPS1
Intensity of Ki67
8
7
6
5
4
3
2
1
0
N.S.
*
Con
WRN-R1
WRN-R2
HGPS1
Levels of protein
10
7.5
5
2.5
0
N.S.
WRN-R1
WRN-R2
D
HGPS1
Intensity of H3K9me3
10
9
8
7
6
5
4
3
2
1
0
N.S.
**
EV
mWRN
hWRN
E
HGPS1
Ki67 positive cell (%)
100
80
60
40
20
0
N.S.
**
EV
mWRN
hWRN

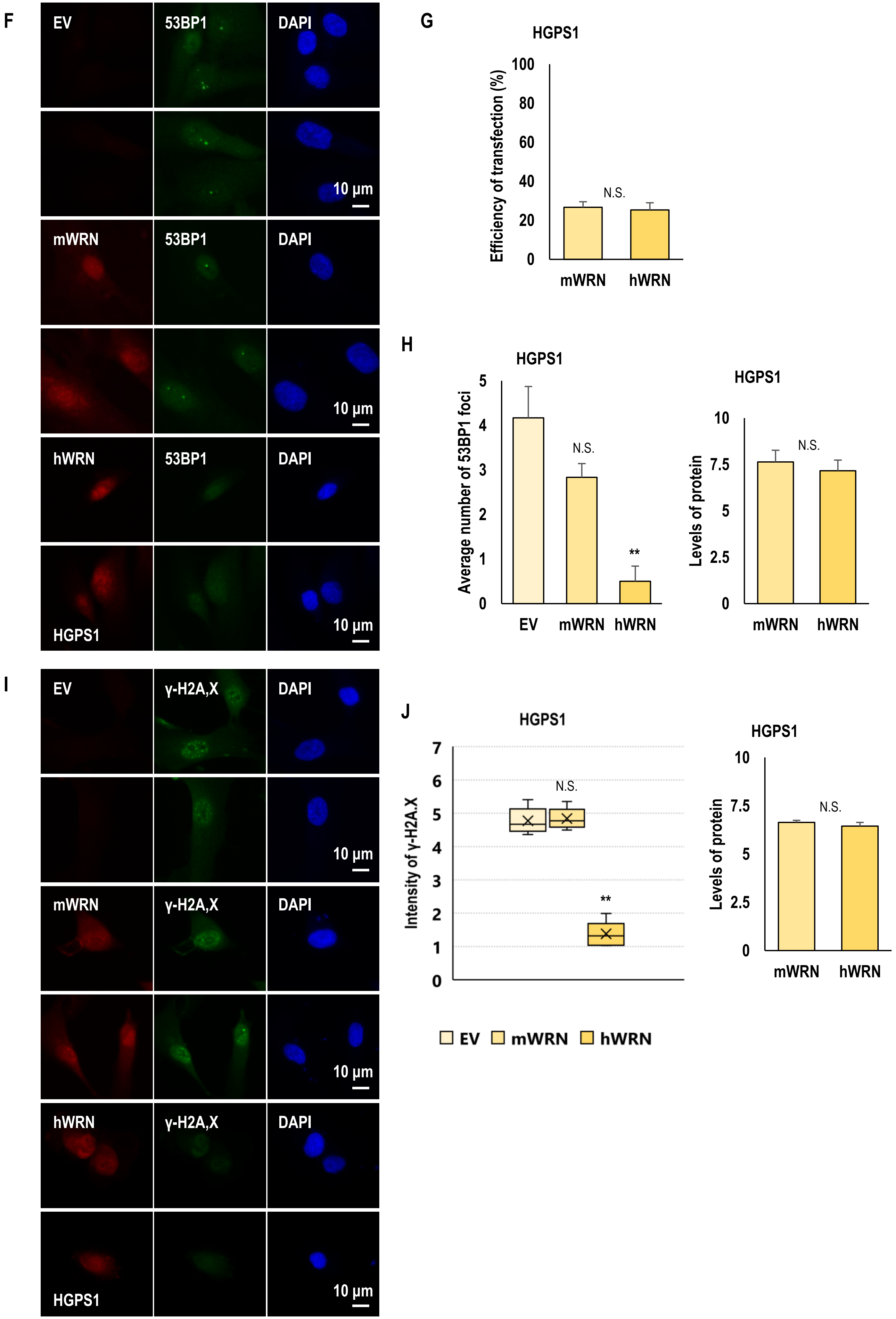
F
EV
53BP1
DAPI
10 μm
mWRN
53BP1
DAPI
10 μm
hWRN
53BP1
DAPI
HGPS1
10 μm
G
HGPS1
Efficiency of transfection (%)
100
80
60
40
20
0
N.S.
mWRN
hWRN
H
HGPS1
Average number of 53BP1 foci
5
4
3
2
1
0
N.S.
**
EV
mWRN
hWRN
HGPS1
Levels of protein
10
7.5
5
2.5
0
N.S.
mWRN
hWRN
I
EV
γ-H2A,X
DAPI
10 μm
mWRN
γ-H2A,X
DAPI
10 μm
hWRN
γ-H2A,X
DAPI
HGPS1
10 μm
J
HGPS1
Intensity of γ-H2A.X
7
6
5
4
3
2
1
0
N.S.
**
HGPS1
Levels of protein
10
7.5
5
2.5
0
N.S.
mWRN
hWRN
EV
mWRN
hWRN

K

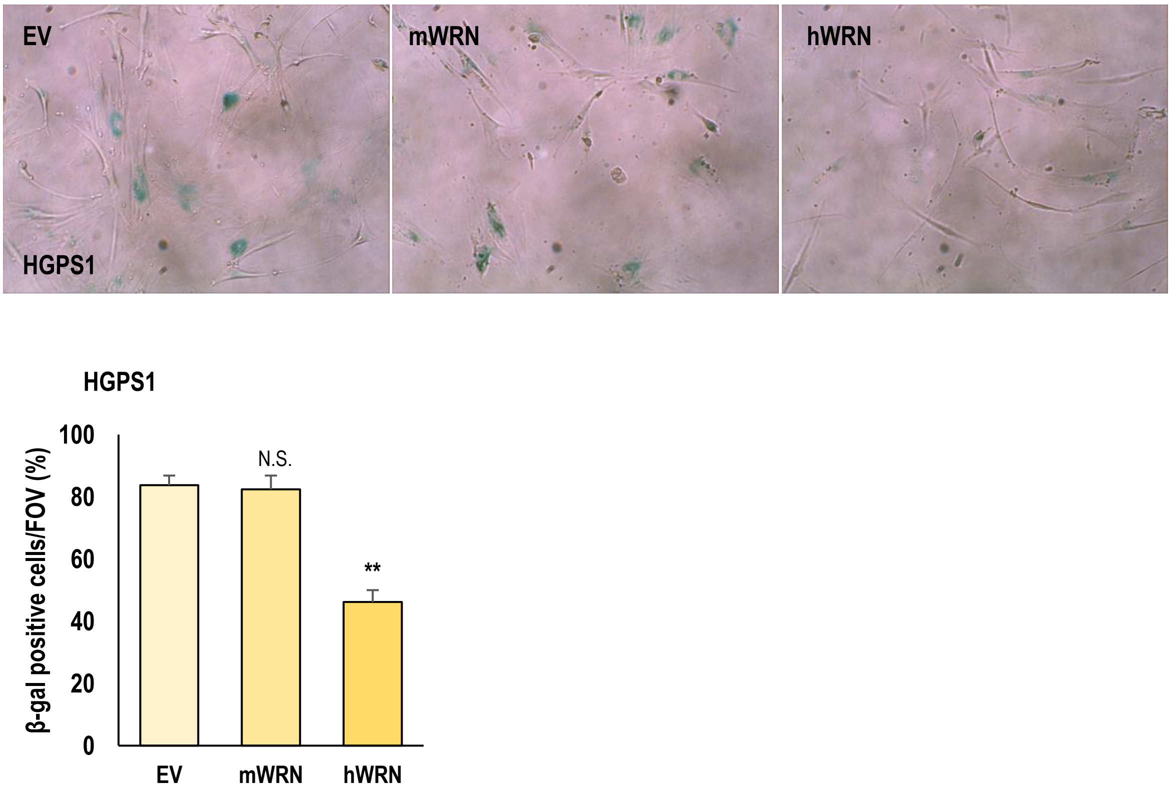
EV
mWRN
hWRN
HGPS1
HGPS1
β-gal positive cells/FOV (%)
100
80
60
40
20
0
N.S.
**
EV
mWRN
hWRN

Fig. S9. Human WRN ameliorates aging features in HGPS fibroblasts. **A.** Transfection of GST-tagged WRN-R1 and WRN-R2 recombinant proteins into HGPS cells for 24 hr. WRN-R2 induced Ki-67 expression in HGPS cells. Cells were stained with anti-GST and anti-Ki67 antibodies and DAPI (n = 3 independent experiments; unpaired t-test). **B.** The graph shows the efficiency of transfection with recombinant proteins in HGPS cells. **C.** The box plot and bar graph show the intensity of Ki67 and the levels of delivered recombinant proteins in HGPS cells (n = 3 independent experiments; unpaired t-test). **D.** The box plot shows the intensity of H3K9me3 expression after transfection with hWRN and mWRN vectors for 48 hr. **E.** The graph shows the percentage of Ki67-positive cells after transfection with hWRN and mWRN vectors. **F.** Transfection of flag-tagged hWRN and mWRN vectors into HGPS cells for 48 hr. hWRN reduced the expression of 53BP1 in HGPS cells. Cells were stained with anti-flag and anti-53BP1 antibodies and DAPI. **G.** The graph shows the efficiency of transfection with hWRN and mWRN in HGPS cells. **H.** The graphs show the intensity of 53BP1 expression (left) and levels of vector expression (right) in HGPS cells. **I.** hWRN reduced the expression of γ-H2A.X in HGPS cells. Cells were stained with anti-flag, anti- γ-H2A.X antibodies and DAPI. **J.** The box plot and bar graph show the intensity of γ-H2A.X expression (left) and levels of protein expression (right) in HGPS cells. **K.** hWRN reduced the expression of SA-β-Gal in HGPS cells compared to mWRN. The graph shows the percentage of SA-β-Gal positive cells within the FOV. Five FOVs were counted in measurements (n = 3 independent experiments; two-tailed Student's t-test). All fibroblasts were examined at passage 11. *p <0.05, **p <0.001, N.S: not significant. Data are mean ± SD.

Figure S10

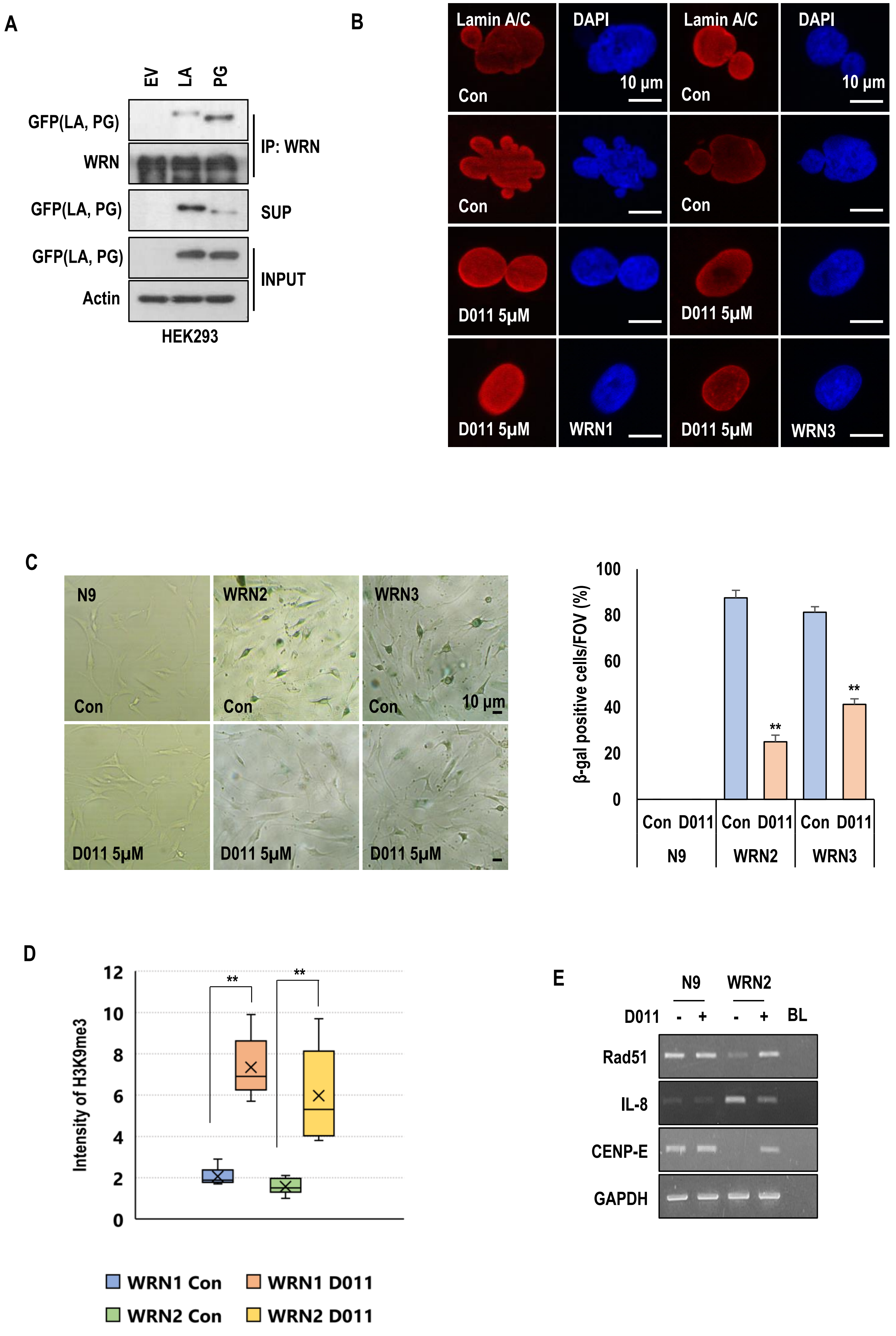
A
EV
LA
PG
GFP(LA, PG)
WRN
IP: WRN
GFP(LA, PG)
SUP
GFP(LA, PG)
Actin
INPUT
HEK293
B
Lamin A/C
DAPI
Lamin A/C
DAPI
10 μm
10 μm
Con
Con
Con
Con
D011 5μM
D011 5μM
D011 5μM
WRN1
D011 5μM
WRN3
C
N9
WRN2
WRN3
Con
Con
Con
10 μm
D011 5μM
D011 5μM
D011 5μM
β-gal positive cells/FOV (%)
100
80
60
40
20
0
**
**
Con D011
Con D011
Con D011
N9
WRN2
WRN3
D
Intensity of H3K9me3
12
10
8
6
4
2
0
**
**
WRN1 Con
WRN1 D011
WRN2 Con
WRN2 D011
E
N9
WRN2
D011
-
+
-
+
BL
Rad51
IL-8
CENP-E
GAPDH

Fig. S10. SLC-D011 can ameliorate the premature aging features of WRN cells. **A.** Immunoprecipitation assay (IP) performed using anti-WRN antibody. HEK293 lysates expressing wild-type lamin A (LA) and progerin were incubated with anti-WRN antibody for 1 hr at room temperature (RT). EV refers to empty-vector which is used as a negative control. WRN binds to progerin more strongly than lamin A. Blots were cropped from different parts of the same samples and analyzed by film-based imaging systems (n = 3 independent experiments; two-tailed Student's t-test). **B.** SLC-D011 ameliorated nuclear abnormalities in WRN cells. WRN cells (at passage 11) were incubated with SLC-D011 (5 μM) for 7 days and stained with anti-lamin A/C antibody and DAPI (n = 3 independent experiments; two-tailed Student's t-test). **C.** SA-β-Gal expression was reduced after treatment with SLC-D011 in WRN cells. WRN cells (at passage 11) were incubated with SLC-D011 for 72 hr. The bar graph shows the percentage of SA-β-Gal-positive cells within the FOV. Five FOVs were counted in measurements (n = 3 independent experiments; two-tailed Student's t-test). **D.** The box plot shows the intensity of H3K9me3 expression after treatment with SLC-D011 in WRN cells (n = 3 independent experiments; two-tailed Student's t-test). **E.** SLC-D011 induced CENP1 and Rad51 expression in WRN cells. Normal fibroblasts and WRN cells (at passage 10) were incubated with SLC-D011 for 7 days and subjected to RT-PCR (n = 3 independent experiments; unpaired t-test). $^{**}p < 0.001$. Data are mean ± SD.

Figure S11

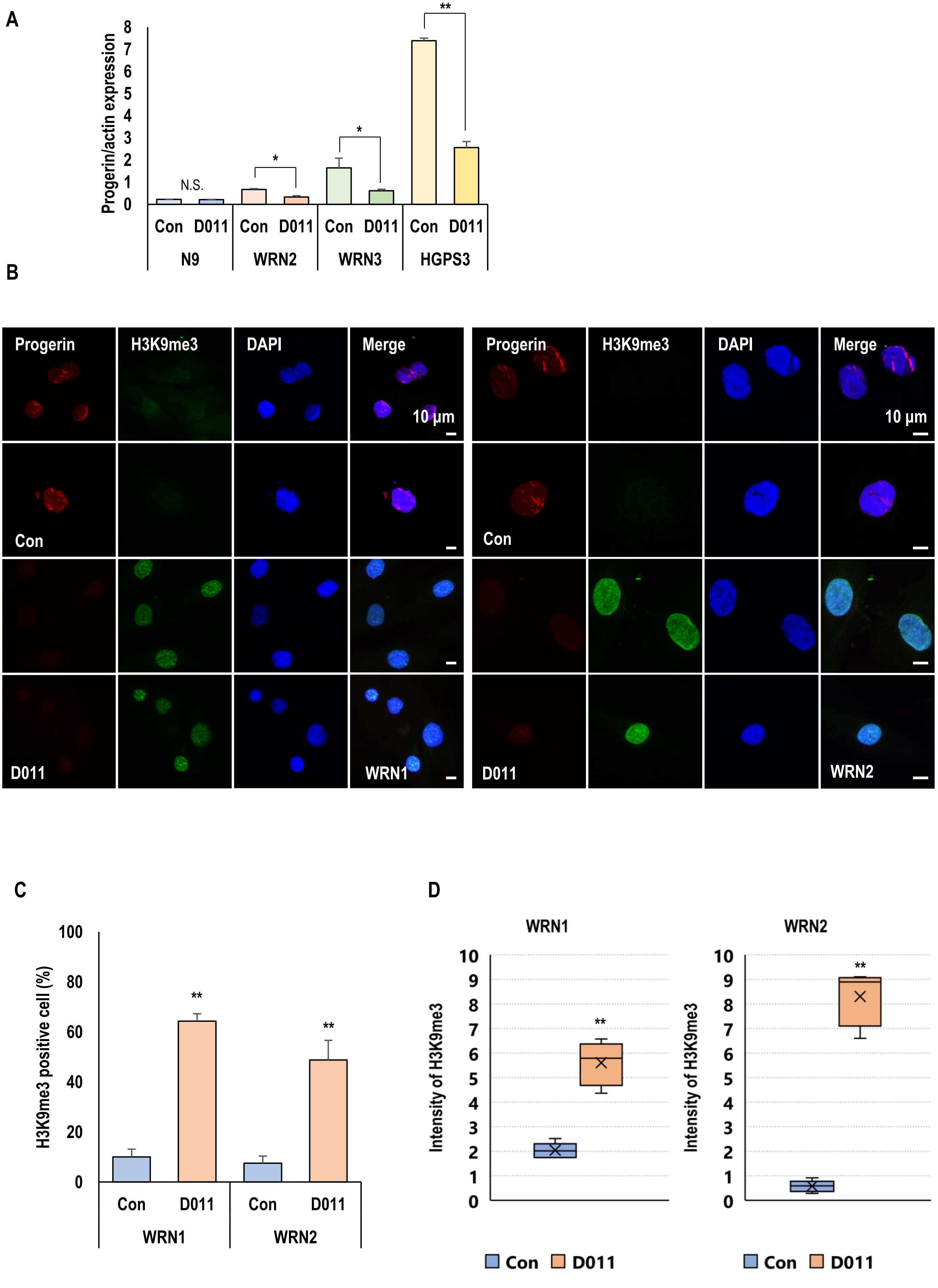
A
Progerin/actin expression
8
7
6
5
4
3
2
1
0
N.S.
*
*
**
Con D011 Con D011 Con D011 Con D011
N9 WRN2 WRN3 HGPS3
B
Progerin
H3K9me3
DAPI
Merge
10 μm
Con
D011
WRN1
Progerin
H3K9me3
DAPI
Merge
10 μm
Con
D011
WRN2
C
H3K9me3 positive cell (%)
100
80
60
40
20
0
**
**
Con D011 Con D011
WRN1 WRN2
D
WRN1
WRN2
Intensity of H3K9me3
10
9
8
7
6
5
4
3
2
1
0
**
**
Con D011
Con D011

Fig. S11. SLC-D011 ameliorates aging features of WRN cells. **A.** The graph shows progerin expression in N9, WRN, and HGPS cells after treatment with SLC-D011. **B.** SLC-D011 reduced the expression of progerin in WRN cells**.** Cells were stained with anti-progerin and anti-H3K9me3 antibodies and DAPI after treatment with SLC-D011 (n = 3 independent experiments; two-tailed Student's t-test). **C and D.** The box plots show the percentage of H3K9me3-positive cells **(C)** and the intensity of H3K9me3 expression **(D)** after treatment with SLC-D011 in WRN cells. All fibroblasts were examined at passage 11. $^{*}p < 0.05$, $^{**}p < 0.001$, N.S: not significant. Data are mean ± SD.

Figure S12

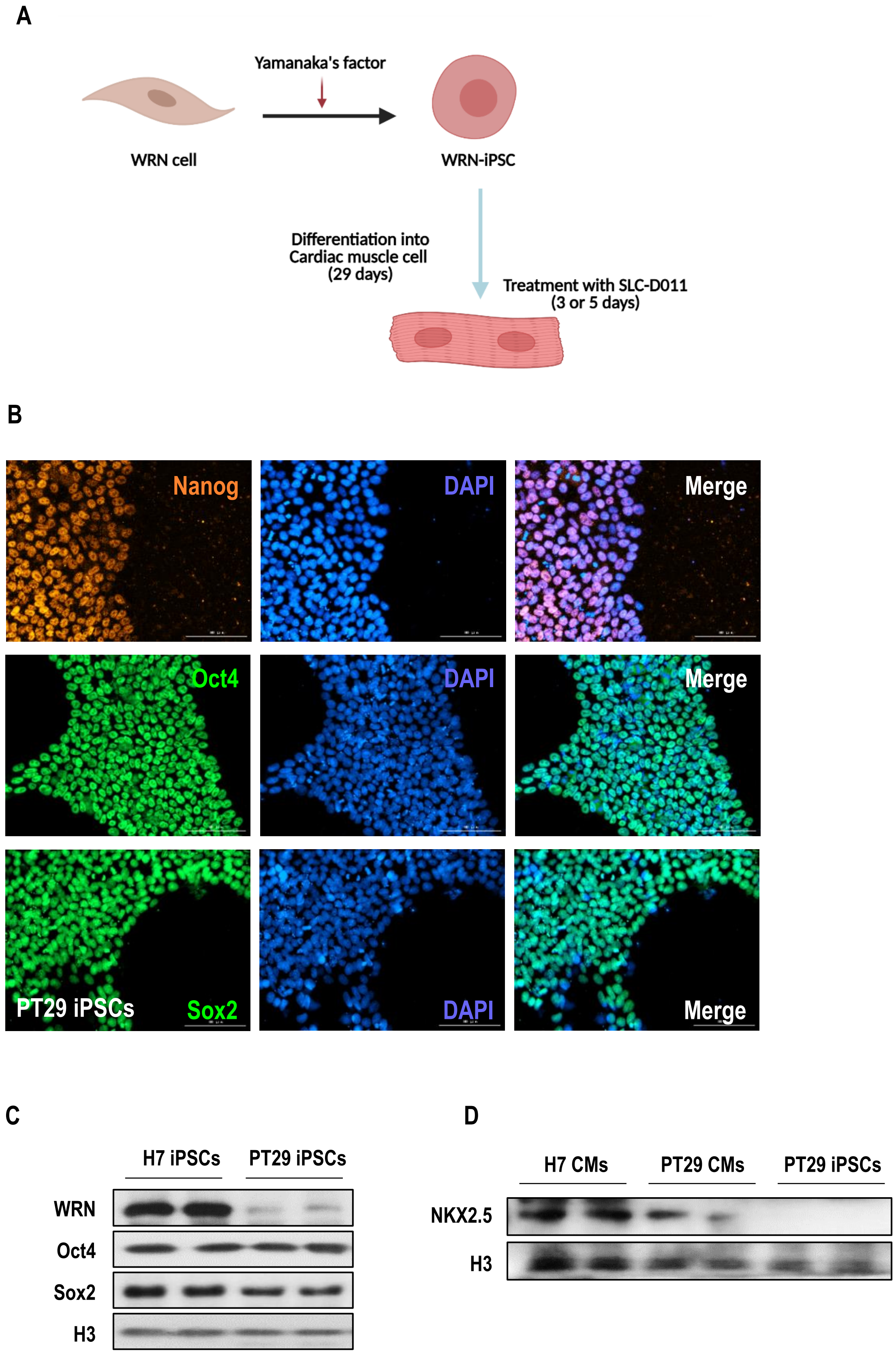
A
Yamanaka's factor
WRN cell
WRN-iPSC
Differentiation into Cardiac muscle cell (29 days)
Treatment with SLC-D011 (3 or 5 days)
B
Nanog
DAPI
Merge
Oct4
DAPI
Merge
PT29 iPSCs
Sox2
DAPI
Merge
C
H7 iPSCs
PT29 iPSCs
WRN
Oct4
Sox2
H3
D
H7 CMs
PT29 CMs
PT29 iPSCs
NKX2.5
H3

E

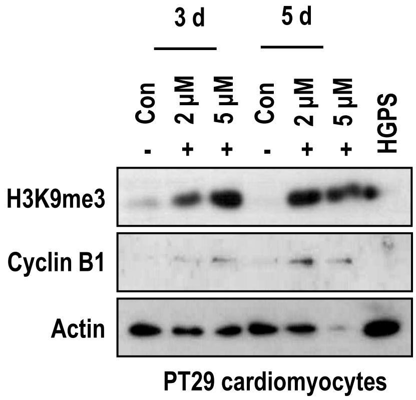
3 d
5 d
Con
2 µM
5 µM
Con
2 µM
5 µM
HGPS
-
+
+
-
+
+
H3K9me3
Cyclin B1
Actin
PT29 cardiomyocytes

F

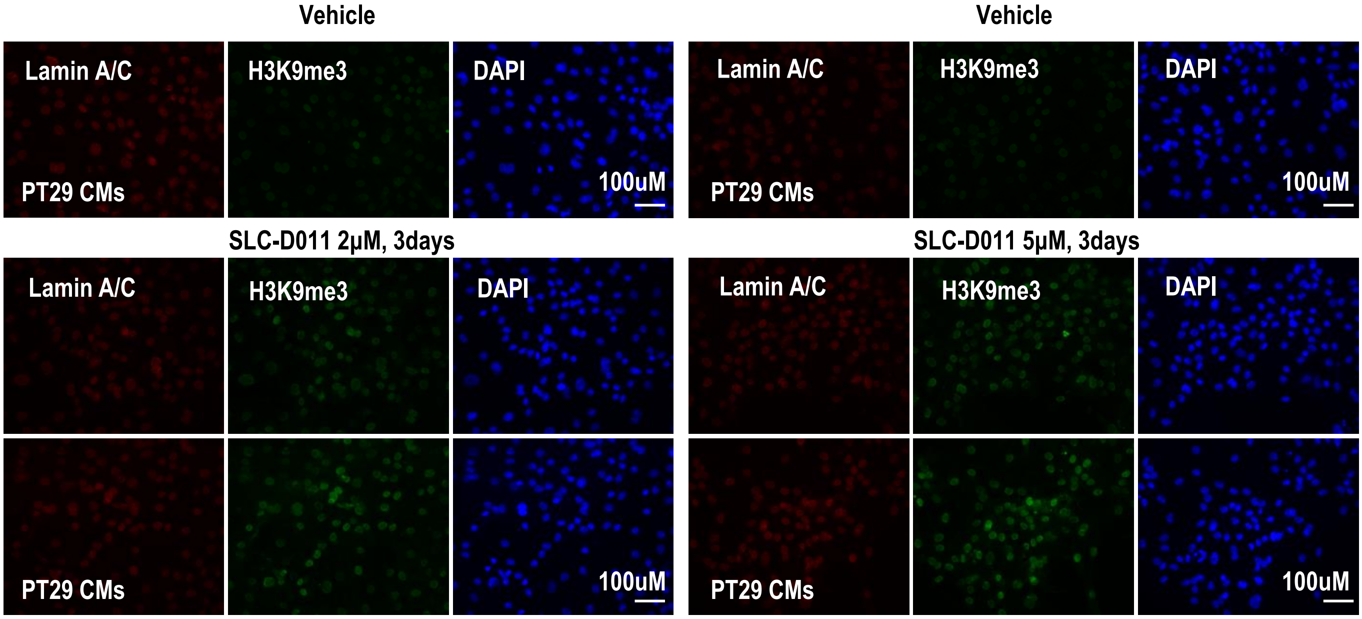
Vehicle
Vehicle
Lamin A/C
H3K9me3
DAPI
PT29 CMs
100uM
SLC-D011 2µM, 3days
SLC-D011 5µM, 3days
Lamin A/C
H3K9me3
DAPI
PT29 CMs
100uM

G

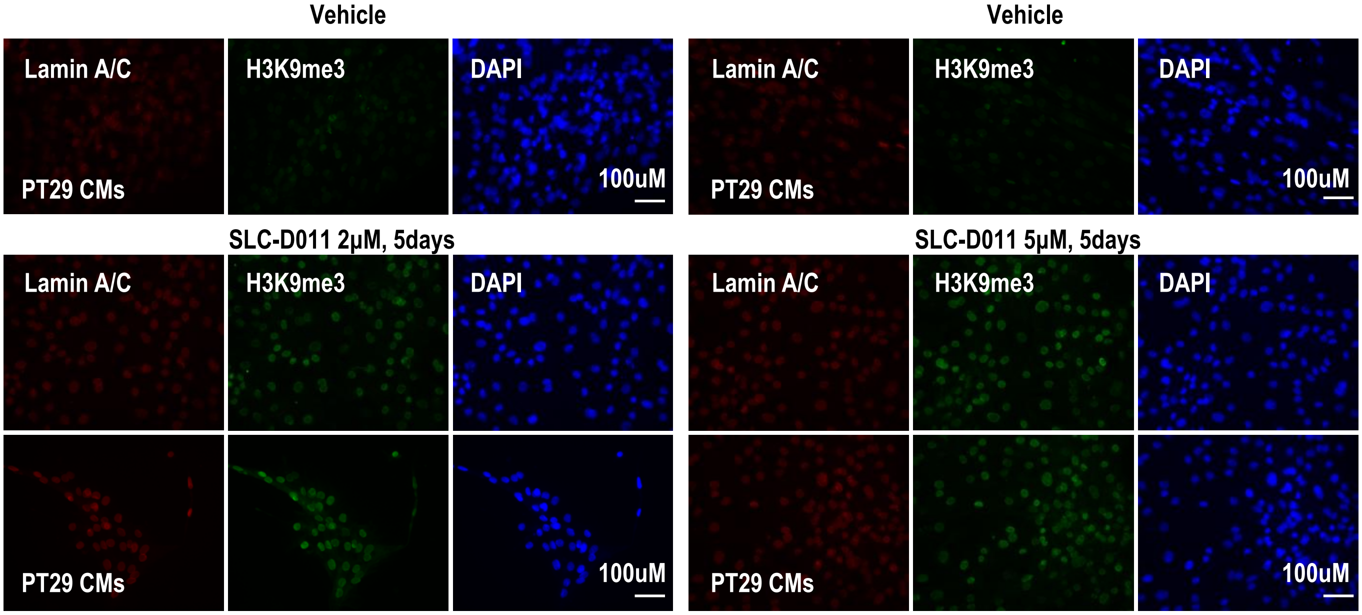
Vehicle
Vehicle
Lamin A/C
H3K9me3
DAPI
PT29 CMs
100uM
SLC-D011 2µM, 5days
SLC-D011 5µM, 5days
Lamin A/C
H3K9me3
DAPI
PT29 CMs
100uM

Fig. S12. SLC-D011 induces H3K9me3 expression in cardiomyocytes derived from WRN iPSCs. **A.** Generation of cardiomyocytes (CMs) derived from iPSCs using fibroblasts from a patient with WRN. **B.** Analysis of pluripotency was performed using ICC staining. Cells were stained with anti-Nanog, anti-Oct4, and anti-Sox4 antibodies and DAPI. **C.** Identification of iPSCs generated from a human embryonic stem cell line (H7) and WRN fibroblasts (PT29) by western blot. Both H7 iPSCs and PT29 iPSCs expressed Oct4 and Sox2. **D.** Identification of cardiomyocytes (CMs) derived from iPSCs. Expression of NKX2.5 was detected in H7 CMs and PT29 CMs but not in PT29 iPSCs. **E.** WRN iPSC-derived CMs were incubated with SLC-D011 for 3 days and 5 days after differentiation for 29 days. SLC-D011 induced H3K9me3 and cyclin B1 expression in CMs derived from WRN iPSCs. Blots were cropped from different parts of the same gels and analyzed by film-based imaging systems (n = 3 independent experiments; unpaired t-test). **F.** The expression of H3K9me3 in CMs derived from WRN iPSCs increased dose-dependently after treatment with SLC-D011 for 3 days. **G.** SLC-D011 does-dependently induce H3K9me3 expression in CMs derived from WRN iPSCs after 5 days of treatment. Cells were stained with anti-lamin A/C, anti-H3K9me3 and DAPI.

Figure S13

Fig. !F

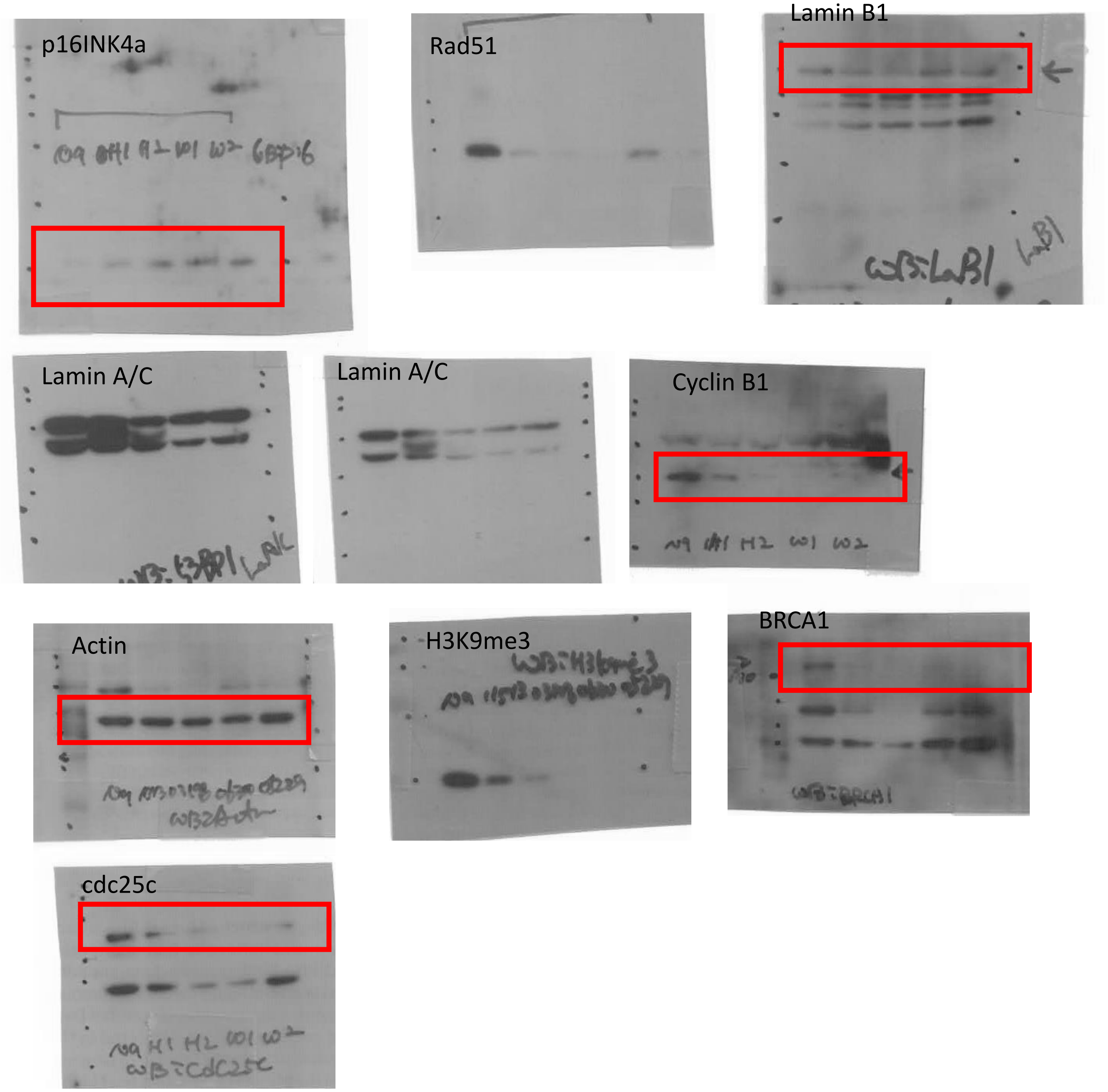

Fig. 3G

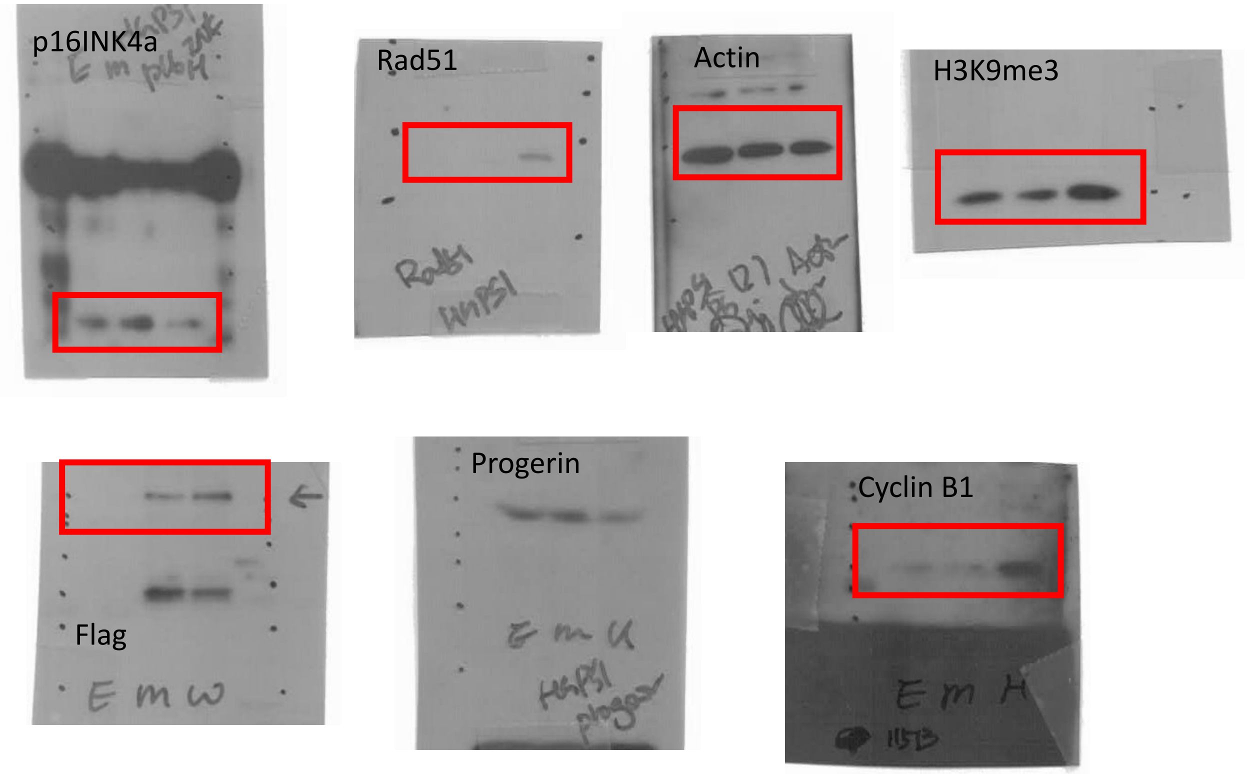

Fig. 4A

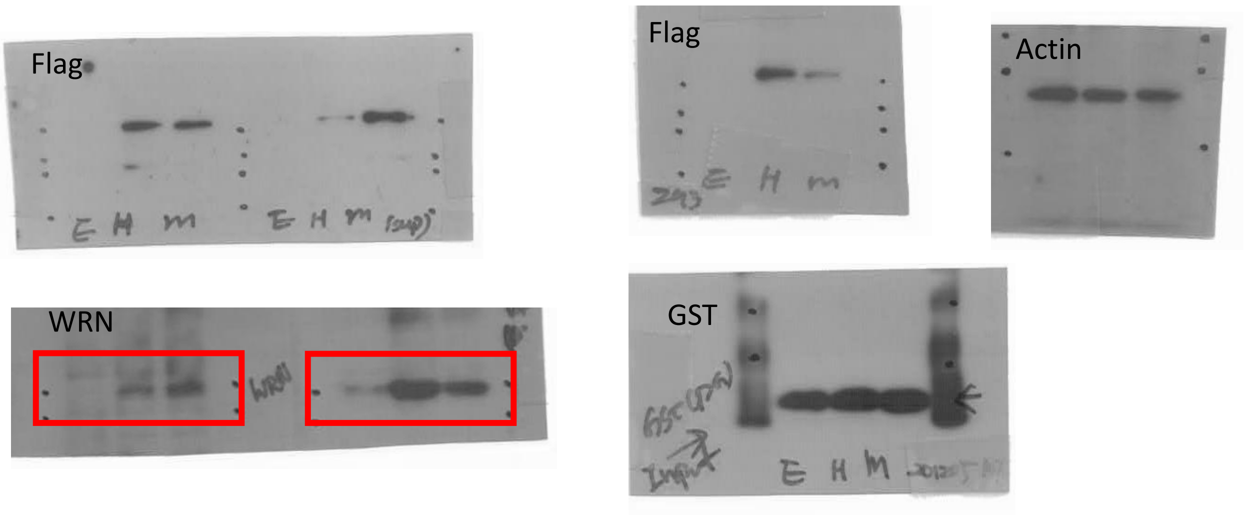

Fig. 4B

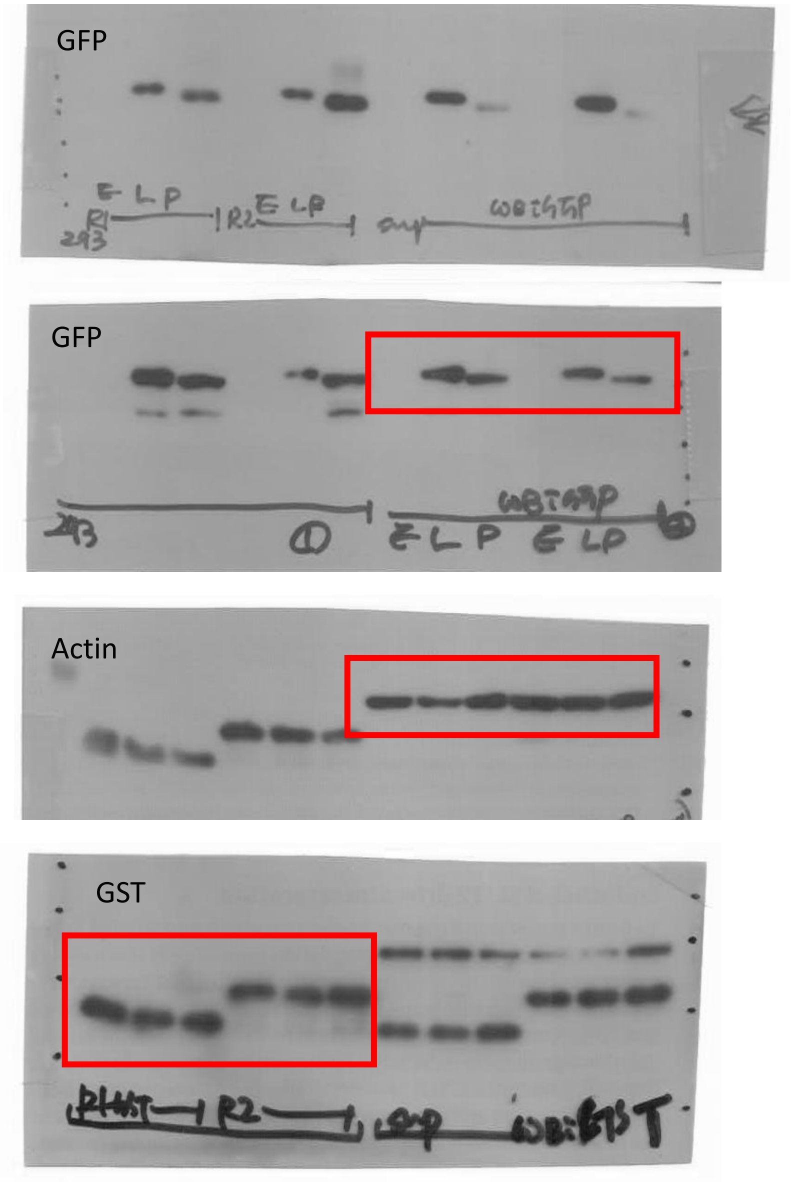

Fig. 4C

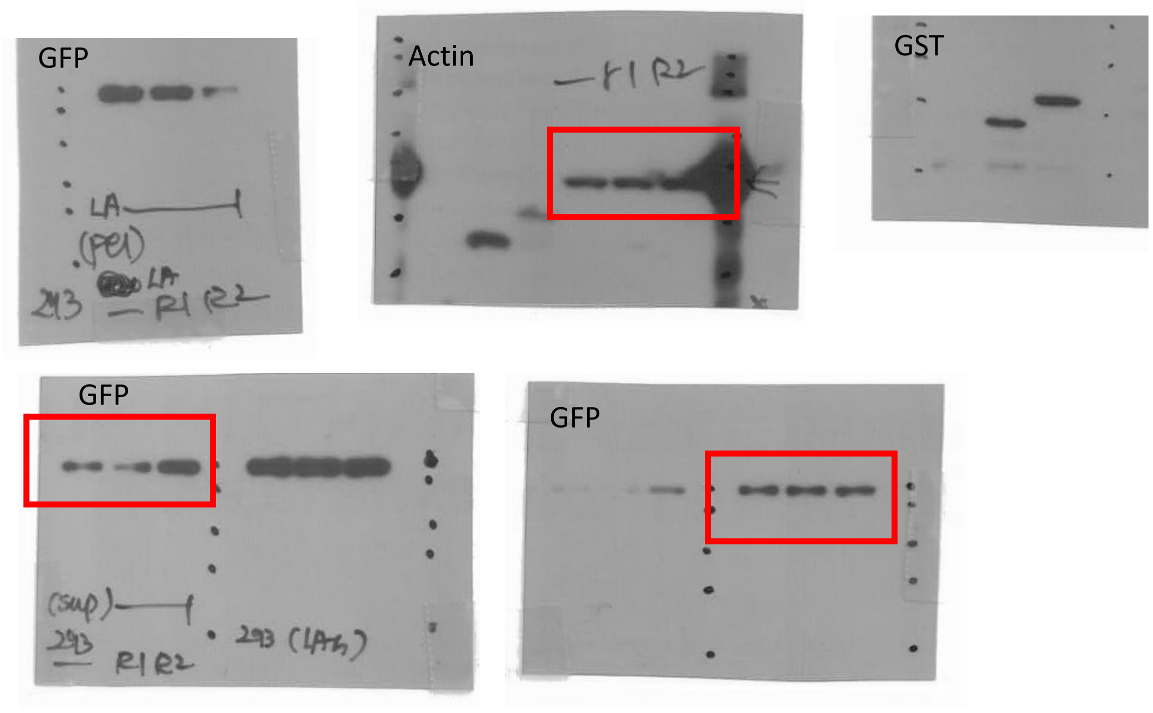
GFP
Actin
GST
GFP
GFP

Fig. 4G

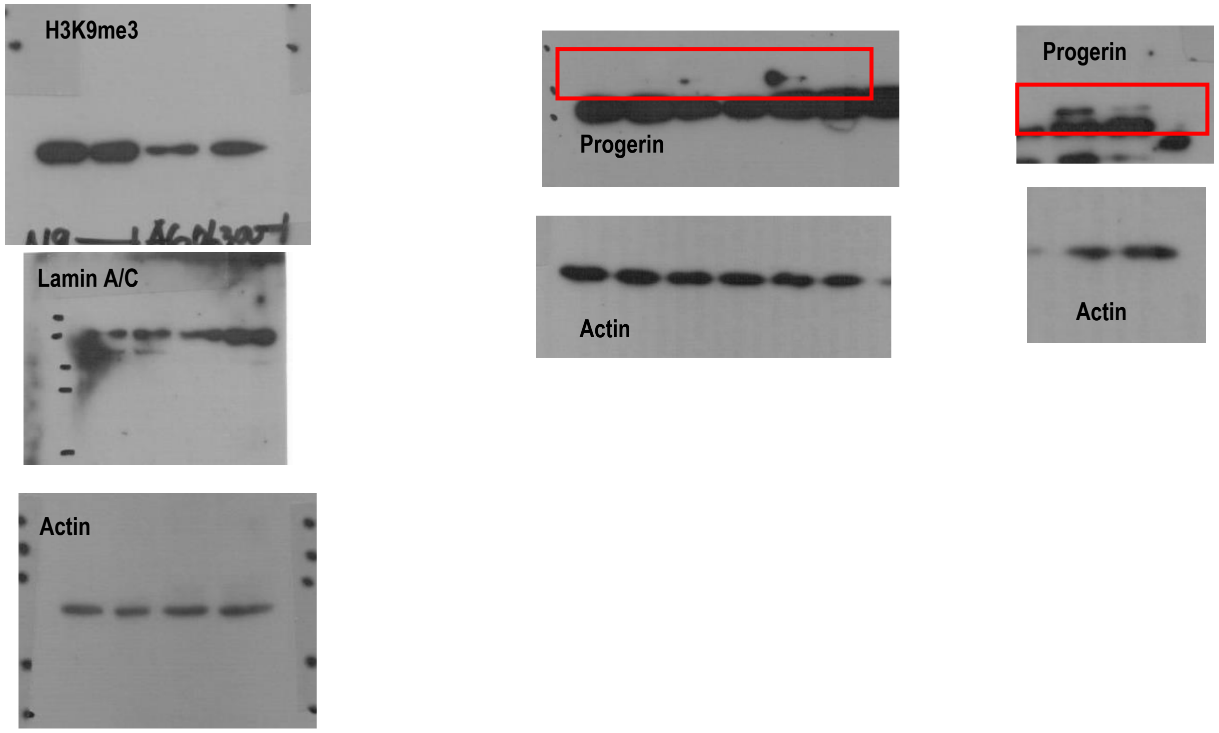
H3K9me3
Lamin A/C
Actin
Progerin
Actin
Progerin
Actin

Fig. S8E

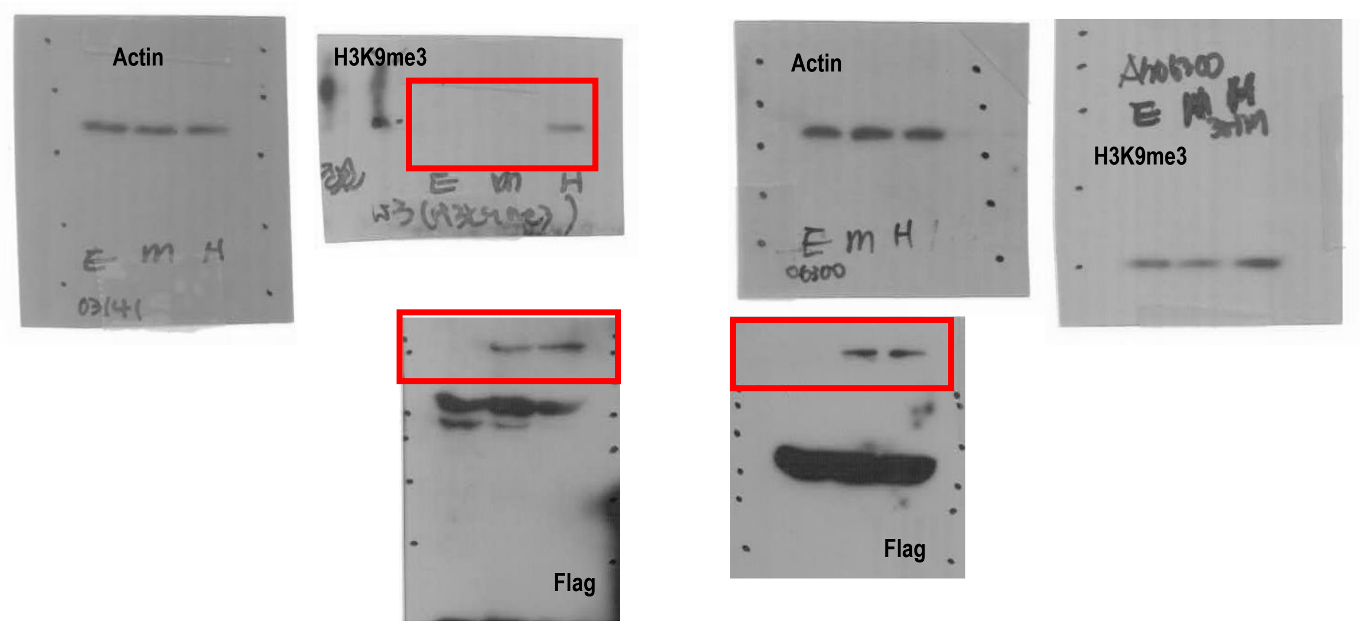

Fig. S10A

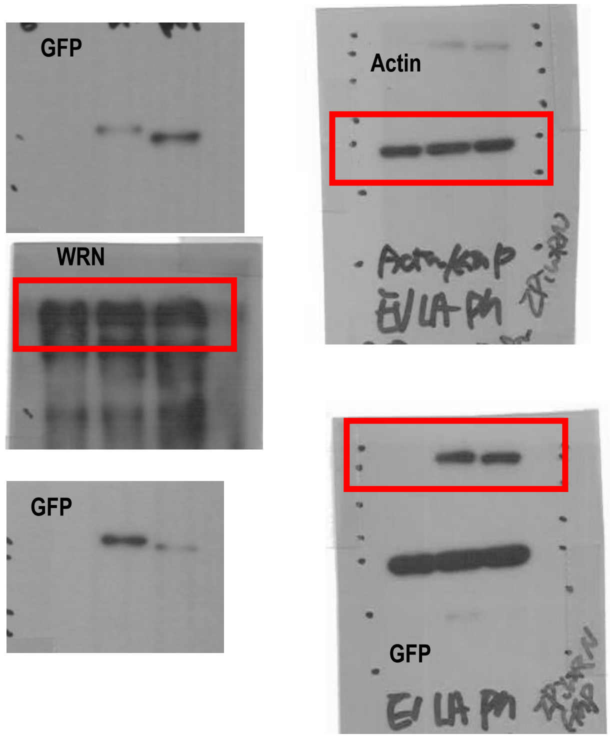

Fig. S12C

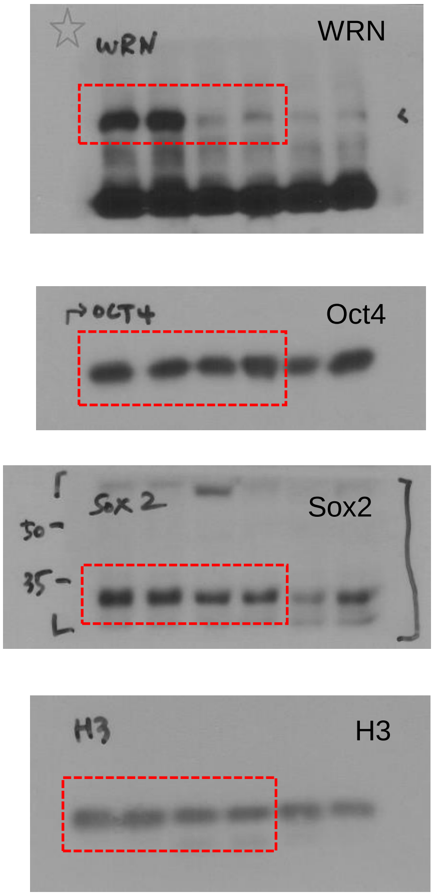

Fig. S12D

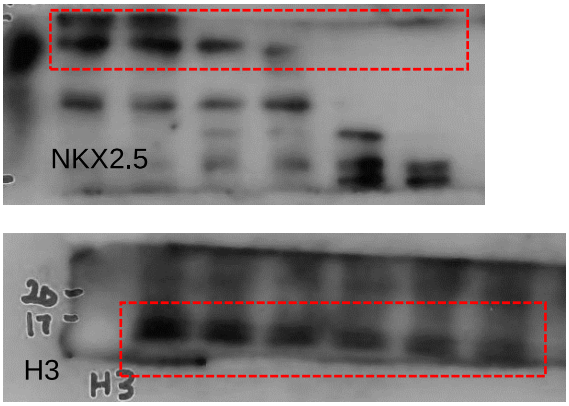

Fig. S12E

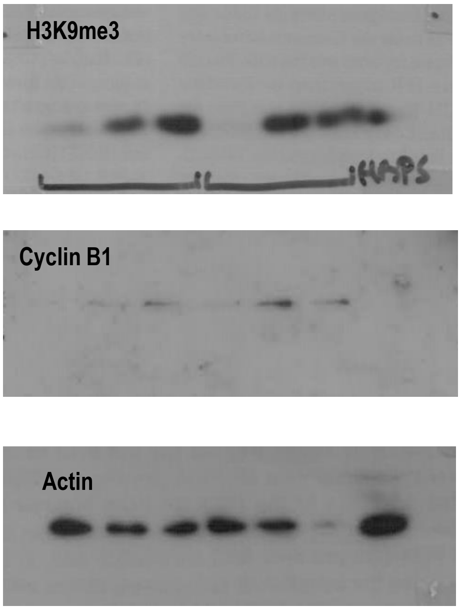

Fig. 2A

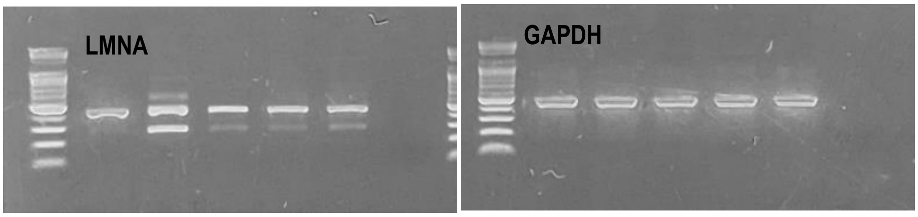

Fig. 4G

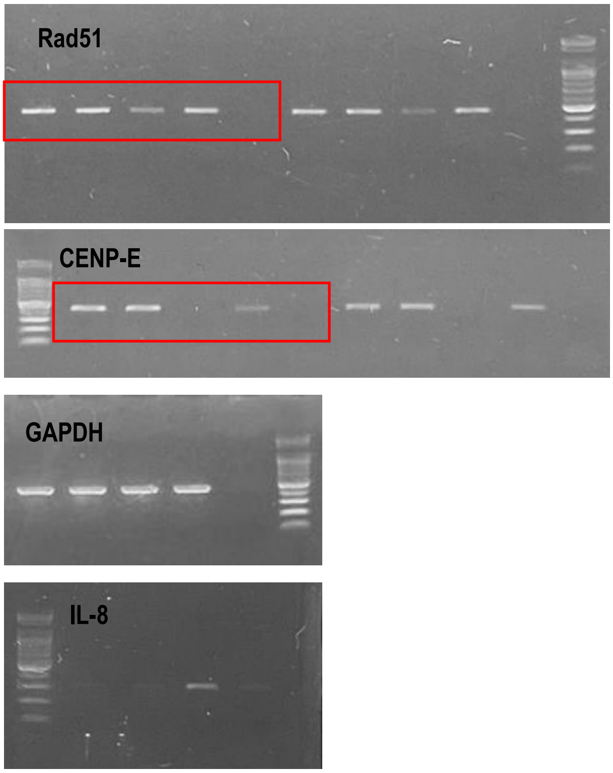

Fig. S1A

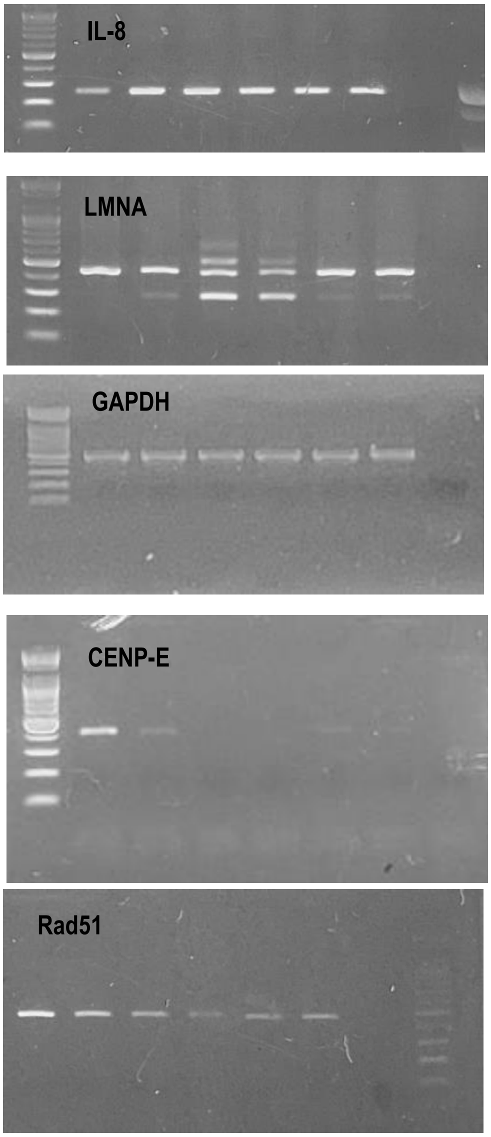

Fig. S4B

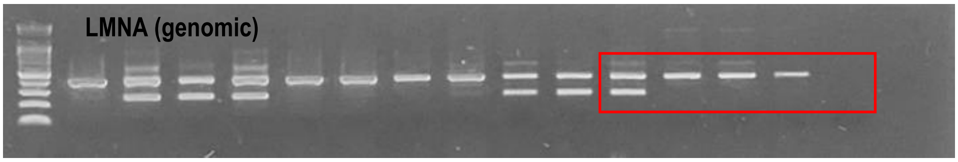

Fig. S4C

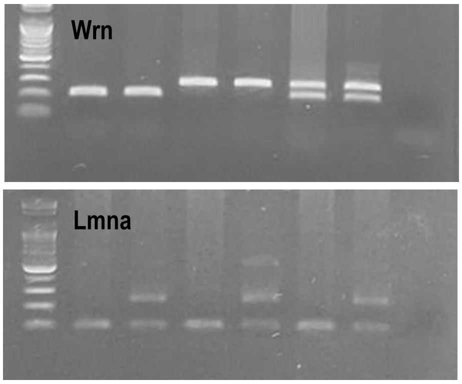

Fig. S5A

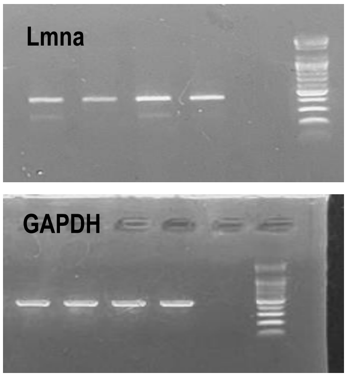

Fig. S10E

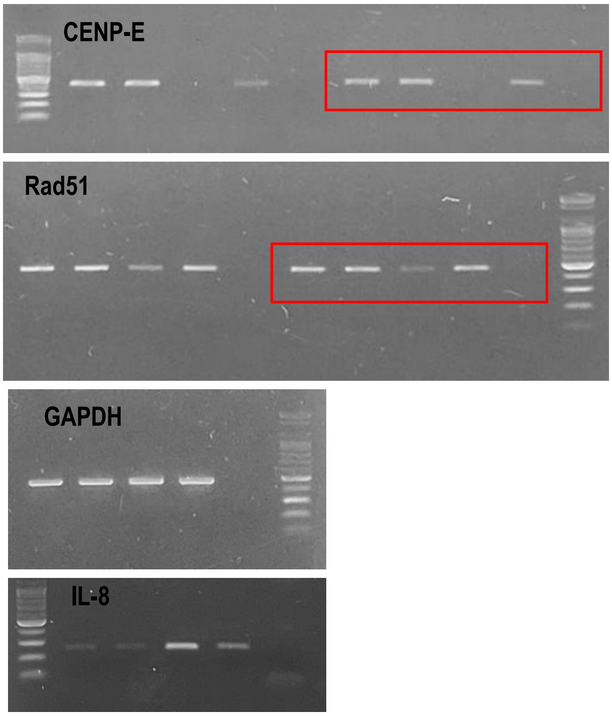

Supplement: Supplementary file 1 — Supplementary Information. [file 41598_2021_88325_MOESM1_ESM.pdf]
